# Photodissociation Dynamics of the Zn^+^(Acetylene) and Zn^+^(Ethylene) Cation‑π Complexes

**DOI:** 10.1021/acs.jpca.6c02947

**Published:** 2026-06-30

**Authors:** John R. C. Blais, Brandon M. Rittgers, Michael A. Duncan

**Affiliations:** Department of Chemistry, 1355 University of Georgia, Athens, Georgia 30602, United States

## Abstract

Zn^+^(acetylene) and Zn^+^(ethylene) ion–molecule complexes are investigated in the gas phase with selected-ion photofragment imaging. UV photodissociation produces respectively Zn^+^ and C_2_H_2_^+^ or Zn^+^ and C_2_H_4_^+^ fragment channels, revealing both simple bond cleavage and charge-transfer dissociation in these complexes. Imaging of each fragment channel reveals considerable kinetic energy release (KER), which provides upper limits on the bond dissociation energies (BDEs): *D*_0_ ≤ 1.04 ± 0.20 eV (24.0 ± 4.6 kcal/mol) for Zn^+^-(C_2_H_2_) and *D*_0_ ≤ 0.82 ± 0.18 eV (18.9 ± 4.2 kcal/mol) for Zn^+^-(C_2_H_4_). Agreement with previous data from spectroscopic measurements on Zn^+^(C_2_H_4_) suggest that these upper limits are near the true BDE for each of these complexes. Density functional theory (DFT) calculations explore the bonding and structures of the Zn^+^(C_2_H_2_) and Zn^+^(C_2_H_4_) ions, employing the B3LYP, M06, M06-L, and MN15-L functionals. Time-dependent DFT (TD-DFT) computations at the B3LYP/def2-QZVP level characterize the excited states of these complexes. Zn^+^(C_2_H_2_) dissociates via absorption to the bound ^2^B_2_ excited state followed by curve crossing to the ^2^A_1_ charge-transfer excited state, whereas Zn^+^(C_2_H_4_) dissociates via direct excitation of the charge-transfer state.

## Graphical abstract

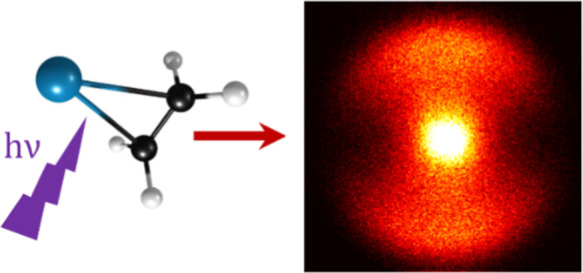

## Introduction

Cation–π interactions are widespread throughout chemistry and biology.^1–8^ Examples vary from simple organometallic systems such as metal ion–benzene complexes to side-chain interactions between amino groups and aromatic residues in proteins. Metal ion-acetylene and metal ion-ethylene complexes in the gas phase serve as perhaps the simplest models of this interaction. Mass spectrometry studies of these systems reveal not only the formation of cation-π complexes,^9–22^ but also the possibility of reactions in multiligand complexes promoted by the metal cations.^23–40^ One particularly fascinating reaction is the cyclotrimerization of acetylene to form benzene.^23–40^ Infrared spectroscopy experiments and theory suggest that early transition metals are more effective in promoting this chemistry,^41–53^ while zinc cations were found instead to promote the formation of poly-acetylene chain structures.^48^ Metal ion-acetylene and -ethylene complexes have also been studied previously with electronic spectroscopy, revealing cation–π structures with particularly weak bonding.^54–62^ Here, we investigate this bonding further using photofragment imaging measurements.

Metal ion-acetylene and metal ion-ethylene systems have been studied extensively using mass spectrometry techniques which explore their gas-phase reactions and thermochemistry.^5–36^ Computational studies have been performed to examine structures, bonding, and spectra for these complexes.^11,13,18,20–22,30,33,34,40,44–53^ The metal ion-ligand bond dissociation energy (BDE) has been determined for many of these systems using techniques such as collision-induced dissociation (CID) or temperature-dependent equilibrium studies. Armentrout and co-workers have determined the BDEs for many of the first-row transition metal ion complexes with ethylene or acetylene using CID.^14,17,19^ Other experiments have used photodissociation thresholds^18^ or energetic cycles using measured spectroscopic constants^54–62^ to investigate BDEs. Typical values of the M^+^-(C_2_H_2_) BDEs for transition metals are in the range of 1.6–2.6 eV, while those for M^+^-(C_2_H_4_) complexes are 0.9–2.0 eV. Kleiber and co-workers have reported a study of the photodissociation spectroscopy of Zn^+^-(C_2_H_4_) in the region of 220–550 nm, and have employed a Birge–Sponer plot of an extended progression in that spectrum to obtain the bond energy.^54^ They determined that the ground state BDE is 0.86 eV,^54^ which was considerably lower than their computed value of 1.144 eV. To our knowledge, the BDE for Zn^+^-(C_2_H_2_) has yet to be measured, but its calculated value (1.16 eV) is also much less than those of other transition metal ion systems.^48^

The structures of metal-ion acetylene complexes and metal-ion ethylene complexes have been predicted to have C_2v_ symmetry, with the metal cation sitting directly over the midpoint of the C–C bond. More specifically, for the metal-ion ethylene complexes the metal cation is directly above the plane formed by the ethylene atoms. This kind of organometallic bonding is typically described by the Dewar–Chatt–Duncanson (DCD) framework.^5,63–65^ In this model, a bond forms between the metal and alkene/alkyne by σ-donation of electron density into metal orbitals from the π orbitals, and back-donation of electron density from filled metal d orbitals into the alkene/alkyne π* orbitals. These charge-transfer interactions tend to weaken the bonding and reduce the frequencies of the C–H vibrations. Additionally, as back-donation increases, the acetylene/ethylene hydrogens bend further away from the metal cation in the C_2v_ structure. Interestingly, a study by our group on the IR spectroscopy of the Zn^+^(C_2_H_2_) complex revealed that its C–H vibrations were hardly shifted from those of acetylene.^48^ Apparently, the charge transfer in this complex is small because of the filled valence d orbitals and half-filled s orbital of Zn^+^ (configuration: [Ar]­3d^10^4s^1^).

An intriguing aspect of the photochemistry in certain organo-metallic ions is the observation of charge-transfer photodissociation.^66–74^ When the ionization potential (IP) of the metal atom is lower than that of the ligand, the charge in an ion–molecule complex resides on the metal in the ground state. Collisional dissociation takes place in the ground state, eliminating the metal ion as the fragment. However, in cases when the ionization energies of the metal and ligand are close, photoexcitation can access a charge-transfer excited state, with dissociation producing the charged ligand as the fragment. Our group has documented several examples of such charge-transfer photochemistry.^66–74^ Kleiber and co-workers have also reported excited state charge transfer in their spectrum of Zn^+^(C_2_H_4_).^54^ Dissociation in such excited states often produces kinetic energy in the fragments, which can be measured with photofragment imaging. An energetic cycle accounting for dissociation on this excited state surface provides an upper limit on the BDE of the complex. Our group has utilized this approach to examine several metal cation–π systems.^70–74^ In the present work, we extend this methodology to the Zn^+^(C_2_H_2_) and Zn^+^(C_2_H_4_) systems. Computational studies were performed using density functional theory (DFT) with different functionals to compare their accuracy in describing the bonding of these systems. Time-dependent DFT (TD-DFT) was also employed to examine the excited states of each complex.

## Methods

A molecular beam containing cation–molecule complexes of Zn^+^(C_2_H_2_)_*n*_ or Zn^+^(C_2_H_4_)_*n*_ was generated from the laser ablation^75^ of a zinc metal rod in a pulsed supersonic expansion of argon containing 3% of acetylene or ethylene. The vaporization wavelength used was the third harmonic (355 nm) of a Nd/YAG laser (Continuum Surelite-II 10), at 10–20 mJ/pulse. The molecular beam was collimated with a skimmer and ions were extracted into a reflectron time-of-flight mass spectrometer (RTOF-MS) designed for mass analysis and photodissociation product analysis experiments.^76,77^ Specific ions were mass-selected using pulsed deflection plates in the initial flight tube before photodissociation. These ions were irradiated with either the third (355 nm) or fourth harmonic (266 nm) of a Nd/YAG laser (Continuum Surelite-EX I) at 2 mJ/pulse in the turning region of the reflectron, and fragment mass analysis was based on the transit time through a second flight tube. Photodissociation “difference” mass spectra were obtained by subtracting a mass spectrum with the laser off from one with it on. These experiments identify the photofragments formed at different wavelengths.

Photofragment imaging studies were performed with our selected-ion velocity-map imaging (SI-VMI) apparatus to measure the kinetic energy release, if any, in different photofragments at different wavelengths.^70^ This instrument employs concepts developed previously for the imaging of photodissociation in neutral molecules,^78–82^ and adapts them to the study of mass-selected ions. In this configuration, the reflectron region of the RTOF-MS was grounded and selected ions traveled through it into a linear flight tube for imaging. This includes a deceleration field followed by ion optics designed for velocity-map imaging (VMI).^83^ After deceleration, mass-selected ions were excited with a Nd/YAG laser (Continuum Surelite EX-1) using either 10 mJ/pulse of the third harmonic at 355 nm or 5 mJ/pulse of the fourth harmonic at 266 nm. The resulting photofragments were reaccelerated by the VMI electrode assembly into a 1.0 m flight tube, with a dual MCP/P-47 detector (Beam Imaging Solutions BOS-75) at its end. The detector was activated by pulses with a fast rise time high-voltage pulser (DEI PVX-4140) to conduct DC-slice imaging^84^ and detect the middle ∼90 ns of the photofragment ion cloud. The images were collected by a CCD camera (Edmund Optics) and centroiding was accomplished with the NuACQ software package.^85,86^ Image analysis was achieved using the FinA program^87^ with resolution determined from the image of Ar^+^ from Ar_2_^+^ at identical instrument settings.^88^ The SI-VMI instrument is unique to our group,^70–74^ but similar instruments have been described by others.^89–93^

Computational chemistry calculations on Zn^+^(C_2_H_2_), Zn^+^(C_2_H_4_), and their cationic and neutral fragments, were conducted with the Gaussian 16 program package.^94^ Calculations employed DFT with the B3LYP, M06, M06-L, and MN15-L functionals, all using the def2-TZVP basis set.^95^ These calculations determined the geometries of these complexes and their energetics. Excited electronic states were simulated with time-dependent density functional theory (TD-DFT)^96^ utilizing the B3LYP functional with the def2-QZVP basis set.

## Results and Discussion

### Mass Spectra

The mass spectra of Zn^+^(C_2_H_2_)_*n*_ and Zn^+^(C_2_H_4_)_*n*_ cluster ions are presented in Figure 1. All peaks assigned to species containing Zn^+^ are triplets, representing the three most stable isotopes of zinc at 64, 66, and 68 amu. Both spectra exhibit clustering of up to 10 ligands around the Zn^+^ center. The spectra have additional masses consistent with pure clusters of the hydrocarbon ligands, up to (C_2_H_2_)_5_^+^ in the case of acetylene and (C_2_H_4_)_12_^+^ in the case of ethylene. The ethylene molecule appears to have a higher propensity for clustering compared to acetylene under similar conditions. Although the expansion gas is argon, we do not see any significant concentrations of the mixed ions containing argon which were studied in our previous work.^48^ This is attributed to slightly different cluster source configurations that were optimized for the production of the Zn^+^(C_2_H_2_) or Zn^+^(C_2_H_4_) parent ions. It should be noted that the mass of ^64^Zn^+^Ar is equal to that of (C_2_H_2_)_4_^+^, and the mass of ^64^Zn^+^(C_2_H_2_)Ar equals that of (C_2_H_2_)_5_^+^. However, the presence of (C_2_H_2_)_2_^+^ and the lack of the zinc isotopic structure in the assigned (C_2_H_2_)_*n*_^+^ mass peaks confirms that these are pure acetylene clusters.

**Figure 1:**
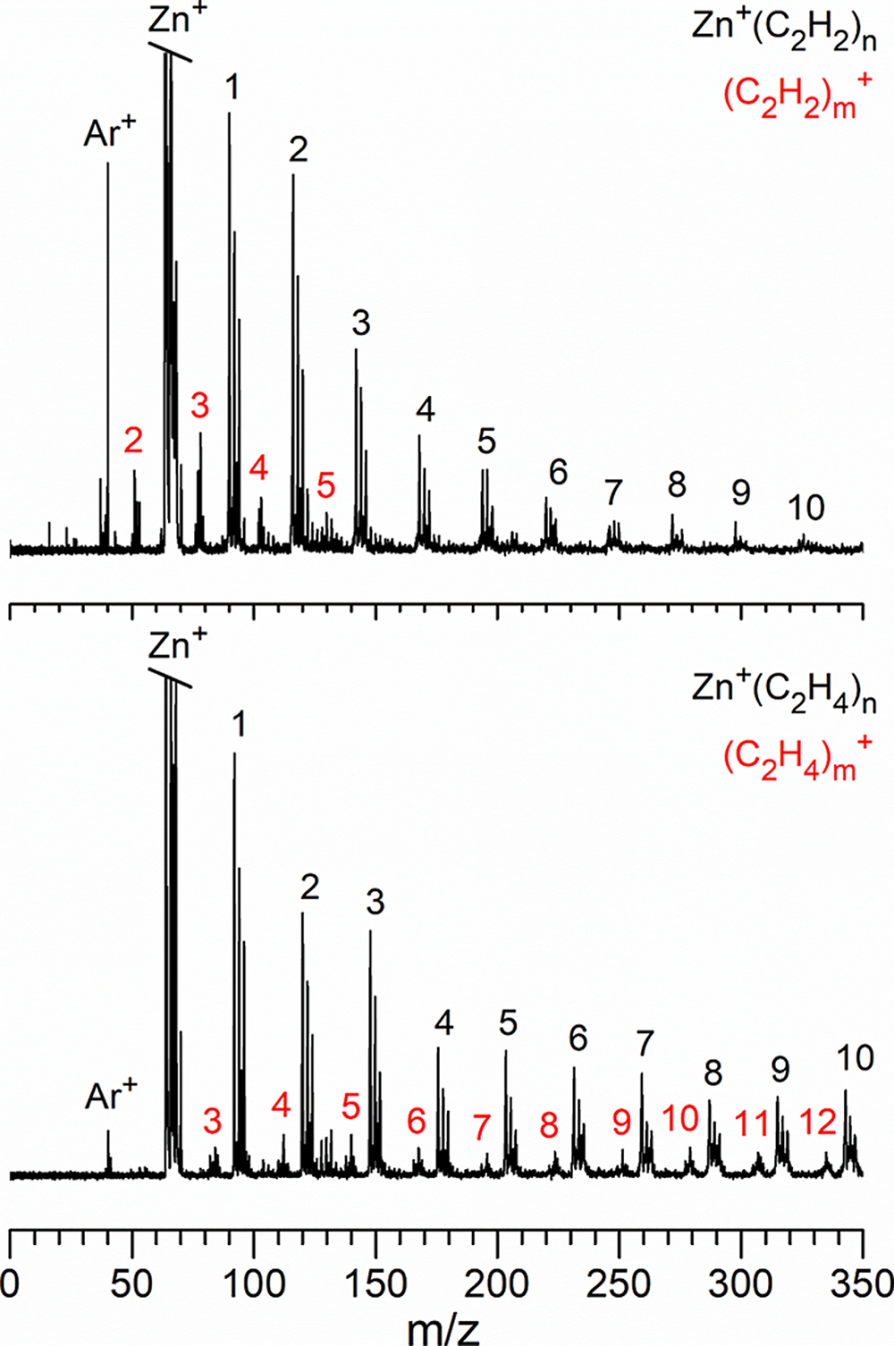
Mass spectra of molecular beams generated from laser ablation of a zinc metal rod during the pulsed supersonic expansion of argon gas seeded with 3% acetylene (top) and 3% ethylene (bottom).

Figure 2 shows the photodissociation mass spectra of the Zn^+^(C_2_H_2_) and Zn^+^(C_2_H_4_) species. These spectra are obtained by taking the difference between a mass spectrum with the photodissociation laser “on” and one with it “off”. The parent ion depletion is represented as a negative intensity and the fragment ions are positive-going. Zn^+^(C_2_H_2_) did not photodissociate at 355 nm. At 266 nm, the Zn^+^(C_2_H_2_) ions fragmented into both Zn^+^ + C_2_H_2_ and C_2_H_2_^+^ + Zn channels. Zn^+^(C_2_H_4_) fragmented into both Zn^+^ + C_2_H_4_ and C_2_H_4_^+^ + Zn at 355 and 266 nm. Based on peak intensity, the branching ratios for these fragments appeared constant over the range of 2–10 mJ/pulse for the photodissociation laser. The overall signal level for the photodissociation of the Zn^+^(C_2_H_2_) ions is about half of that for the Zn^+^(C_2_H_4_) ions. In previous work, Kleiber and co-workers found both Zn^+^ and C_2_H_4_^+^ ions in the photodissociation of the Zn^+^(C_2_H_4_) complex, with a branching ratio that varied with the wavelength.^54^ However, the C_2_H_4_^+^ ion was generally more intense than the Zn^+^ ion in their data. The weak ^66^Zn^+^ signal and the lack of the ^68^Zn^+^ in our spectra are due to the influence of the mass-selection deflection plates on the trajectory of the Zn^+^(C_2_H_2_) and Zn^+^(C_2_H_4_) parent ions, causing these ions to miss the detector. The timing of the pulsed photodissociation laser adds another level of mass selection, which in these cases was optimized to favor the ^64^Zn^+^ ions.

**Figure 2:**
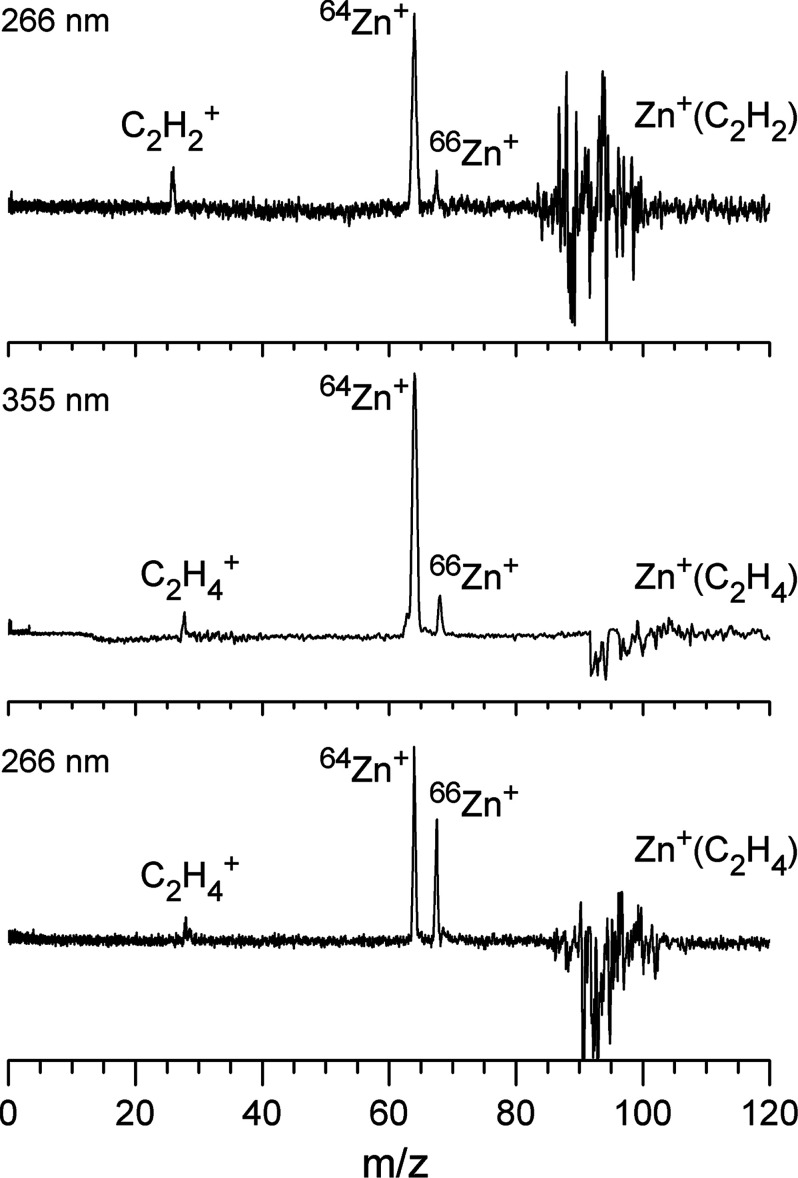
Photodissociation difference mass spectra of the Zn^+^(C_2_H_2_) (2 mJ/pulse of 266 nm (top)) and Zn^+^(C_2_H_4_) (2 mJ/pulse of 355 nm (middle) and 2 mJ/pulse of 266 nm (bottom)) ion–molecule complexes. Both parent ions demonstrate a fragmentation channel corresponding to dissociative charge transfer. The 64 vs 66 isotope ratios and the absence of the 68 isotope are caused by the mass gate aperture and the timing of the photodissociation laser pulse, which both introduce mass discrimination.

The photofragments detected here are understandable based on the electronic states and ionization energies of zinc, acetylene and ethylene. Each of these have few low-lying electronic states, and therefore both the Zn^+^(C_2_H_2_) and Zn^+^(C_2_H_4_) complexes also have only a few excited electronic states accessible in the near-UV. For example, three excited states were assigned in the spectroscopy of Zn^+^(C_2_H_4_) by Kleiber and co-workers.^54^ The charge-transfer dissociation channel is of primary interest here, since it often produces large kinetic energy release in the fragments. In the past, charge transfer dissociation was observed most often in transition metal ion complexes with aromatic ligands such as benzene (IP = 9.24 eV).^66–74^ Most transition metals have ionization energies in the 7–8 eV range,^97^ which means that charge-transfer resonances occur in the blue visible or near-UV region. However, the ionization energies of acetylene (11.4 eV) and ethylene (10.51 eV) are much higher,^97^ changing the energetics significantly. Conveniently, the ionization energy of zinc is the highest of all the first-row transition metals at 9.39 eV, bringing the IP differences in the present complexes to the range of ΔIP = 2.01 eV for Zn^+^(acetylene) and 1.12 eV for Zn^+^(ethylene). Observation of the charge transfer photodissociation channel requires a photon energy that overcomes both the bond energy and the IP difference. Therefore, these channels can be detected at near-UV wavelengths. Furthermore, Kleiber and co-workers have characterized the low-lying electronic states of the Zn^+^(ethylene) complex, and recorded charge transfer fragmentation at photon energies as low as 4.17 eV (297 nm).^54^ Based on their spectroscopy, the Nd/YAG laser harmonics at 355 and 266 nm should access the charge-transfer excited states expected to provide kinetic energy release, and so we investigate these wavelengths for imaging experiments.

### Zn^+^(C_2_H_2_) Photofragment Images

Figure 3 shows the images of the C_2_H_2_^+^ and Zn^+^ fragments from photodissociation of Zn^+^(C_2_H_2_) at 266 nm and the corresponding kinetic energy release (KER) spectra. No photodissociation was detected for this ion at 355 nm. Because the photodissociation efficiency is rather low, these images for Zn^+^(C_2_H_2_) took about twice as long to accumulate as those for Zn^+^(C_2_H_4_) shown below. These images are symmetrized to compensate for a “dead spot” on the MCP detector; the corresponding raw images are presented as Figures S1 and S2 in the Supporting Information. The KER spectra were derived from the raw images. The KER spectra are obtained by summing the intensity at each radius of the image, which is assigned a value of KER based upon the velocity calculated from detector size and detected ion flight time. The outer edges of the images are therefore generated by fragment ions which possess the maximum kinetic energy (KER_max_), and the filled inner portion of the image corresponds to ions with lower kinetic energies. Because of energy conservation, this lower KER signal necessarily represents ions with energy contained in other degrees of freedom, such as vibrational and rotational states of the molecular fragments. Both of the images shown here have the majority of their signal at lower kinetic energies, indicating that photodissociation produces a broad distribution of excited internal states of the fragments.

**Figure 3:**
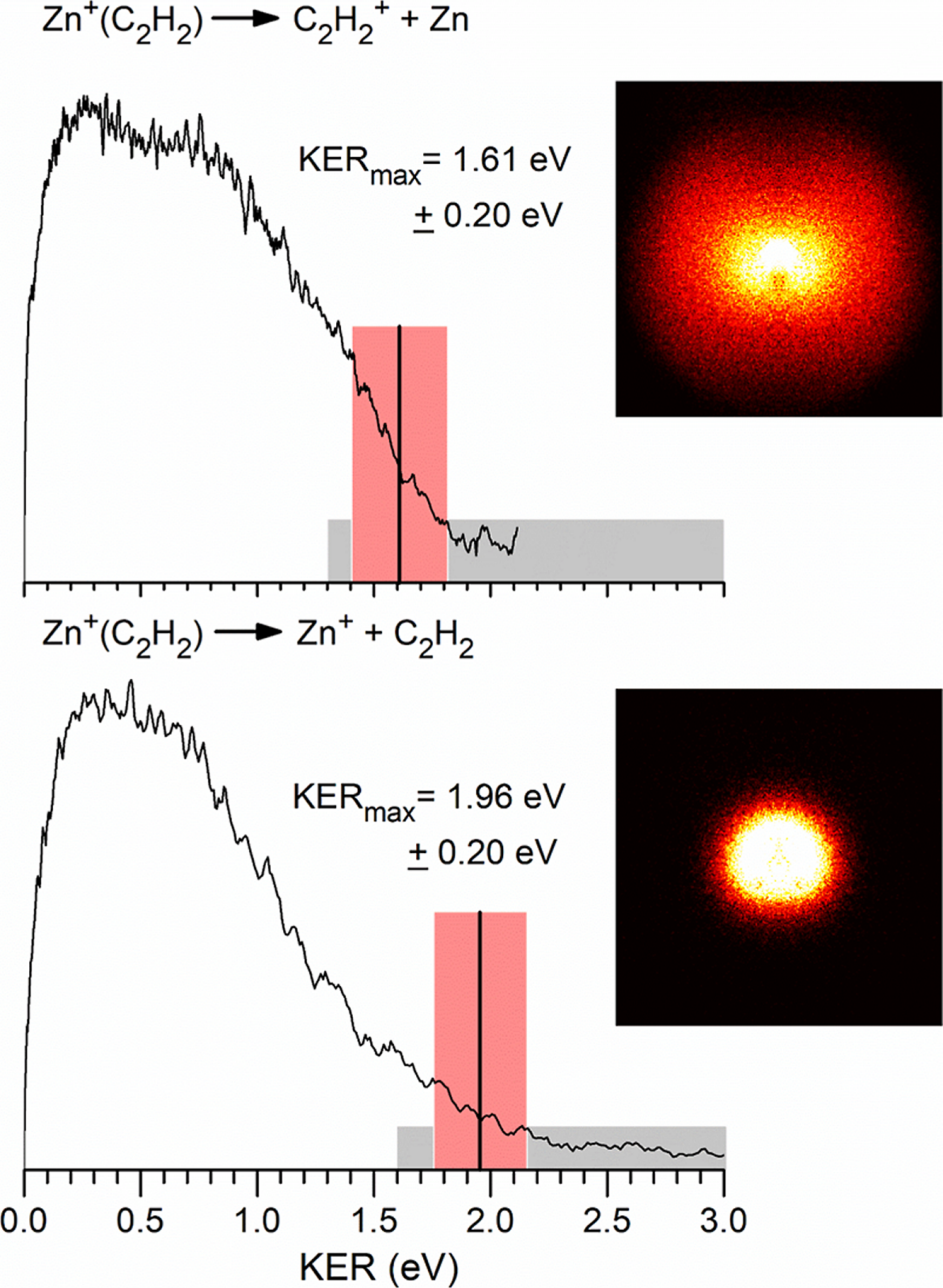
Symmetrized photofragment images with kinetic energy release spectra of the C_2_H_2_^+^ fragment (top) and the Zn^+^ fragment (bottom) from Zn^+^(C_2_H_2_) photodissociation at 5 mJ/pulse of vertically polarized 266 nm.

The KER_max_ values, taken from the outside edges of these images, provide information about the dissociation energies of these ions. Although the distribution of energies is broad, the outside edge of the image indicates that at least some of the ions dissociate with this much excess kinetic energy. The photon energy must therefore exceed the bond energy and this KER_max_ energy. Rearranging this, the dissociation energy must be less than the photon energy minus this KER value

1 $$D_{0}\leq h\nu -KER_{\max}$$

As written, this assumes dissociation on the ground state surface. For the charge-transfer channel, the photon energy must exceed the bond energy, the ionization energy difference and the KER. This results in the limit on the dissociation energy of

2 $$D_{0}\leq h\nu -\Delta IP-KER_{\max}$$

Equation 2 describes the charge transfer image of Figure 3 (top). The C_2_H_2_^+^ fragment, which is assumed to have the lowest energy acetylene cation structure, must be due to dissociation on the dissociative charge transfer (DCT) surface, 2.01 eV above the ground state asymptote. Consequently, an upper limit on the BDE of *D*_0_ ≤ 1.04 ± 0.20 eV (24.0 ± 4.6 kcal/mol) is derived. The image of the Zn^+^ photofragment provides a BDE of *D*_0_ ≤ 2.70 ± 0.20 eV (62.3 ± 4.6 kcal/mol) from eq 1 assuming dissociation in the ground state. This is consistent with the BDE from the C_2_H_2_^+^ fragment, but this upper bound indicates that significant energy is lost in the internal states of the neutral molecular fragment when the Zn^+^ cation fragment is produced. All of these energetics include error bars, indicated with the pink shaded box in the KER spectra, that are derived from the width of the KER distribution of the Ar^+^ fragment from Ar_2_^+^ photodissociation under identical VMI settings.^88^ The gray boxes in these figures show the approximate level of baseline noise outside the edge of these images.

The C_2_H_2_^+^ image in Figure 3 exhibits some anisotropy, such that most of the Zn^+^(C_2_H_2_) ions that photofragment into Zn + C_2_H_2_^+^ are those with their transition moment perpendicular to the laser polarization. The Zn^+^ image is nearly isotropic. This is represented quantitatively by beta parameters derived from the angular intensity distribution: β = −0.35 for the C_2_H_2_^+^ fragment and β = −0.09 for the Zn^+^ fragment (see Figures S3 and S4 in the Supporting Information). The polarization of the excitation laser is lost in the Zn^+^ photofragment, possibly due to a dissociation time frame with the same order of magnitude as the rotational period of the molecule, or excited state mixing. The image of C_2_H_2_^+^ from Zn^+^(C_2_H_2_) has two distinct regions. The inner portion appears to mirror the anisotropy of the outer portion, and extends to roughly 0.6 ± 0.20 eV. The outer region appears to have weaker intensity due to the radius-squared dependence of the energy, but as the KER spectrum shows (top of Figure 3) the two regions/peaks have roughly the same intensity. Note that the image of the Zn^+^ photofragment is smaller in area even though there is greater KER. This is an effect of the greater mass of ^64^Zn than that of C_2_H_2_.

### Zn^+^(C_2_H_4_) Photofragment Images

Figure 4 shows the images of the C_2_H_4_^+^ fragment from photodissociation of Zn^+^(C_2_H_4_) at both 355 and 266 nm and their corresponding KER spectra. This is assumed to be the lowest energy ethylene cation, but we refer to it by its chemical formula because its structure cannot be confirmed. As in the case of the Zn^+^(C_2_H_2_) dissociation, the image is filled with intensity at low KER, indicating a broad distribution of internal energies in the fragment.

**Figure 4:**
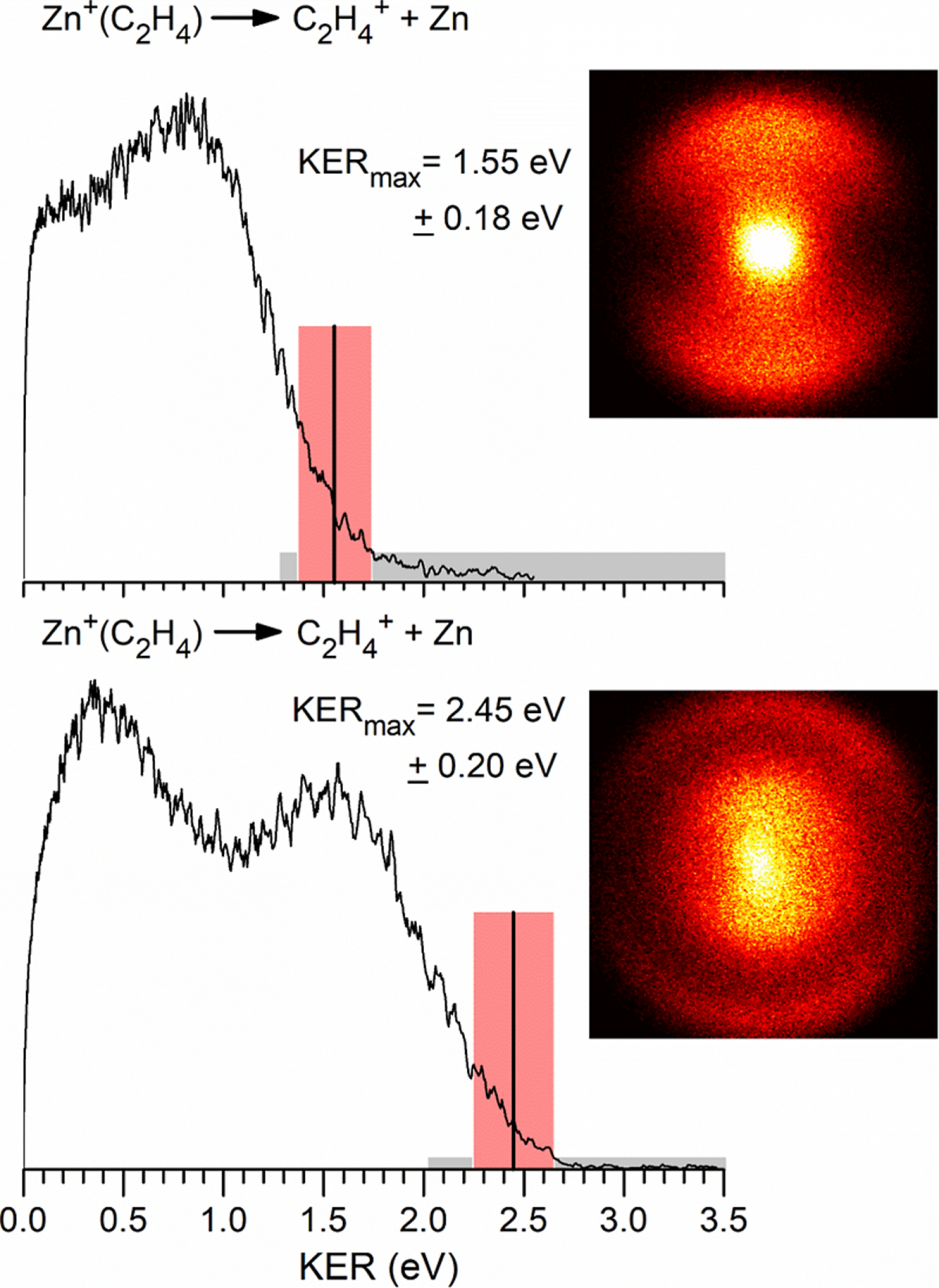
Photofragment images with kinetic energy release spectra of the C_2_H_4_^+^ fragment from Zn^+^(C_2_H_4_) photodissociation at 10 mJ/pulse of vertically polarized 355 nm (top) and 5 mJ/pulse of vertically polarized 266 nm (bottom).

Using eq 2 and ΔIP = IP­(ethylene) – IP­(zinc) = 10.51 eV – 9.39 eV = 1.12 eV, the value of KER_max_ (1.55 ± 0.18 eV) for the image of the C_2_H_4_^+^ fragment channel at 355 nm gives an upper limit on the bond dissociation energy of *D*_0_ ≤ 0.82 ± 0.18 eV (18.9 ± 4.2 kcal/mol) for Zn^+^(C_2_H_4_). Similarly, the image of the C_2_H_4_^+^ fragment channel at 266 nm gives a KER_max_ of 2.45 ± 0.20 eV for an upper limit of *D*_0_ ≤ 1.09 ± 0.20 eV (25.1 ± 4.6 kcal/mol) which is consistent with the BDE from the image at 355 nm. Both upper limits are consistent with the findings of Kleiber and co-workers who reported a BDE of *D*_e_ = 0.86 eV from a Birge–Sponer extrapolation of their vibronic spectra.^54^ The Birge–Sponer extrapolation employs vibrations only in the lower portion of the potential and is expected to be only a rough estimate for the bond energy. Therefore, our BDE value is likely to be closer to the actual value.

Both images in Figure 4 exhibit anisotropy along the vertical axis, implying that complexes that photofragment are those with the Zn^+^-C_2_H_4_ bond parallel to the laser polarization (β = 0.97 for the 355 nm image and β = 0.44 for the 266 nm image, see Figures S5 and S6 in the Supporting Information). This is a marked deviation from the Zn^+^(C_2_H_2_) charge-transfer image which had perpendicular anisotropy. The vertical anisotropy is most pronounced on the outer perimeter of both images, consistent with a model of recoil along the dissociation axis and a parallel transition dipole moment. The bright inner portion of the image of C_2_H_4_^+^ at 355 nm (top of Figure 4) appears to have less anisotropy and its border lies at approximately 0.5 ± 0.18 eV. For the image of the 266 nm C_2_H_4_^+^ fragment, the more isotropic inner portion extends to roughly 1.2 ± 0.20 eV. The change in symmetry of different areas of these images suggests that the inner and outer components correspond to dissociation on different potential energy surfaces.

The images of the Zn^+^ fragments from the photofragmentation of Zn^+^(C_2_H_4_) at 355 and 266 nm in Figure 5 are isotropic (β = 0.11 and β = 0.17 respectively, see Figures S7 and S8 in the Supporting Information) with smaller sizes than their corresponding C_2_H_4_^+^ fragment images. Similar to the Zn^+^ photofragment from Zn^+^(C_2_H_2_), the polarization information on the incident light is lost to either a long dissociation time scale or a transition dipole moment that is a result of state-mixing. Again, the size difference between the Zn^+^ and C_2_H_4_^+^ images is due to the heavier masses of the Zn^+^ fragments, which have lower velocity and thus a smaller radial spread than C_2_H_4_^+^ fragments with the same kinetic energy. The KER spectrum for the 355 nm image leads to a KER_max_ value of 1.29 ± 0.18 eV. Since the ground state potential is the only one with a Zn^+^ fragment accessible at this photon energy, the BDE is derived with eq 1; this gives *D*_0_ ≤ 2.20 ± 0.18 eV (50.7 ± 4.2 kcal/mol). Similarly, the Zn^+^ image at 266 nm with KER_max_ = 2.39 ± 0.20 eV yields a BDE of *D*_0_ ≤ 2.27 ± 0.20 eV (52.3 ± 4.6 kcal/mol). While both of these upper limits are consistent with those determined from the charge transfer images, the higher values are not as useful.

**Figure 5:**
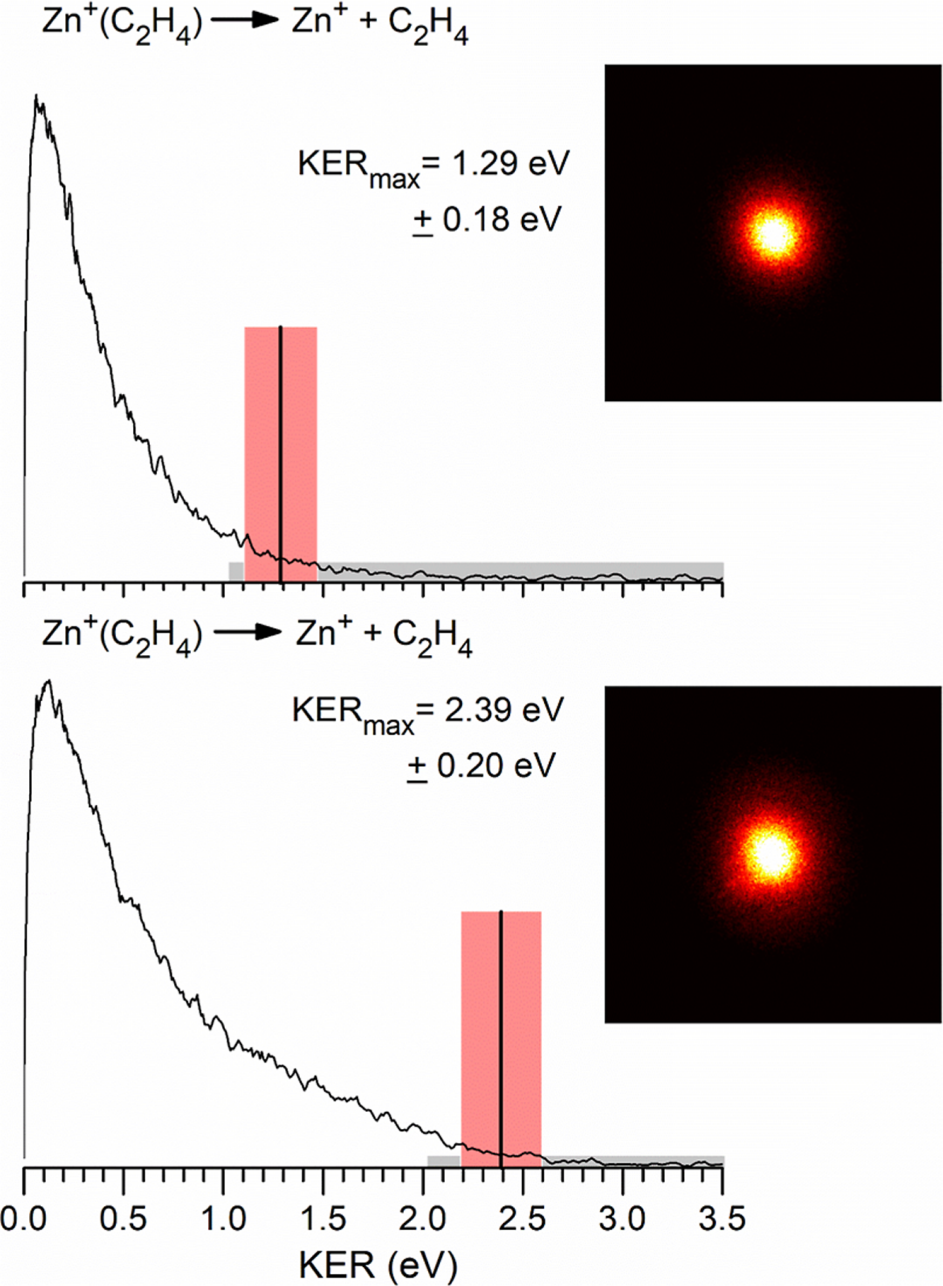
Photofragment images with kinetic energy release spectra of the Zn^+^ fragment from Zn^+^(C_2_H_4_) photodissociation at 10 mJ/pulse of vertically polarized 355 nm (top) and 5 mJ/pulse of vertically polarized 266 nm (bottom).

It is possible to explore the character of the bonding in these zinc complexes by comparing their BDEs with those of other M^+^(acetylene) and M^+^(ethylene) complexes studied previously. Typical values of the BDE for transition metal (TM) ion complexes with acetylene are in the range of 1.6–2.6 eV, placing the Zn^+^-(C_2_H_2_) BDE of 1.04 ± 0.20 eV as the lowest among such complexes studied so far. The BDE of Zn^+^-(C_2_H_4_) at *D*_0_ ≤ 0.82 ± 0.18 eV is also below the range of values (0.9–2.0 eV) for first-row singly charged TM ions bound to ethylene. These dissociation energies are comparable to those of corresponding alkaline earth metal ion complexes (e.g., Mg^+^-(C_2_H_2_): *D*_0_ = 0.76 eV;^59^ Ca^+^-(C_2_H_2_): *D*_0_ = 0.81 eV;^58^ Mg^+^-(C_2_H_4_): *D*_0_ = 0.70 eV).^56^ The transition metal cation–π complexes are understood to have partial covalent character from σ-donation and π back-bonding interactions involving the partially filled d orbitals. It is therefore understandable that Zn^+^, Mg^+^ and Ca^+^ complexes would have much weaker bonding, as there are no partially filled d orbitals available for these interactions, and the bonding is mostly electrostatic. Each of these metal cations has an *n*s^1^ valence electron configuration and their ionic radii are somewhat similar,^98^ suggesting similar electrostatic interactions. Zn^+^ has the filled 3d^10^ subshell which may provide a small amount of back-bonding, but the low bond energies suggest that this is not a major factor.

In addition to these bond energy trends, the amount of charge-transfer interaction in metal acetylene complexes is reflected in the distortion of the linear acetylene structure and in the frequencies of the C–H stretching vibrations. These issues have been investigated extensively in previous work on metal ion-acetylene complexes.^41–53^ Other transition metal complexes have structures in which the acetylene moiety has its hydrogens bent away from the metal ion, providing IR activity to the symmetric C–H stretch vibration. Consistent with the limited amount of charge-transfer, Zn^+^(C_2_H_2_) has a structure in which the acetylene is essentially linear and the symmetric C–H stretch has virtually no intensity.^48^ Likewise, the d back-bonding in most transition metal-acetylene complexes weakens the framework bonding in acetylene and the C–H stretches are shifted significantly to lower frequencies. The antisymmetric C–H stretch of Zn^+^(C_2_H_2_) is observed and somewhat red-shifted, but this shift is the smallest of any transition metal cation–acetylene complex.^48^ These observations are consistent with the weak bonding in this system. Corresponding studies for the infrared spectroscopy of Zn^+^(C_2_H_4_) are not available.

The difference in BDEs between Zn^+^(C_2_H_2_) and Zn^+^(C_2_H_4_) is small but significant. The Zn^+^-(C_2_H_4_) bond (0.82 eV) is weaker than the Zn^+^-(C_2_H_2_) bond (1.04 eV), consistent with the behavior of other singly charged TM ions. This trend is not expected if the bonding is purely electrostatic, because the polarizability of ethylene (42.6 × 10^–25^ cm^3^) is much greater than that of acetylene (33.3 × 10^–25^ cm^3^).^99,100^ The apparent difference is the efficiency of the Zn^+^ π back-donation with the two molecules. Acetylene possesses two orthogonal π* orbitals, whereas ethylene has only one, making acetylene a more efficient π acceptor.^4,5^ The covalent character of the bonding to zinc is small compared to the other transition metals, but it is apparently this small covalent interaction that explains the difference in bonding between acetylene and ethylene.

### Ground State Theory

DFT computations were carried out to investigate the structure and energetics of Zn^+^(C_2_H_2_) and Zn^+^(C_2_H_4_) using the B3LYP, M06, M06-L, and MN15-L functionals coupled with the def2-TZVP basis set. The results of these computations are summarized in Table 1 and the full details are presented in the Supporting Information. All functionals find that the ground state of both complexes are doublets. Quartet structures were investigated for completeness, but were much higher in energy (see Supporting Information). All functionals also find that the C_2v_ structure is the most stable for Zn^+^(C_2_H_2_). However, all functionals but MN15-L find that the most stable structure for Zn^+^(C_2_H_4_) is not one with C_2v_ symmetry, but instead one where the Zn^+^ ion is centered over a carbon of the ethylene (Figure 6). The stationary point determined by constrained calculations identified the C_2v_ structure as a transition state between the two degenerate minima. The barrier between these minima was found to be 0.14–0.57 kcal/mol, depending on the functional (see Figure 6). It is not clear if these minima are chemically relevant or an artifact of the density functional theory, especially considering the small barrier and that ab initio calculations by Kleiber and co-workers settled on the C_2v_ structure.^54^ All functionals significantly overestimate the experimental Zn^+^-(C_2_H_4_) and Zn^+^-(C_2_H_2_) bond dissociation energies, however B3LYP agrees best with the experimental values. Because the BDE for Zn^+^-(C_2_H_4_) has been determined by two independent experiments, theory seems to be in error. While DFT is known to overestimate BDEs for first-row transition metals bound to π-systems, it is nonetheless surprising that functionals designed to handle transition metal systems underperform for these cases, especially since M06 and MN15-L are recommended for determining BDEs.^101–103^

**Table 1:** Computed Energies (Hartrees) for Zn^+^(C_2_H_2_) and Zn^+^(C_2_H_4_) Employing Different DFT Functionals with a def2-TZVP Basis Set.^a^ ^,^ ^b^

	B3LYP	M06	M06 L	MN15-L
acetylene	–77.339399	–77.272199	–77.321417	–77.261266
ethylene	–78.572809	–78.496390	–78.551080	–78.479022
Zn^+^	–1779.106516	–1779.012779	–1778.999551	–1779.044013
Zn^+^(C_2_H_2_)	–1856.486908	–1856.329252	–1856.363057	–1856.349166
Zn^+^(C_2_H_4_)	–1857.726048	–1857.560485	–1857.598108	–1857.571286
Zn^+^(C_2_H_4_) C_2v_	–1857.725820	–1857.560131	–1857.597201	–1857.571319
*D*_0_ Zn^+^-(C_2_H_2_)	25.7 (1.12)	27.8 (1.20)	26.4 (1.15)	27.5 (1.19)
*D*_0_ Zn^+^-(C_2_H_4_)	29.3 (1.27)	32.2 (1.40)	29.8 (1.29)	30.3 (1.31)
*D*_0_ Zn^+^-(C_2_H_4_) C_2v_	29.2 (1.27)	32.0 (1.39)	29.2 (1.27)	30.3 (1.31)
r(C–C) acetylene	1.19682	1.19473	1.19724	1.19681
r(C–C) ethylene	1.32475	1.31898	1.32047	1.33410
r(C–C) Zn^+^(C_2_H_2_)	1.20878	1.20814	1.20960	1.21862
r(C–C) Zn^+^(C_2_H_4_)	1.35616	1.35384	1.35491	1.36362
r(C–C) Zn^+^(C_2_H_4_) C_2v_	1.35208	1.34872	1.34717	1.36185
r(Zn^+^–C) Zn^+^(C_2_H_2_)	2.49316	2.45042	2.44718	2.45125
r(Zn^+^–C) Zn^+^(C_2_H_4_)	2.36636	2.30536	2.26586	2.36998
r(Zn^+^–C) Zn^+^(C_2_H_4_) C_2v_	2.55758	2.51527	2.51174	2.50506

a All neutrals are singlets and all cations are doublets.

b Dissociation energies (*D*_0_) are in kcal/mol with eV values in parentheses. All energies are zero-point corrected. Bond distances are given in Angstroms.

**Figure 6:**
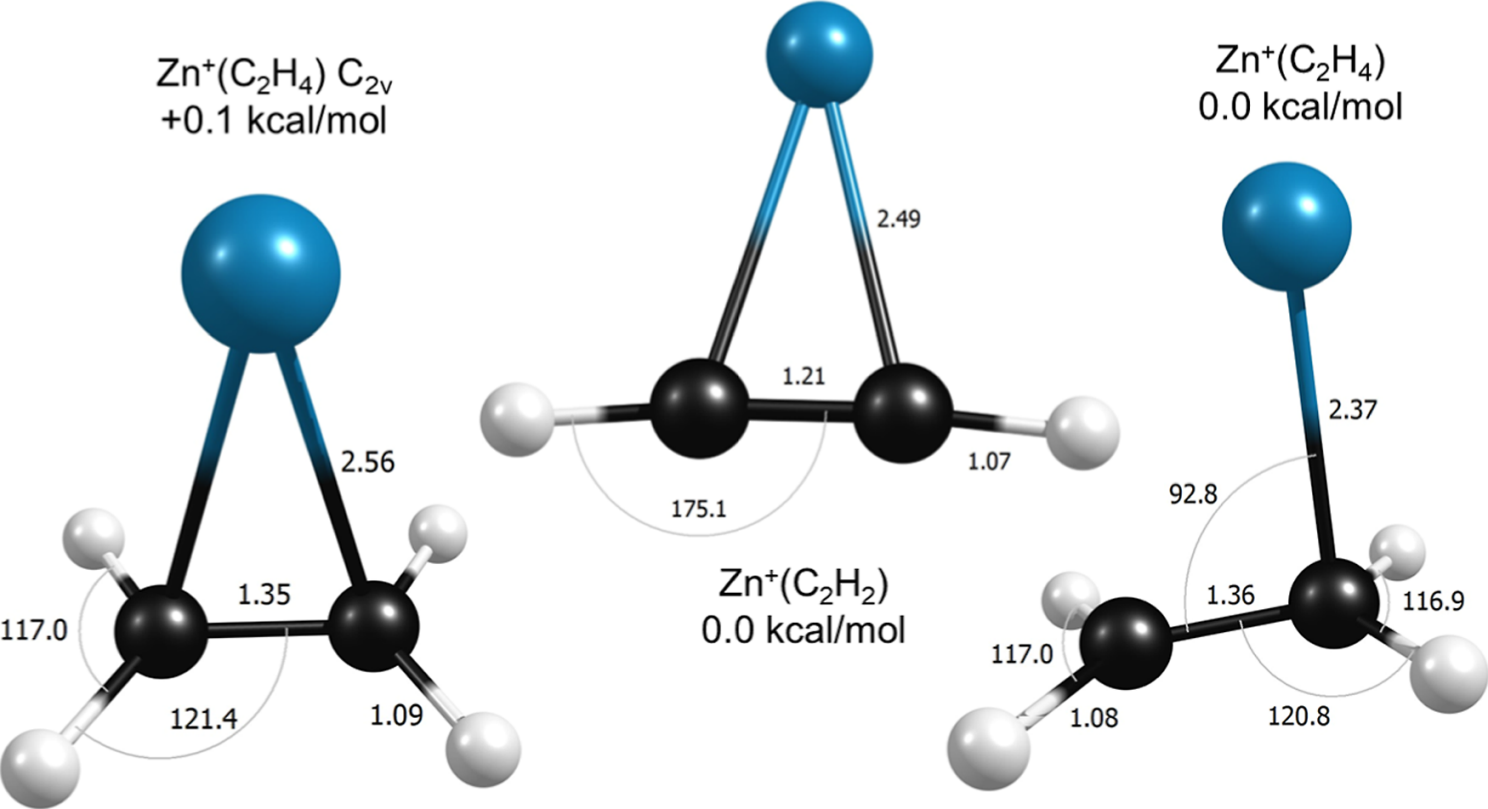
Optimized equilibrium structures for Zn^+^(C_2_H_2_) (middle) and Zn^+^(C_2_H_4_) (right) along with the C_2v_ stationary point for Zn^+^(C_2_H_4_) (left) at the B3LYP/def2-TZVP level.

The equilibrium structure for Zn^+^(C_2_H_2_) predicted by DFT supports our assessment of the Zn^+^(C_2_H_2_) bond. The T-shaped structure with near-linear acetylene (∠CCH 178°) suggests that the bond is more electrostatic with less back-donating character. Furthermore, according to theory, there is minimal elongation of the C–C bond in both Zn^+^(C_2_H_4_) and Zn^+^(C_2_H_2_) compared to free ethylene and acetylene respectively, which is again indicative of negligible back-donation into the π* orbitals. For the acetylene complex, the elongation is predicted to be between 0.012 and 0.022 Å, and for the ethylene complex it is between 0.029 and 0.035 Å in the minimized Zn^+^(C_2_H_4_) structure and between 0.026 and 0.030 Å in the C_2v_ structure. Comparing this to previous theoretical work on other first row transition metal ions bound to ethylene or acetylene, these results are symptomatic of electrostatic bonding. Additionally, previous BDEs tend to be higher for the ethylene complex compared to acetylene when the metal–ligand bond is predominantly electrostatic, consistent with the higher polarizability of ethylene. This is exactly what the DFT reflects here. However, the order of the BDEs is reversed in our experiment, with acetylene more strongly bound than ethylene. This pattern also occurs for the corresponding Fe^+^ and Cr^+^ complexes studied previously.^11–20^ It is unclear if this trend holds for other first-row transition metals, as the Mn^+^, Co^+^, Ni^+^, and Cu^+^ bond energies with acetylene have yet to be measured.

### Excited States of Zn^+^(C_2_H_2_)

The lowest energy excited states of Zn^+^ and acetylene are both greater than 5 eV above the ground state.^104–108^ Therefore, the only accessible states which could yield photodissociation with the photon energies used in this experiment are the charge-transfer state, whose asymptote is at 2.01 eV, and the ground state. Figure 7 schematically represents the potential energy surfaces of the different electronic excited states of Zn^+^(C_2_H_2_), including these lower energy states but extending up to higher energies to include the possibility (discussed below) of two-photon absorption. These surfaces are based on TD-DFT calculations at the B3LYP/def2-QZVP level. The energies of the Zn^+^(C_2_H_2_) complex electronic states were computed as the distance between the Zn^+^ ion and the center of the C≡C bond in acetylene was varied in the C_2v_ symmetry π-complex structure, with all other bond lengths and angles held fixed. The calculated energetic order of these states is unchanged, but each curve is shifted vertically to known asymptotic energies of separated zinc and acetylene fragments from tabulated electronic excited states of Zn and Zn^+^,^104^ as well as the first few excited states of acetylene^105,106^ and the lowest energy excited state of acetylene cation.^107,108^

**Figure 7:**
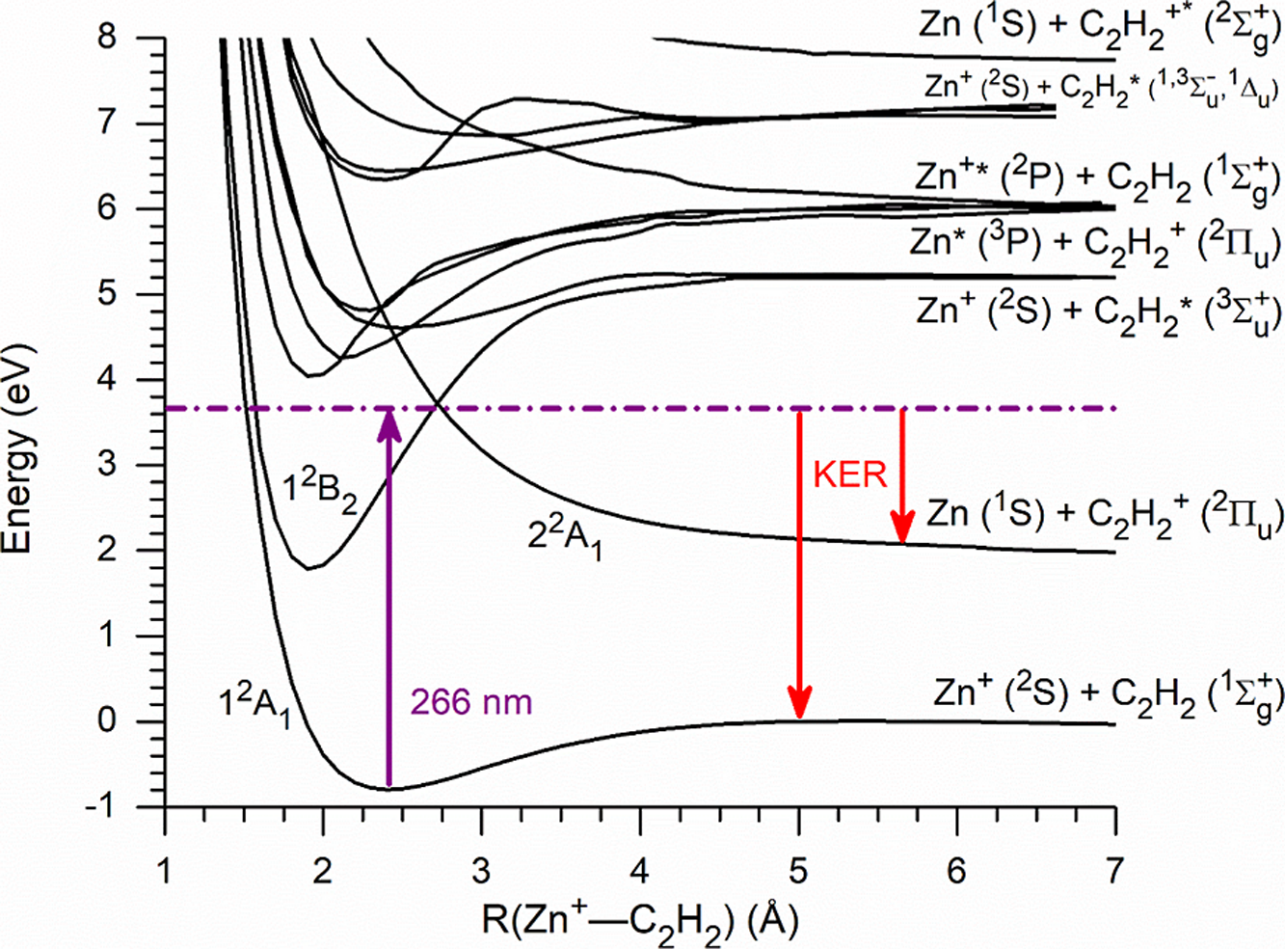
Schematic diagram outlining the low-lying electronic states of Zn^+^(acetylene) and their separated Zn^+^ atom and acetylene molecule asymptotes based on rigid body potential energy surface scans along various distances between Zn^+^ and the center of the C≡C bond in acetylene at the B3LYP/def2-QZVP level of theory.

Our TD-DFT computations show that Zn^+^(C_2_H_2_) exhibits a strongly bound first excited state (1^2^B_2_) that is crossed by a DCT state (2^2^A_1_). The well depth of this first excited state is 3.42 eV, which is likely an overestimation given the excessive binding energy found for the 1^2^B_2_ state of Zn^+^(C_2_H_4_) in the subsequent section. Assuming a ground state BDE of about 1.0 eV, the 1^2^B_2_ excited state appears to be vertically accessible by photons in the 3.2–6.2 eV range, and the DCT 2^2^A_1_ state intersects it at an energy 3.7 eV above the ground state asymptote. Many additional states are found at energies greater than those accessible with one photon at 266 nm. These correlate to various excited states of Zn^+^ and acetylene.

These potential energy surfaces reveal possible mechanisms which account for the structure in the Zn^+^(C_2_H_2_) photofragment images of Figure 3. At the excitation wavelength of 266 nm, it seems that the only excited state accessible is the bound 1^2^B_2_ state. Because this state is strongly bound, and the excitation is well below its dissociation limit, dissociation cannot occur in this state. The 2^2^A_1_ charge transfer state is higher than the expected energy of an absorbed 266 nm photon, so the direct excitation of this state charge transfer state is not possible. Instead, dissociation to produce the charge-transfer acetylene cation fragment must occur through predissociation, via a curve crossing from the 1^2^B_2_ state to the 2^2^A_1_ charge-transfer state. This predissociation route explains the low yield of the C_2_H_2_^+^ fragment in the experiment. It also explains the perpendicular polarization of the image of this fragment. Direct excitation to the 2^2^A_1_ state would be allowed via *z* polarization and would give a parallel-polarized image, which is not observed. However, excitation of the 1^2^B_2_ state is allowed in *y* polarization. Initial excitation of this “doorway” state, followed by crossing to the charge transfer state, produces the perpendicular polarization of the C_2_H_2_^+^ image. Production of the Zn^+^ fragment from photodissociation of Zn^+^(C_2_H_2_) at 266 nm probably occurs through a nonadiabatic relaxation from the 1^2^B_2_ excited state to the ground state surface. This has been suggested to occur in the similar Mg^+^(C_2_H_4_) complexes.^55^ This pathway would produce excess energy in vibrations, yielding low kinetic energy. It would also slow the dissociation, resulting in the isotropic image. The isotropic image with low KER is therefore consistent with this mechanism for production of the Zn^+^ fragment.

One last detail of the C_2_H_2_^+^ image is interesting to consider. The inner portion of the image corresponding to lower kinetic energy extends to roughly 0.6 ± 0.20 eV and is more isotropic in its angular distribution. Low KER can result from vibrational excitation in the C_2_H_2_^+^ fragment that occurs with charge transfer, but the angular distribution of this should be the same as the charge-transfer channel. This suggests that this signal may be coming from yet another dissociation mechanism. A possible explanation is two-photon absorption, which is not uncommon when the first photon accesses a bound state with a strong transition moment (e.g., oscillator strength for the 1^2^A_1_ → 1^2^B_2_ transition is 0.12; see Supporting Information). For example, we found similar two-photon effects in our recent photofragment imaging study of Mg^+^(benzene).^74^ Assuming that the BDE is about 1.0 eV, as derived from the outer edge of the image, two photons at 266 nm would produce excitation that is 8.3 eV above the ground state. This excitation brings several different excited states of zinc cation and acetylene into play. One possibility is dissociation on the charge transfer surface corresponding to the asymptote of ground state zinc atom (^1^S) and the first excited state of the acetylene ion (^2^Σ_g_^+^) at 7.71 eV, which would result in KER_max_ ≤ 0.57 eV. This agrees with the boundary of the inner region of intensity in the image. Two-photon absorption could also affect the Zn^+^ image. This could produce the Zn^+^ (^2^P) + C_2_H_2_ (^1^Σ_g_^+^) asymptote at 6.07 eV, resulting in a KER_max_ ≤ 2.2 eV. Another intriguing possibility is that the neutral C_2_H_2_ fragment isomerizes from acetylene to vinylidene (H_2_C=C) during dissociation. The net change in enthalpy for this process has been determined to be 2.06 ± 0.17 eV.^109^ If such a process occurs, Zn^+^ fragments would also have less energy available to them, with KER up to 1.56 ± 0.37 eV. Acetylene and its cation are both able to undergo other structural rearrangements in their electronic excited states.^105–108^ Acetylene can distort to a *cis*-bent structure with both hydrogens on the same side of the C≡C bond in C_2v_ symmetry or a *trans*-bent structure with both hydrogens displaced on opposite sides of the C≡C bond in C_2h_ symmetry. Again, these processes would produce lower KER values. In summary, two-photon absorption is definitely possible in this system, perhaps inducing several additional dissociation channels. All of these would produce low energy fragments and would not affect our determination of the KER_max_ value used for bond energy determination.

### Excited States of Zn^+^(C_2_H_4_)

Figure 8 shows a schematic representation of the low-energy excited states of Zn^+^(C_2_H_4_) based on the spectroscopy of Kleiber and co-workers^54^ and TD-DFT calculations at the B3LYP/def2-QZVP level. These calculations scanned the potential energy surface of these excited states as the distance between the Zn^+^ ion and the center of the C=C bond was varied in the C_2v_ symmetry π-complex structure, using a rigid body approximation where the bond lengths and angles in ethylene were held constant. The relative order of these calculated potential energy surfaces was retained, but the curves were translated vertically to correspond to the correct asymptotic energy of separated ethylene and zinc fragments, analogous to the surfaces for Zn^+^(C_2_H_2_) in Figure 7. The asymptotic energies correspond to well-known electronic excited states of Zn and Zn^+^,^104^ along with excited states of ethylene and ethylene cation.^110–112^ The 1^2^B_2_ state correlates to ground state Zn^+^ (^2^S) and the first triplet electronic excited state of ethylene (^3^B_1u_), and the 1^2^A_2_ state correlates to ground state Zn (^1^S) and the first excited state of the ethylene cation (^2^B_3g_). Asymptotes corresponding to the ground state Zn atom (^1^S) and the second (^2^A_g_) and third (^2^B_2u_) excited states of ethylene cation are also included. These ethylene cation state energies were derived from vertical transition energies in the spectroscopy of that ion.^111^ The energy of the lowest-lying excited state of ethylene (^3^B_1u_) is not known precisely. Furthermore, this excited state has two conformers through rotation about the C=C bond; the first excited state has a minimum energy of approximately 2.9–3.2 eV in a D_2d_ structure where the CH_2_ moiety has been rotated 90°with respect to the C=C bond axis. The vertical transition from the 0° ground state (^2^A_g_) to the 0° first excited state (^3^B_1u_) was measured to be approximately 4.3 eV^110^—this is the energy represented schematically in Figure 8 for the asymptote of the 1^2^B_2_ excited state complex. The ethylene cation has similar characteristics in its electronic structure, with the energy of the lowest excited state (^3^B_1u_) at the ground state geometry calculated as 3.6 eV (represented in Figure 8 after addition of ΔIP as 4.7 eV). This state in its relaxed conformation (D_2h_ symmetry with a 90° angle between the planes of the two CH_2_ moieties) has a calculated energy relative to the ground state ion of ∼1.8 eV at the CASSCF level.^111^ These calculations place the energy of the second excited state (^2^A_g_) as roughly 4.4 eV and that of the third (^2^B_2u_) at 6.0 eV, which when combined with the ΔIP value form the 5.5 and 7.1 eV asymptotes in Figure 8.

**Figure 8:**
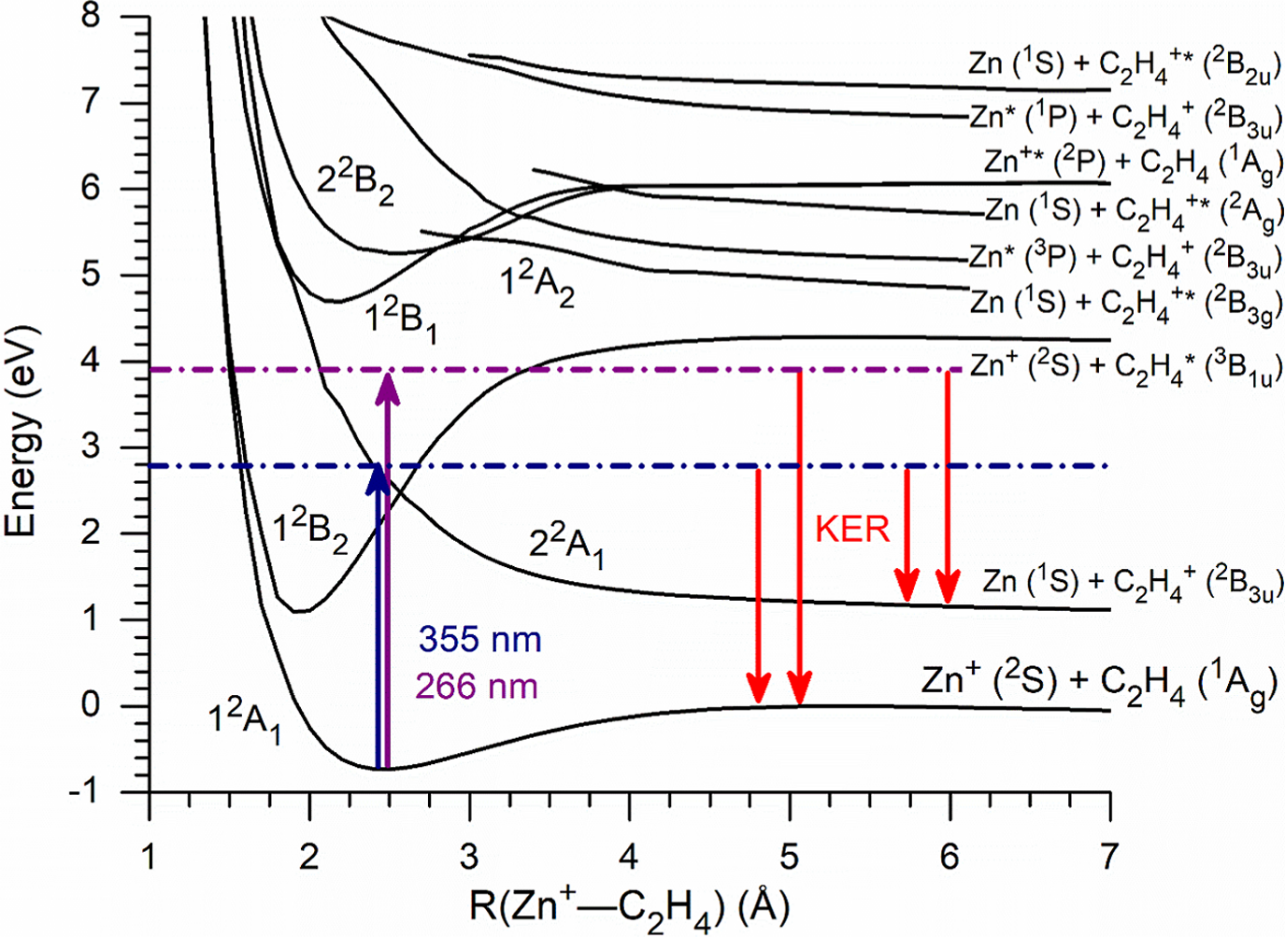
Schematic diagram outlining the low-lying electronic states of Zn^+^(ethylene) and their separated Zn^+^ atom and ethylene molecule asymptotes based on rigid body potential energy surface scans along various distances between Zn^+^ and the center of the C=C bond in ethylene at the B3LYP/def2-QZVP level.

The potential energy curves of Figure 8 mostly reproduce those based on CIS (configuration interaction-singles) calculations by Kleiber and co-workers,^54^ but our calculations find a much deeper well for the first bound excited state (1^2^B_2_) and thus stronger binding; this supports their spectroscopic findings for the 1^2^B_2_ ← 1^2^A_1_ excitation. However, the estimated dissociation energy of the 1^2^B_1_ excited state via their Birge–Sponer extrapolation is 2.76 eV, which is considerably more than the ∼1.4 eV value from our DFT calculations. Also, our calculated ground state well depth of 0.74 eV underestimates the Kleiber experimental value of 0.86 eV,^54^ so these surfaces are best viewed qualitatively. The first excited state (1^2^B_2_) is predicted in our computations to be vertically accessible by photon energies between 2.5–5.2 eV, which could explain the broad continuum centered at 450 nm that has been reported by Kleiber for the 1^2^B_2_ ← 1^2^A_1_ transition.^54^ The bound 1^2^B_2_ state is calculated to cross the repulsive 2^2^A_1_ charge-transfer state at an energy of 2.45 eV above the ground state asymptote. A vertical transition to this state is predicted to occur at a photon energy of approximately 3.46 eV. Therefore, either a direct transition or a curve crossing to this state could explain the observed charge transfer channel at 355 nm (3.49 eV). The assignment of 266 nm excitation differs significantly between our excited state calculations and the experiments of Kleiber.^54^ According to our computed energetics, absorption of a 266 nm photon (4.66 eV) could excite either higher vibrational levels of the bound 1^2^B_2_ state or the 2^2^A_1_ charge transfer state. However, the Kleiber experiments and their theory clearly show that the 1^2^B_1_ excited state lies at lower energy than we predict. Therefore 266 nm excitation likely lands in either the upper vibrational levels of this 1^2^B_1_ bound state or the 2^2^A_1_ charge transfer state. Fragmentation to produce Zn^+^ at both photodissociation wavelengths is most likely nonadiabatic and due to curve crossing to the ground state potential; this has already been suggested based upon comparable behavior of the Mg^+^(C_2_H_4_) complex.^55,56^

Combining the information from the potential energy surfaces of Figure 8 with the Zn^+^(C_2_H_4_) photofragment images of Figure 4, photodissociation measured in the C_2_H_4_^+^ fragment channel after absorption of a single photon is expected to occur on the lowest-lying DCT surface (2^2^A_1_). This makes sense energetically for the outer anisotropic portions of each image, using the previously known bond energy of 0.86 eV. The polarization of the images at both 355 and 266 nm is parallel to the laser polarization, which is expected for the 1^2^A_1_ → 2^2^A_1_ transition. Therefore, the main signal producing the highest KER signal at both wavelengths is coming from direct excitation of the charge transfer excited state.

It remains to consider the possible source(s) of the lower KER signal in these images. The bright inner portion of the image of C_2_H_4_^+^ at 355 nm (top of Figure 4) appears to have less anisotropy and its border lies at approximately 0.5 ± 0.18 eV. If an already excited Zn^+^(C_2_H_4_) absorbs a second 355 nm photon in a resonance-enhanced two-photon dissociation process, it may land in the 2^2^B_2_ or higher vibrational levels of the 1^2^B_1_ state (whose wells coincide in Figure 8). Following this, a curve crossing could occur into the second DCT state which correlates to the separated ground state zinc atom and the excited ethylene ion: Zn (^1^S) and C_2_H_4_^+^* (^3^B_3g_) at 5.5 eV. If this were the case, two 355 nm photons would land at 6.12 eV above the ground state asymptote, so the total excess energy would be 0.62 eV, which is possible given this is only slightly more than the KER_max_ of the inner circle of at least 0.5 ± 0.18 eV. The inner portion of the 266 nm C_2_H_4_^+^ photofragment image that extends to roughly 1.2 ± 0.20 eV could result from the absorption of two 266 nm photons that would place the complex 8.46 eV above the ground state asymptote, and dissociation on the DCT surface correlating to the Zn (^1^S) and C_2_H_4_^+^* (^2^B_2u_) asymptote at 7.1 eV would lead to a KER_max_ ≤ 1.33 eV. For both photodissociation wavelengths, the additional possibility of dissociation on other DCT potentials between these and 2^2^A_1_ with concomitant rovibrational excitation of C_2_H_4_^+^ cannot be eliminated. Another conceivable explanation is that the C_2_H_4_^+^ photofragment may initially be ethylene, but which isomerizes to structures such as ethylidene (H_3_C–CH) or a bridged intermediate. Calculations at the SA4-CASPT2­(11/7) level reported a bridged minima structure with one of the hydrogens above the C=C bond that is 1.02 eV less stable than the ground state ethylene ion.^111^ If the C_2_H_4_^+^ photofragment was detected in this configuration, then the maximum possible KER would be 1.66 eV. Finally, a less convoluted explanation for both images is that the C_2_H_4_^+^ ions contributing to the inner section of the image are just those from the 2^2^A_1_ state with a large amount of excess energy redistributed into their vibrational modes.

Analyzing the Zn^+^ photofragment images of Zn^+^(C_2_H_4_) (Figure 5) in an analogous way, we note an interesting characteristic of these images: the difference in KER_max_ (∼1.10 eV) between them is approximately the difference in photon energies (4.66 eV – 3.49 eV = 1.17 eV). This may be coincidental, or perhaps these fragment ions are produced on surfaces which correlate to the same asymptote so that any additional photon energy is converted directly to excess translational energy. From Figure 8, it is not clear how this would be achieved. Kleiber and co-workers have assigned absorption at 266 nm to be a part of the 1^2^A_1_ → 1^2^B_1_ transition and subsequent dissociation to occur on the ground state surface.^54^ This is reasonable since they find the 1^2^B_1_ state to be much more strongly bound than our TD-DFT calculations predict. They do not report any photodissociation at 355 nm, but based on their assignment, absorption at this photon energy would not access the 1^2^B_1_ manifold. Regardless, since both of these images are expected to be from fragments on the ground state 1^2^A_1_ potential, it must be that the same amount of excess energy is lost to internal energy of the molecular fragments.

## Conclusions

The Zn^+^(C_2_H_2_) and Zn^+^(C_2_H_4_) complexes were generated in the gas phase via laser vaporization and studied with selected-ion photofragment imaging. Photofragment images of both the charge-transfer fragment channel and the Zn^+^ fragment channel provided upper limits on the bond dissociation energies, which characterized the magnitude of the cation–π interaction in these complexes. The dissociation energies obtained are *D*_0_ ≤ 0.82 ± 0.18 eV (18.9 ± 4.2 kcal/mol) for Zn^+^-(C_2_H_4_) and *D*_0_ ≤ 1.04 ± 0.20 eV (24.0 ± 4.6 kcal/mol) for Zn^+^-(C_2_H_2_). The first of these substantiates the spectroscopic findings by Kleiber and co-workers of *D*_e_ = 0.86 eV for Zn^+^-(C_2_H_4_), and provides evidence that the upper limit is an accurate measurement of the true BDE. DFT studies employing the B3LYP, M06, M06-L, and MN15-L functionals agree on the most stable structures for the Zn^+^(C_2_H_2_) and Zn^+^(C_2_H_4_) complexes, revealing an unexpected deviation from the typical C_2v_ structure for Zn^+^(C_2_H_4_). All functionals overestimated the BDE of both the acetylene and ethylene complexes, with B3LYP performing best.

The photofragment images of Zn^+^(C_2_H_2_) and Zn^+^(C_2_H_4_) have markedly different angular distributions, with the main structure for Zn^+^(C_2_H_2_) having a polarization perpendicular to the laser excitation and that for Zn^+^(C_2_H_4_) having a parallel polarization. Electronic structure calculations with TD-DFT make it possible to explain this behavior. The charge-transfer state in Zn^+^(C_2_H_2_) lies at higher energies beyond those accessible with the available UV wavelengths employed here. Excitation accesses a bound state, which leads to dissociation via a curve crossing to the charge-transfer state. The ground-to-bound state transition moment determines the perpendicular polarization of the resulting photofragment image. In Zn^+^(C_2_H_4_), the charge-transfer state lies at lower energy and it is accessed directly at both 355 and 266 nm, producing the expected parallel-polarization for the images. Images for both complexes have internal structure corresponding to lower kinetic energies, in some cases with polarization different from that at higher KER values. The pattern of excited states suggests that this signal may come from two-photon absorption processes that lead to dissociation out of higher energy excited states.

Analysis of the bonding in these complexes revealed a primarily electrostatic interaction between Zn^+^ and each of the two ligands, but with a non-negligible covalent character that is enhanced in the acetylene complex. This mirrors the behavior of other late first-row transition metal cations with ethylene and acetylene. Further experimental studies of late first-row transition metal ions bound to acetylene would provide useful insight for a more complete evaluation of bonding trends. The Zn^+^(C_2_H_2_) and Zn^+^(C_2_H_4_) complexes illustrate the intermediate behavior of zinc between that of a transition metal and an alkaline earth metal.

## Supplementary Material



## Notes

Further details of the DFT calculations, including structures and energetics for all ions considered, as well as additional images and information on angular distributions and the employed fitting routine (PDF)
