# Supplementary material for: Photodissociation Dynamics of the Zn+(Acetylene) and Zn+(Ethylene) Cation‑π Complexes
Source: J Phys Chem A. 2026 Jun 30;130(28):5484–98. doi: 10.1021/acs.jpca.6c02947 (PMC13383836; doi:10.1021/acs.jpca.6c02947)
Supplement: Supplementary file 1 [file jp6c02947_si_001.pdf]

Supporting Information

Photodissociation Dynamics of the Zn^{+}(Acetylene) and Zn^{+}(Ethylene) Cation-π Complexes

John R. C. Blais,[1] Brandon M. Rittgers,[1] Michael A. Duncan[1]*

[1]Department of Chemistry, University of Georgia, Athens, Georgia 30602, United States

*Email: maduncan@uga.edu

Table of Contents

Non-symmetrized $Zn^+(C_2H_2)$ Images……S3

Photofragment Angular Distributions……S5

Angular Distributions for $Zn^+(C_2H_2)$ Images……S5

Angular Distributions for $Zn^+(C_2H_4)$ Images……S7

DFT Computations……S11

Zn……S11

Zn^+……S24

Acetylene……S36

$Acetylene^+$……S52

Ethylene……S68

$Ethylene^+$……S84

$Zn^+(C_2H_2)$ Isomers……S100

B3LYP……S100

M06……S116

M06-L……S132

MN15-L……S148

$Zn^+(C_2H_4)$ Isomers……S164

B3LYP……S164

M06……S184

M06-L……S204

MN15-L……S224

$Zn^+(C_2H_4)$ (Symmetry Constrained C_{2v})……S244

Non-symmetrized $Zn^+(C_2H_2)$ Images

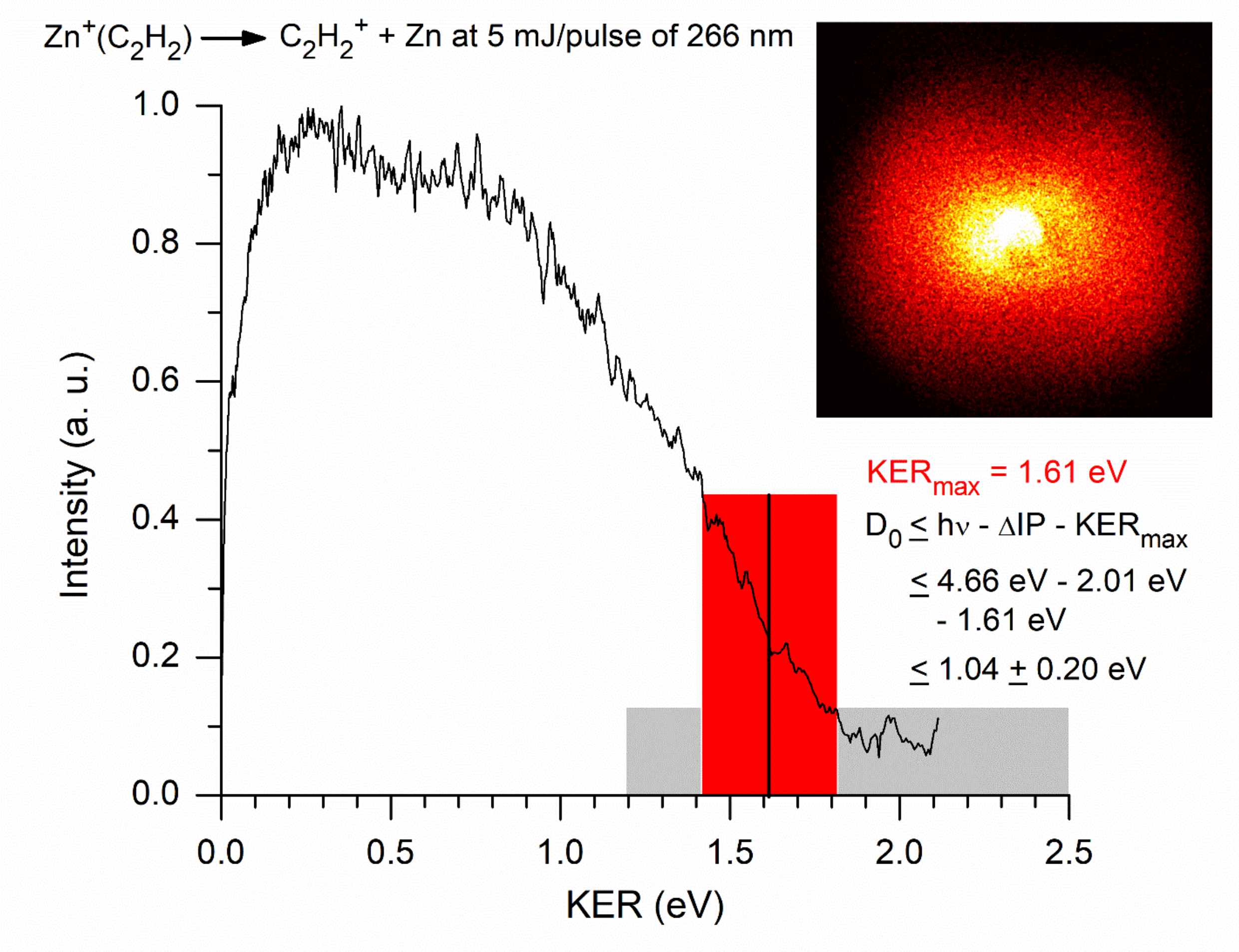

Figure S1. Non-symmetrized photofragment image and kinetic energy release spectrum of the $C_2H_2^+$ fragment from $Zn^+(C_2H_2)$ photodissociation at 5 mJ/pulse of vertically polarized 266 nm. Region with less intensity is due to MCP “dead spot.”

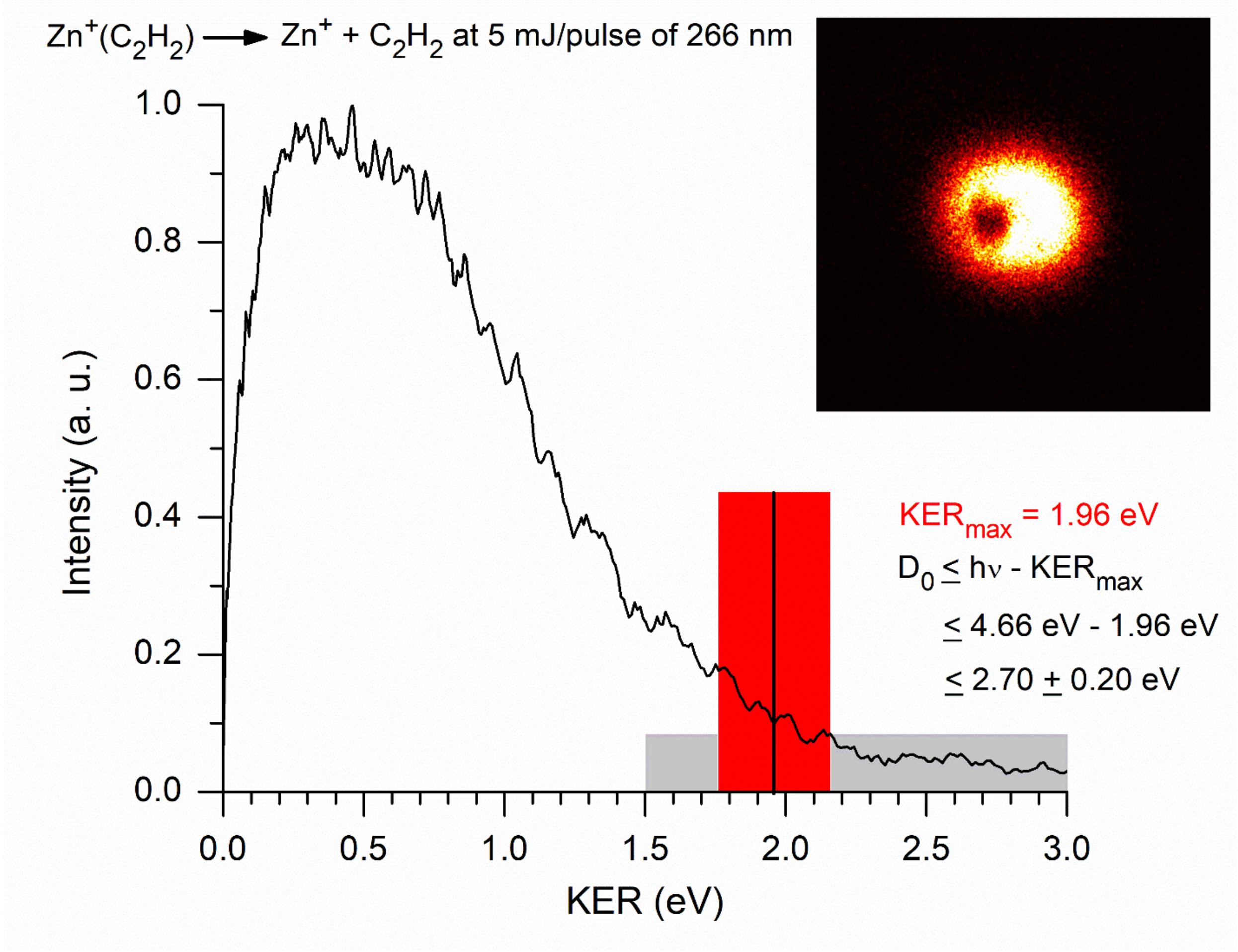

Figure S2. Non-symmetrized photofragment image and kinetic energy release spectrum of the Zn^+ fragment from $Zn^+(C_2H_2)$ photodissociation at 5 mJ/pulse of vertically polarized 266 nm. Region with less intensity is due to MCP “dead spot.”

Photofragment Angular Distributions

The following equation is used for fitting angular distributions:

$$I = \frac{A}{4\pi}\left\{1 + \frac{B}{2}\left[3\,cos^2\left(\frac{\theta\pi}{180} - C\right) - 1\right]\right\}$$

I is the signal intensity. The *A* parameter allows for variation of the amplitude, *B* is the *β* parameter, and *C* is a phase shift parameter correcting for rotation of the image.

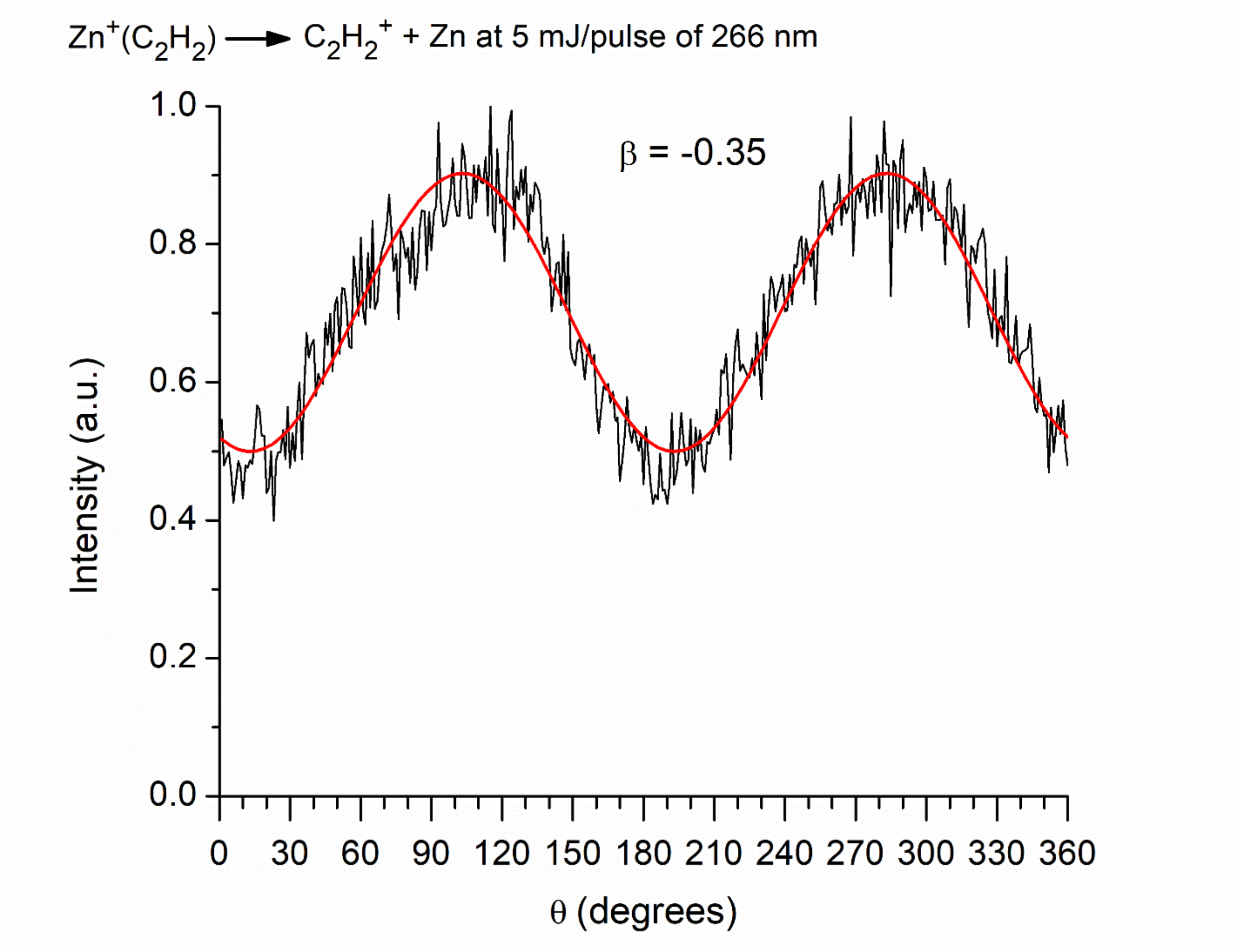

Figure S3. The angular distribution of the sliced $C_2H_2^+$ photofragment image from the dissociation of $Zn^+(C_2H_2)$ with vertically polarized light at 266 nm. The red line is a fit with ß = -0.35.

A = 9.65671 ± 0.03660

B = -0.34996 ± 0.00602

C = 0.22878 ± 0.00924

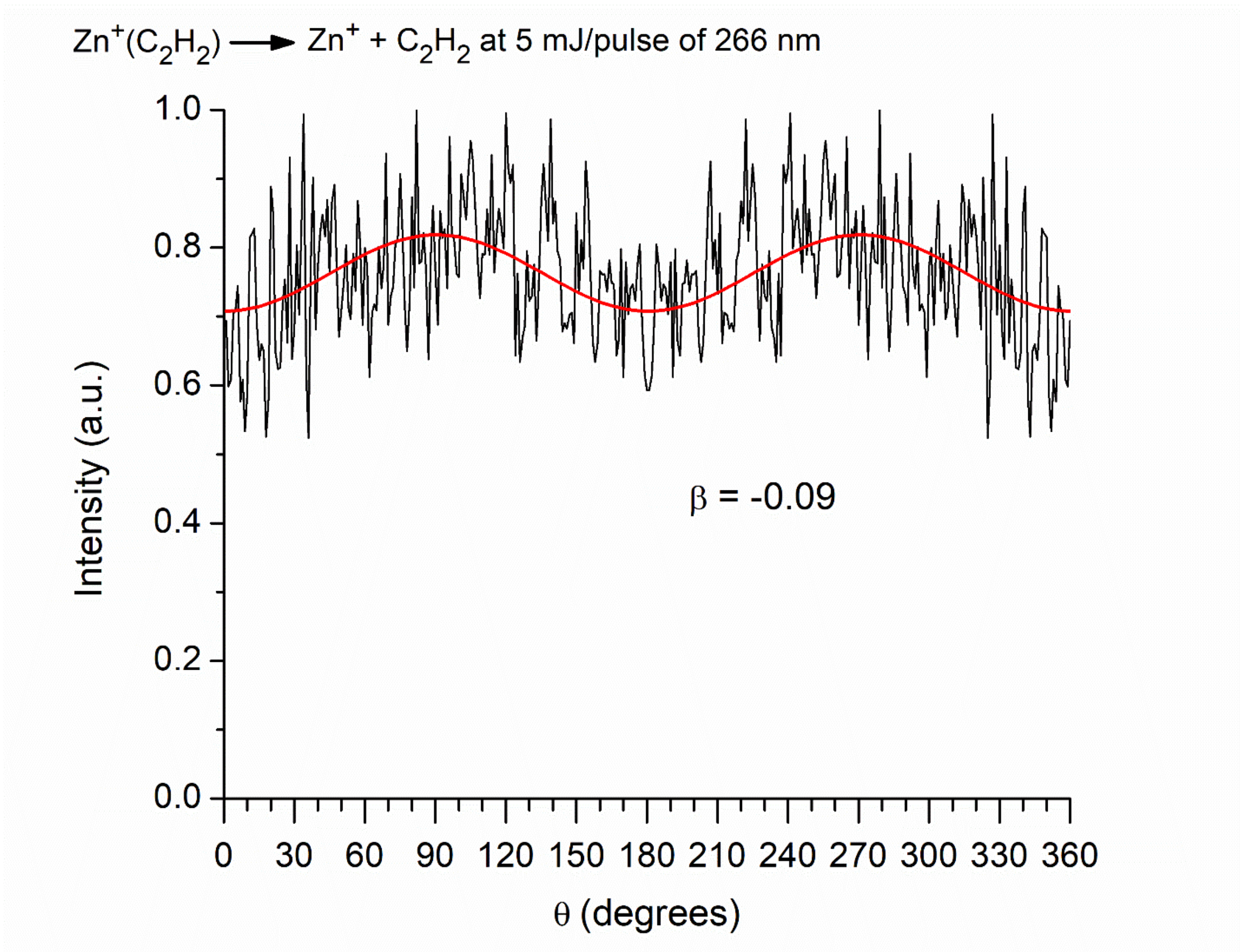

Figure S4. The angular distribution of the sliced Zn^+ photofragment image from the dissociation of $Zn^+(C_2H_2)$ with vertically polarized light at 266 nm. The red line is a fit with ß = -0.09.

A = 9.82352 ± 0.06650

B = -0.09461 ± 0.01129

C = 0.00873 ± 0.06102

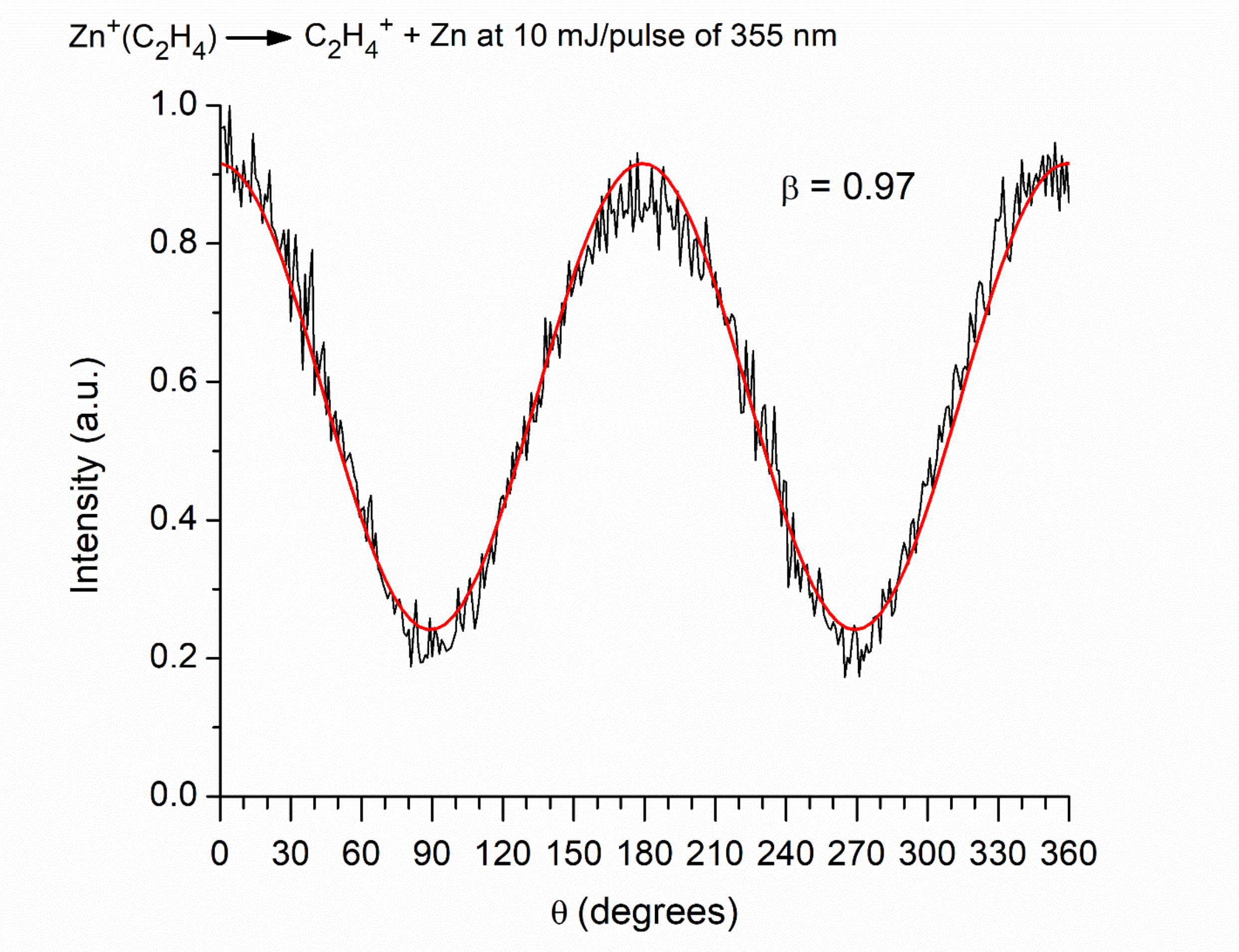

Figure S5. The angular distribution of the sliced $C_2H_4^+$ photofragment image from the dissociation of $Zn^+(C_2H_4)$ with vertically polarized light at 355 nm. The red line is a fit with ß = 0.97.

A = 5.85824 ± 0.03011

B = 0.96546 ± 0.01177

C = -0.01373 ± 0.00454

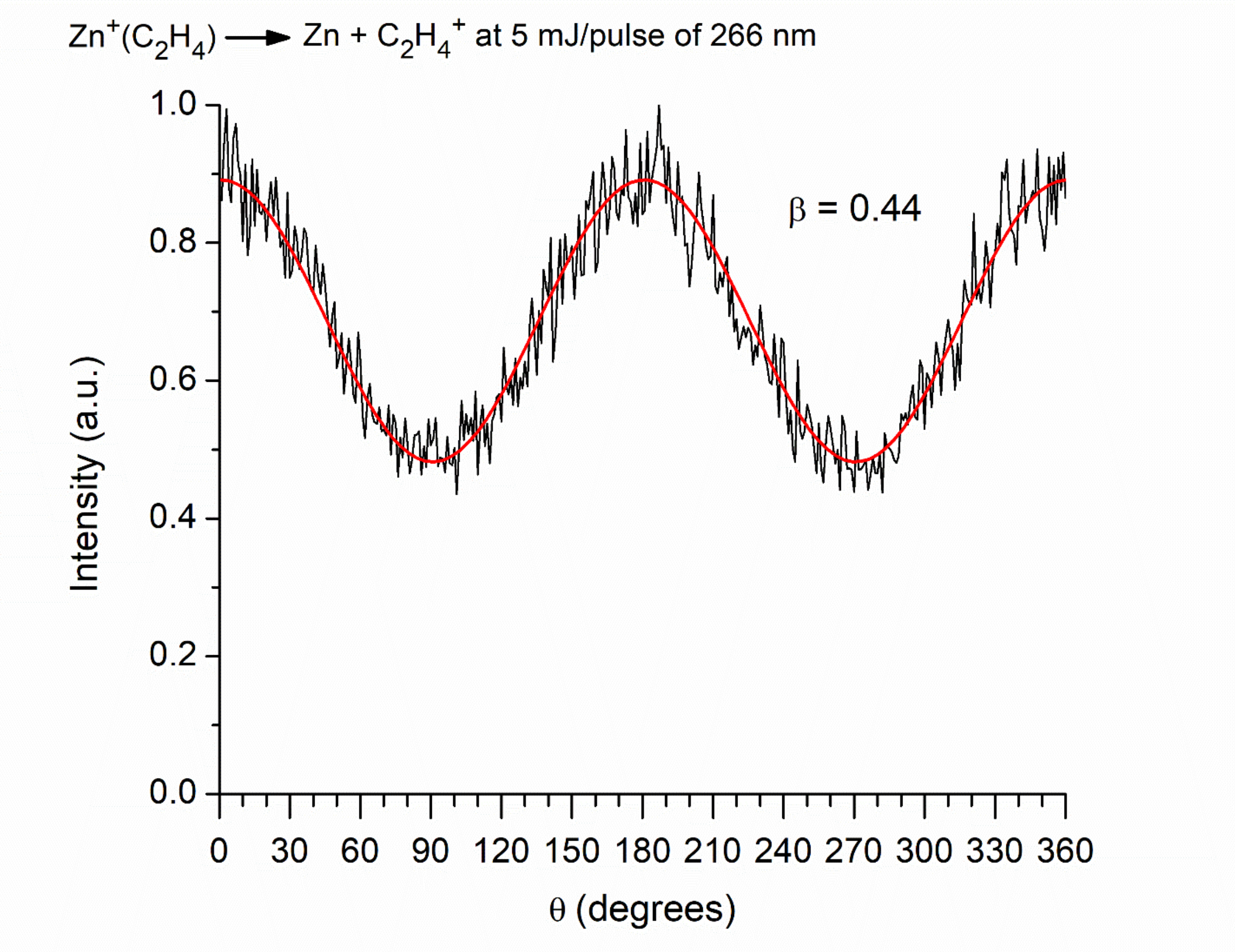

Figure S6. The angular distribution of the sliced $C_2H_4^+$ photofragment image from the dissociation of $Zn^+(C_2H_4)$ with vertically polarized light at 266 nm. The red line is a fit with ß = 0.44.

A = 7.77291 ± 0.03089

B = 0.44130 ± 0.00769

C = 0.01319 ± 0.00768

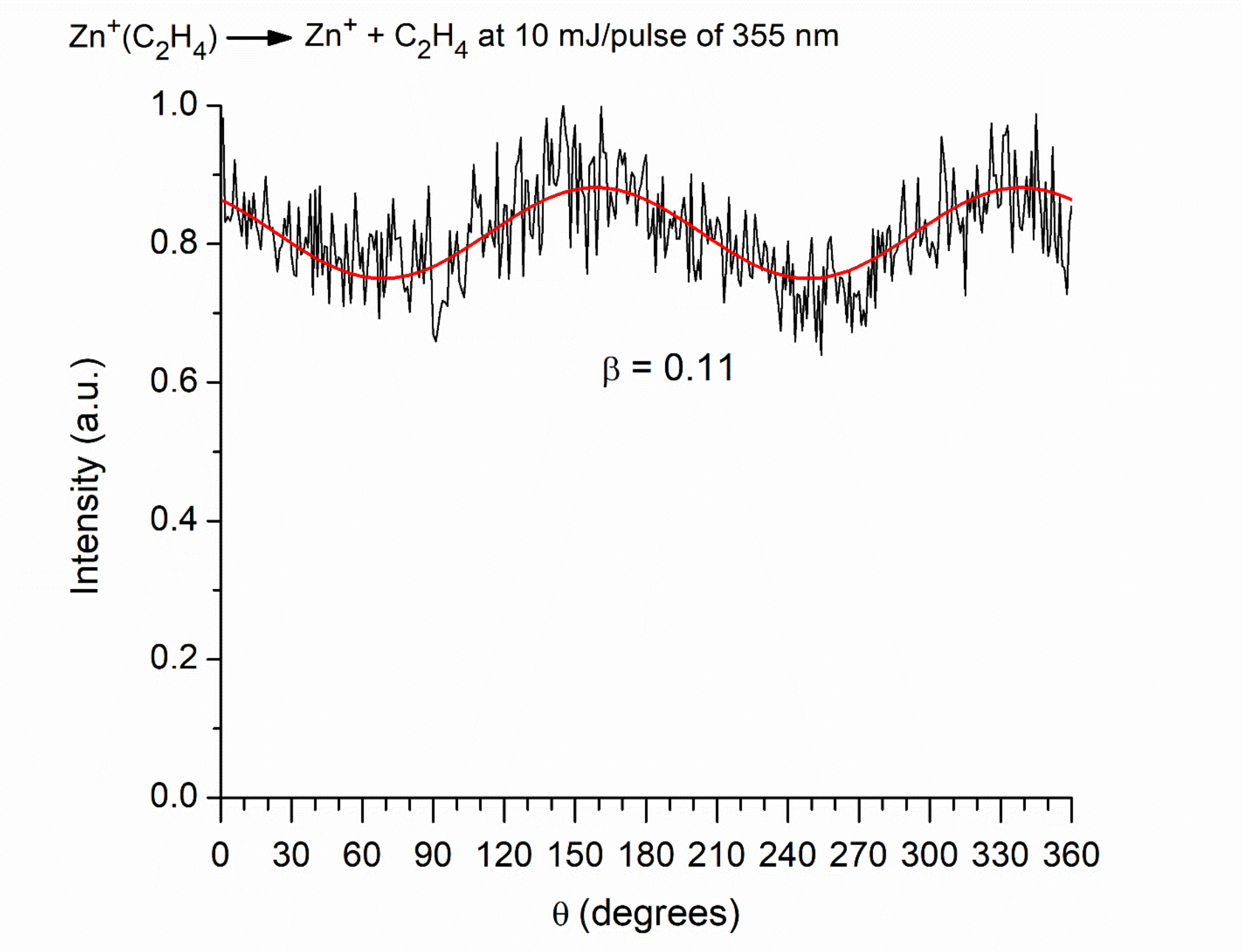

Figure S7. The angular distribution of the sliced Zn^+ photofragment image from the dissociation of $Zn^+(C_2H_4)$ with vertically polarized light at 355 nm. The red line is a fit with ß = 0.11.

A = 9.97731 ± 0.03900

B = 0.11030 ± 0.00686

C = -0.37407 ± 0.03022

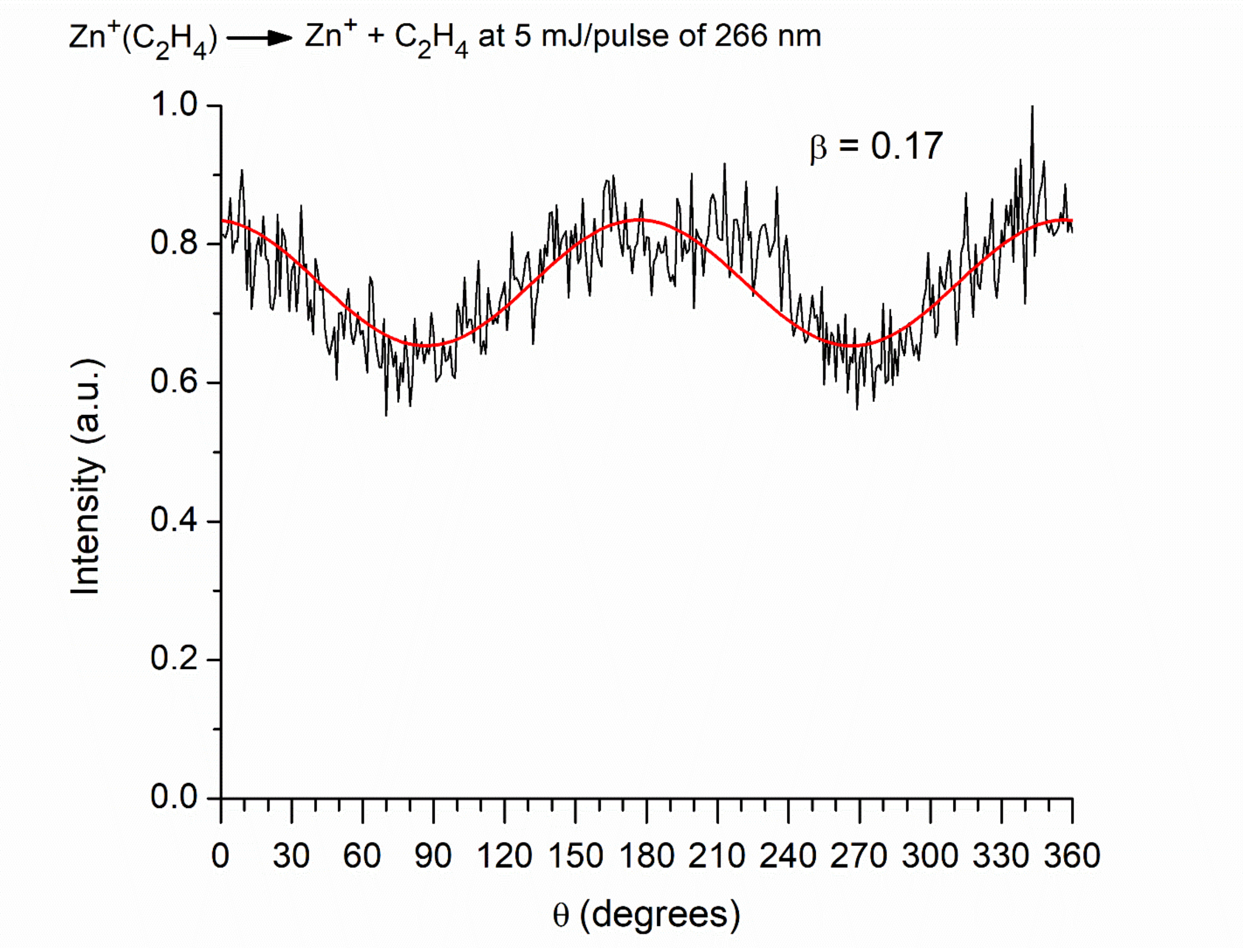

Figure S8. The angular distribution of the sliced Zn^+ photofragment image from the dissociation of $Zn^+(C_2H_4)$ with vertically polarized light at 266 nm. The red line is a fit with ß = 0.17.

A = 8.97285 $\pm$ 0.03785

B = 0.16979 $\pm$ 0.00753

C = -0.05780 $\pm$ 0.02119

DFT Computations

All calculations were carried out using an "ultrafine" integration grid, and the optimization threshold for energy and structure optimizations were set to "tight." The "stable=opt" keyword paired with "nosymm" was used on all structures to check for electronic wavefunction stability. All electronic energies are corrected for zero-point vibrational energy. Vibrational frequencies were calculated at the def2/TZVP level. The first 100 electronic transitions were calculated using TD-DFT at the def2/QZVP level.

Zn
B3LYP
m = 1

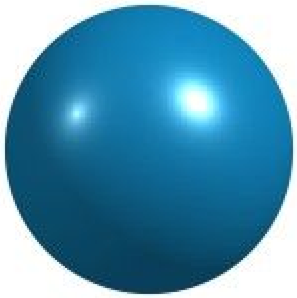

Coordinates:

30	0.000000000	0.000000000	0.000000000

Zero-Point Corrected Electronic Energy:
-1779.452807 Hartrees

Electronic Transitions:

Wavelength (nm)	Oscillator Strength
438.52	0
318.32	0
318.32	0
318.32	0
225.53	0.4758
225.53	0.4758
225.53	0.4758
180.79	0
167.27	0
152.36	0
152.36	0
152.36	0
140.28	0.0358

140.28	0.0358
140.28	0.0358
133.17	0
133.17	0
133.17	0
133.17	0
133.17	0
129.8	0
129.8	0
129.8	0
129.8	0
129.8	0
120.56	0
120.56	0
120.56	0
120.04	0
120.04	0
120.04	0
120.04	0
120.04	0
120.04	0
120.04	0
118.35	0
118.35	0
118.35	0
118.35	0
118.35	0
118.35	0
118.35	0
117.86	0
117.86	0
117.86	0
117.86	0
117.86	0
117.86	0
117.86	0
117.86	0
117.86	0
117.86	0
112.88	0.1595
112.88	0.1595
112.88	0.1595
94.85	0

94.85	0
94.85	0
94.85	0
94.85	0
94.09	0
94.09	0
94.09	0
94.09	0
94.09	0
86.18	0
86.18	0
86.18	0
85.99	0
85.99	0
85.99	0
85.99	0
85.99	0
85.99	0
85.99	0
85.45	0
85.45	0
85.45	0
85.45	0
85.45	0
85.45	0
85.45	0
85.33	0
85.33	0
85.33	0
85.33	0
85.33	0
85.33	0
85.33	0
85.33	0
85.33	0
85.33	0
83.8	0.0692
83.8	0.0692
83.8	0.0692
79.28	0
78.69	0
78.69	0
78.69	0

78.69	0
78.69	0

Zn
M06
m = 1

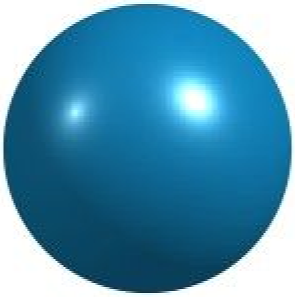

Coordinates:
30 0.000000000 0.000000000 0.000000000

Zero-Point Corrected Electronic Energy:
-1779.367414 Hartrees

Electronic Transitions:

Wavelength (nm)	Oscillator Strength
218.4	0.3885
218.4	0.3885
218.4	0.3885
165.05	0
138.52	0.0594
138.52	0.0594
138.52	0.0594
137.08	0
137.08	0
137.08	0
137.08	0
137.08	0
117.15	0
117.15	0
117.15	0
117.15	0
117.15	0
115.02	0
115.02	0
115.02	0
115.02	0
115.02	0

115.02	0
115.02	0
110.77	0.1734
110.77	0.1734
110.77	0.1734
97.22	0
97.22	0
97.22	0
97.22	0
97.22	0
91.74	0
91.74	0
91.74	0
91.74	0
91.74	0
90.96	0
90.96	0
90.96	0
90.96	0
90.96	0
90.96	0
90.96	0
89.3	0.0949
89.3	0.0949
89.3	0.0949
78.93	0
78.93	0
78.93	0
78.7	0
78.7	0
78.7	0
78.7	0
78.7	0
78.7	0
78.7	0
78.29	0
78.29	0
78.29	0
78.29	0
78.29	0
78.29	0
78.29	0
78.29	0

78.29	0
78.2	0
78.2	0
78.2	0
78.2	0
78.2	0
74.86	0
68.55	0
62.05	0.0002
62.05	0.0002
62.05	0.0002
54.71	0
54.71	0
54.71	0
54.71	0
54.71	0
52.48	0
52.48	0
52.48	0
52.48	0
52.48	0
48.7	0
48.7	0
48.7	0
48.7	0
48.7	0
47.98	0
47.98	0
47.98	0
47.98	0
47.98	0
47.98	0
47.98	0
46.67	0.0751
46.67	0.0751

Zn
M06-L
m = 1

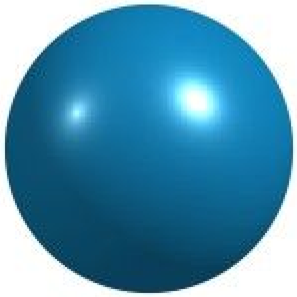

Coordinates:

30 0.000000000 0.000000000 0.000000000

Zero-Point Corrected Electronic Energy:
-1779.338514 Hartrees

Electronic Transitions:

Wavelength (nm)	Oscillator Strength
218.66	0.488
218.66	0.488
218.66	0.488
158.2	0
139.51	0
139.51	0
139.51	0
139.51	0
139.51	0
134.35	0.0169
134.35	0.0169
134.35	0.0169
126.09	0
126.09	0
126.09	0
126.09	0
126.09	0
126.09	0
126.09	0
125.14	0
125.14	0
125.14	0

125.14	0
125.14	0
118.91	0.1795
118.91	0.1795
118.91	0.1795
98.23	0
98.23	0
98.23	0
98.23	0
98.23	0
93.62	0
93.62	0
93.62	0
93.62	0
93.62	0
93.62	0
93.62	0
93.36	0
93.36	0
93.36	0
93.36	0
93.36	0
91.6	0.0703
91.6	0.0703
91.6	0.0703
81.06	0
81.06	0
81.06	0
81.06	0
81.06	0
81.06	0
81.06	0
81.06	0
81.06	0
80.62	0
80.62	0
80.62	0
80.62	0
80.62	0
80.62	0
80.62	0
80.62	0
80.62	0

80.62	0
80.21	0
80.21	0
80.21	0
80.21	0
80.21	0
75.35	0
65.11	0
59.59	0.0115
59.59	0.0115
59.59	0.0115
55.58	0
55.58	0
55.58	0
55.58	0
55.58	0
54.43	0
54.43	0
54.43	0
54.43	0
54.43	0
47.92	0
47.92	0
47.92	0
47.92	0
47.92	0
47.92	0
47.92	0
47.7	0
47.7	0
47.7	0
47.7	0
47.7	0
46.25	0.0821
46.25	0.0821

Zn
MN15-L
m = 1

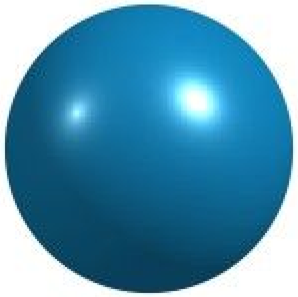

Coordinates:

30	0.000000000	0.000000000	0.000000000

Zero-Point Corrected Electronic Energy:
-1779.380659 Hartrees

Electronic Transitions:

Wavelength (nm)	Oscillator Strength
227.88	0.4888
227.88	0.4889
227.88	0.4887
172.39	0
136.26	0.0351
136.26	0.0351
136.26	0.0351
132.99	0
132.99	0
132.99	0
132.99	0
132.99	0
126.88	0
126.88	0
126.88	0
126.88	0
126.88	0
126.88	0
126.88	0
125.46	0
125.46	0
125.46	0

125.46	0
125.46	0
119.9	0.1883
119.9	0.1883
119.9	0.1883
99.09	0
99.09	0
99.09	0
99.09	0
99.09	0
91.21	0
91.21	0
91.21	0
91.21	0
91.21	0
91.21	0
91.21	0
90.58	0
90.58	0
90.58	0
90.58	0
90.58	0
88.9	0.0863
88.9	0.0863
88.9	0.0863
81.76	0
81.76	0
81.76	0
81.76	0
81.76	0
81.76	0
81.76	0
81.76	0
81.76	0
81.55	0
81.55	0
81.55	0
81.55	0
81.55	0
81.55	0
81.55	0
81.55	0
81.55	0

81.55	0
81.12	0
81.12	0
81.12	0
81.12	0
81.12	0
76.45	0
63.08	0
57.08	0.0184
57.08	0.0184
57.08	0.0184
52.48	0
52.48	0
52.48	0
52.48	0
52.48	0
51.89	0
51.89	0
51.89	0
51.89	0
51.89	0
46.55	0
46.55	0
46.55	0
46.55	0
46.55	0
46.55	0
46.55	0
45.92	0
45.92	0
45.92	0
45.92	0
45.92	0
45	0.0823
45	0.0823

Zn^+
B3LYP
m = 2

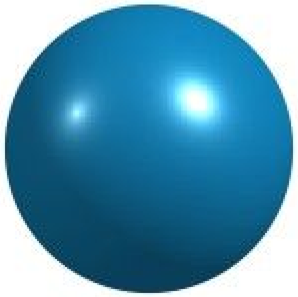

Coordinates:
30 0.000000000 0.000000000 0.000000000

Zero-Point Corrected Electronic Energy:
-1779.106516 Hartrees

Electronic Transitions:

Wavelength (nm)	Oscillator Strength
195.57	0.2604
195.57	0.2604
195.57	0.2604
166.95	0
166.95	0
166.95	0
166.95	0
166.95	0
114.54	0
101.88	0
101.88	0
101.88	0
101.88	0
101.88	0
101.57	0.0019
101.57	0.0019
101.57	0.0019
97.67	0
97.67	0
97.67	0
97.67	0
97.67	0

97.67	0
97.67	0
97.05	0.014
97.05	0.014
97.05	0.014
96.1	0
96.1	0
96.1	0
96.1	0
96.1	0
92.35	0
92.35	0
92.35	0
92.35	0
92.35	0
92.35	0
92.35	0
91.22	0
91.22	0
91.22	0
91.22	0
91.22	0
88.78	0.1427
88.78	0.1427
88.78	0.1427
68.4	0
68.4	0
68.4	0
68.4	0
68.4	0
67.98	0
67.98	0
67.98	0
67.98	0
67.98	0
64.13	0
63.16	0
63.16	0
63.16	0
63.13	0
63.11	0
63.11	0
63.11	0

63.11	0
63.11	0
63.11	0
63.11	0
62.92	0
62.92	0
62.92	0
62.92	0
62.92	0
62.84	0
62.84	0
62.84	0
62.84	0
62.84	0
62.84	0
62.84	0
62.78	0
62.78	0
62.78	0
62.78	0
62.78	0
62.78	0
62.78	0
62.78	0
62.78	0
62.7	0
62.7	0
62.7	0
62.7	0
62.7	0
62.56	0
62.56	0
62.56	0
62.56	0
62.56	0

Zn^{+}
M06
m = 2

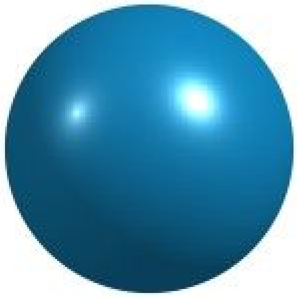

Coordinates:

30	0.000000000	0.000000000	0.000000000

Zero-Point Corrected Electronic Energy:
-1779.012779 Hartrees

Electronic Transitions:

Wavelength (nm)	Oscillator Strength
185.61	0.242
185.61	0.242
185.61	0.242
157.21	0
157.21	0
157.21	0
157.21	0
157.21	0
121.03	0
107.58	0.0001
107.58	0.0001
107.58	0.0001
105.83	0
105.83	0
105.83	0
105.83	0
105.83	0
94.8	0
94.8	0
94.8	0
94.8	0
94.8	0

93.57	0.0268
93.57	0.0268
93.57	0.0268
93.21	0
93.21	0
93.21	0
93.21	0
93.21	0
93.21	0
93.21	0
87.05	0
87.05	0
87.05	0
87.05	0
87.05	0
87.05	0
87.05	0
85.09	0
85.09	0
85.09	0
85.09	0
85.09	0
84.93	0.1392
84.93	0.1392
84.93	0.1392
73.64	0
73.64	0
73.64	0
73.64	0
73.64	0
67.43	0
67.43	0
67.43	0
67.43	0
67.43	0
67.15	0
67.15	0
67.15	0
67.15	0
67.15	0
67.15	0
67.15	0
67.06	0.0194

67.06	0.0194
67.06	0.0194
66.9	0
66.24	0
66.24	0
66.24	0
66.24	0
66.24	0
65.5	0
65.5	0
65.5	0
65.34	0
65.34	0
65.34	0
65.34	0
65.34	0
65.34	0
65.34	0
65.25	0
65.25	0
65.25	0
65.25	0
65.25	0
65.16	0
65.16	0
65.16	0
65.16	0
65.16	0
65.16	0
65.16	0
65.16	0
65.16	0
64.68	0
61.56	0
61.56	0

Zn^{+}
M06-L
m = 2

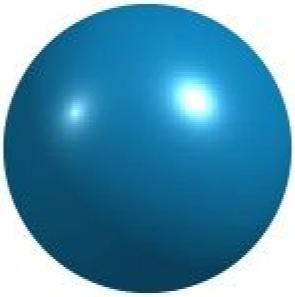

Coordinates:

30	0.000000000	0.000000000	0.000000000

Zero-Point Corrected Electronic Energy:
-1778.999551 Hartrees

Electronic Transitions:

Wavelength (nm)	Oscillator Strength
200.11	0.2722
200.11	0.2722
200.11	0.2722
170.33	0
170.33	0
170.33	0
170.33	0
170.33	0
114.97	0
106.11	0.0002
106.11	0.0002
106.11	0.0002
102.88	0
102.88	0
102.88	0
102.88	0
102.88	0
100.36	0
100.36	0
100.36	0
100.36	0
100.36	0

100.36	0
100.36	0
99.19	0.0144
99.19	0.0144
99.19	0.0144
98.12	0
98.12	0
98.12	0
98.12	0
98.12	0
94.29	0
94.29	0
94.29	0
94.29	0
94.29	0
94.29	0
94.29	0
90.86	0
90.86	0
90.86	0
90.86	0
90.86	0
90.48	0.1408
90.48	0.1408
90.48	0.1408
76.18	0
76.18	0
76.18	0
76.18	0
76.18	0
71.2	0
71.2	0
71.2	0
71.2	0
71.2	0
71.2	0
71.2	0
70.97	0
70.97	0
70.97	0
70.97	0
70.97	0
70.87	0.0154

70.87	0.0154
70.87	0.0154
68.27	0
65.04	0
64.89	0
64.89	0
64.89	0
64.89	0
64.89	0
64.89	0
64.89	0
64.89	0
64.89	0
64.57	0
64.57	0
64.57	0
64.57	0
64.57	0
64.45	0
64.45	0
64.45	0
64.45	0
64.45	0
64.45	0
64.45	0
64.45	0
64.45	0
64.45	0
61.02	0.0057
61.02	0.0057
61.02	0.0057
60.45	0
60.45	0
60.45	0
60.45	0

Zn^{+}
MN15-L
m = 2

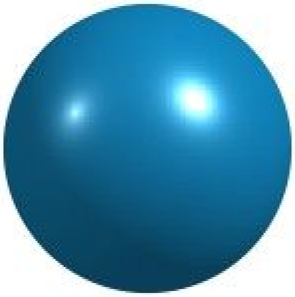

Coordinates:

30	0.000000000	0.000000000	0.000000000

Zero-Point Corrected Electronic Energy:
-1779.044013 Hartrees

Electronic Transitions:

Wavelength (nm)	Oscillator Strength
223.73	0.3066
223.73	0.3066
223.73	0.3066
158.69	0
158.69	0
158.69	0
158.69	0
158.69	0
121.33	0
107.59	0.0001
107.59	0.0001
107.59	0.0001
106.2	0
106.2	0
106.2	0
106.2	0
106.2	0
102.25	0
102.25	0
102.25	0
102.25	0
102.25	0

102.25	0
102.25	0
100.22	0.0219
100.22	0.0219
100.22	0.0219
98.52	0
98.52	0
98.52	0
98.52	0
98.52	0
93.62	0
93.62	0
93.62	0
93.62	0
93.62	0
93.62	0
93.62	0
90.51	0.107
90.51	0.107
90.51	0.107
89.62	0
89.62	0
89.62	0
89.62	0
89.62	0
75.08	0
75.08	0
75.08	0
75.08	0
75.08	0
73.07	0
73.07	0
73.07	0
73.07	0
73.07	0
69.22	0
69.22	0
69.22	0
69.22	0
69.22	0
69.22	0
69.22	0
68.93	0.0098

68.93	0.0098
68.93	0.0098
68.37	0
68.37	0
68.37	0
68.37	0
68.37	0
67.9	0
67.9	0
67.9	0
67.9	0
67.9	0
67.9	0
67.9	0
67.47	0
67.47	0
67.47	0
67.47	0
67.47	0
67.36	0.0302
67.36	0.0302
67.36	0.0302
66.3	0
65.39	0
65.39	0
65.39	0
65.39	0
65.39	0
65.39	0
65.39	0
65.39	0
65.39	0
65.18	0
65.18	0
65.18	0

Acetylene
B3LYP
m = 1

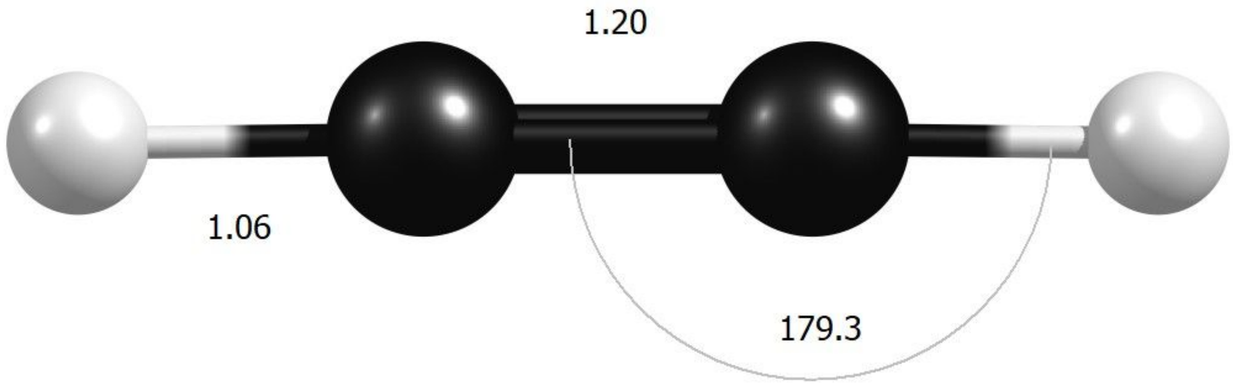

Coordinates:

6	-0.598409000	0.001828000	0.000000000
6	0.598409000	0.001831000	0.000000000
1	-1.661039000	-0.010974000	0.000000000
1	1.661038000	-0.010981000	0.000000000

Zero-Point Corrected Electronic Energy:
-77.339399 Hartrees

Vibrational Frequencies:

Frequency (cm^{-1})	Intensity (km/mol)
619.8052	0
619.8612	0.0712
764.1448	107.9749
2072.5557	0.0039
3417.9487	90.6222
3516.0923	0.0146

Electronic Transitions:

Wavelength (nm)	Oscillator Strength
242.97	0
214.33	0
214.33	0
183.91	0
183.9	0
179.38	0

179.38	0
154.88	0
154.88	0
148.86	0
148.86	0
148.79	0
148.79	0
146.5	0.0704
146.5	0.0704
130.77	0
130.77	0
126.71	0.698
124.69	0.1365
124.69	0.1365
123.89	0
122.42	0
122.42	0
120.41	0
120.41	0
119.53	0
119.53	0
112.03	0
112.03	0
109.55	0
109.3	0
109.3	0.0002
105.2	0
105.18	0
101.39	0
99.63	0
99.63	0
98.44	0.0003
98.43	0
97.23	0
97.23	0
96.88	0
96.88	0
94.98	0
92.61	0
91.49	0
91.49	0
89.44	0
89.44	0

89.3	0.5371
86.71	0
86.4	0.0005
86.36	0
85.14	0
84.66	0.0897
82.21	0.1155
82.2	0.1155
79.49	0
79.11	0
78.41	0.0001
78.39	0
78.39	0
77.7	0.0354
77.7	0.0354
77.2	0
76.02	0
75.77	0.0264
72.97	0
72.97	0
72.67	0.0014
72.67	0.0013
72.26	0
72.26	0
71.93	0
71.63	0
71.63	0
71.18	0.0268
71.18	0.0267
71.18	0.0734
70.87	0.2955
70.66	0
70.65	0
70.45	0
70.44	0
69.8	0.0005
69.8	0
69.34	0
69.34	0
69.23	0
69.22	0
68.61	0
68.61	0.0001

68.05	0
67.91	0
67.9	0
67.42	0.0006
67.42	0
66.68	0
66.68	0
65.38	0.0001

Acetylene
M06
m = 1

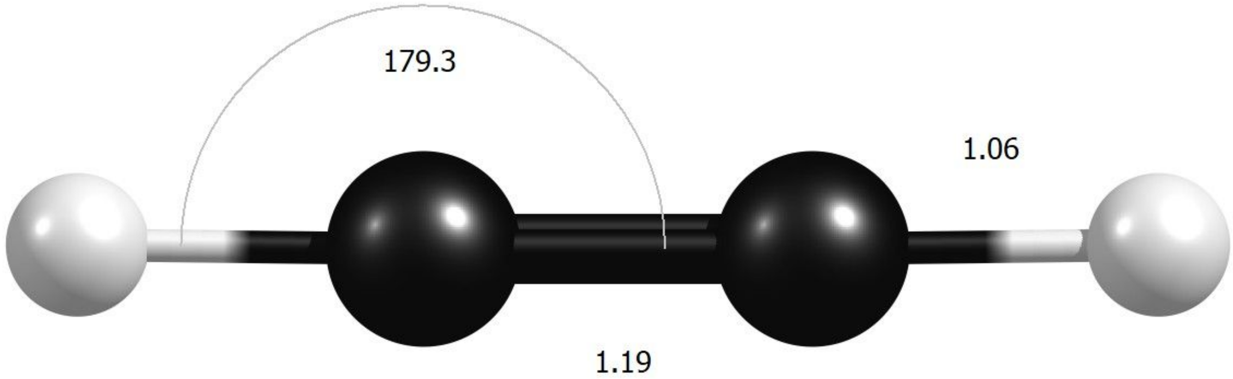

Coordinates:

6	-0.597365000	-0.001801000	0.000001000
6	0.597365000	-0.001793000	-0.000001000
1	-1.661697000	0.010792000	-0.000003000
1	1.661697000	0.010775000	0.000003000

Zero-Point Corrected Electronic Energy:
-77.272199 Hartrees

Vibrational Frequencies:

Frequency (cm^{-1})	Intensity (km/mol)
670.647	0.0001
670.7426	0.0604
786.4374	94.4502
2076.4813	0.0037
3397.5351	76.8363
3500.3222	0.0139

Electronic Transitions:

Wavelength (nm)	Oscillator Strength
189.27	0
189.27	0
188.25	0
161.96	0
161.95	0
156.42	0.0367
156.42	0.0367
127.99	0.6091

124.78	0.1537
124.78	0.1539
124.07	0.0002
121.88	0
121.88	0
117.02	0
117.01	0.0005
111.9	0
105.52	0
102.93	0
102.93	0
101.7	0.0004
101.69	0
94.89	0.4127
94.67	0.009
94.66	0
91.06	0
87.35	0.1388
84.19	0.127
84.18	0.127
81.64	0.0001
80.74	0.0459
80.74	0.0459
79.58	0.1248
78.16	0
77.54	0.0135
77.54	0.0135
76.55	0.0094
76.55	0.0092
76.21	0.1602
73.21	0.0003
73.21	0
71.73	0
71.73	0.0024
71.67	0.113
70.94	0.0002
70.94	0
70.13	0
70.13	0
69.66	0
65.78	0.486
65.77	0.4865
65.18	0.6141

64.49	0.0004
64.44	0.0002
64.44	0.0003
64.2	0.001
64.2	0
62.67	0.2918
62.63	0.3828
62.57	0.0898
62.41	0.0296
62.03	0.0004
59.59	0.0006
57.93	0.001
55.91	0.0001
55.48	0.0096
55.48	0.0096
55	0.0044
54.34	0.0133
54.26	0.1542
54.25	0.1422
53.91	0.001
53.91	0.001
53.66	0.0101
53.44	0.0537
52.63	0
52.63	0
52.23	0
52.22	0.0001
51.78	0.0795
51.16	0
51.12	0
51.1	0.0001
51.02	0
51.01	0
50.91	0
49.97	0.1243
49.79	0
49.79	0
49.7	0.0006
49.37	0
49.37	0
49.24	0.7764
49.23	0.7731
48.8	0

48.8	0
48.53	0.0022
46.11	0
46.1	0.0003
45.84	0.0004
44.95	0

Acetylene
M06-L
m = 1

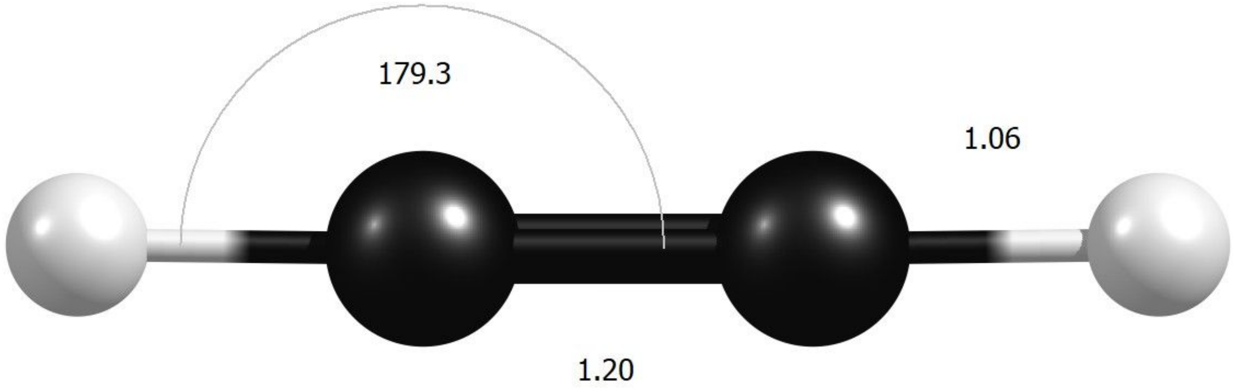

Coordinates:

6	-0.598622000	-0.001793000	0.000004000
6	0.598622000	-0.001770000	-0.000004000
1	-1.660750000	0.010713000	-0.000009000
1	1.660751000	0.010663000	0.000009000

Zero-Point Corrected Electronic Energy:
-77.321417 Hartrees

Vibrational Frequencies:

Frequency (cm^{-1})	Intensity (km/mol)
632.9152	0.0585
633.2672	0.0013
793.9003	94.7882
2069.0583	0.0038
3425.3068	78.5478
3526.7383	0.0153

Electronic Transitions:

Wavelength (nm)	Oscillator Strength
175.01	0
175.01	0
165.52	0
143.63	0
143.62	0
137.03	0.0539
137.03	0.0539

121.2	0.6735
117.38	0.1627
117.38	0.1627
111.4	0
111.4	0
107.94	0
107.5	0
107.5	0.0003
105.07	0
97.84	0.0006
97.83	0
93.48	0
93.48	0
93.27	0
89.93	0.4582
88.88	0.0001
88.86	0
85.78	0
82.18	0.1575
81.03	0.1556
81.02	0.1557
77.93	0.0001
75.73	0.0572
75.72	0.0572
75.27	0.2082
74.35	0
73.86	0.0028
73.85	0.0029
71.7	0.1231
70.39	0.0126
70.38	0.0129
69.23	0
69.23	0
68.64	0.1377
68.61	0
68.61	0
67.31	0.0012
67.31	0
66.09	0
66.09	0
65.93	0
63.3	0.5668
62.86	0.0007

62.86	0.0006
62.07	0.5793
62.05	0.6143
61.96	0.0007
61.95	0
61.72	0.0357
60.57	0.0017
59.53	0.2698
59.52	0.2732
59.31	0.0419
58.74	0.0005
58.15	0.0016
54.84	0.0015
53.72	0.0003
52.65	0.0237
52.53	0.0162
52.52	0.0149
52.34	0.0006
52.17	0
52.17	0
51.77	0.0931
51.77	0.0938
51.19	0.0972
51.13	0
51.13	0
50.64	0.0305
50.02	0.0393
49.64	0
49.62	0.001
49.16	0
49.16	0
48.61	0
48.57	0.0019
48.41	0
48.39	0.0951
48.22	0.9075
48.2	0.9034
47.83	0
47.83	0
47.56	0.0006
47.37	0
47.37	0
47.35	0.0007

46.84	0.0149
46.26	0
46.26	0
44.9	0
44.89	0.0005
43.97	0.0002
42.71	0

Acetylene
MN15-L
m = 1

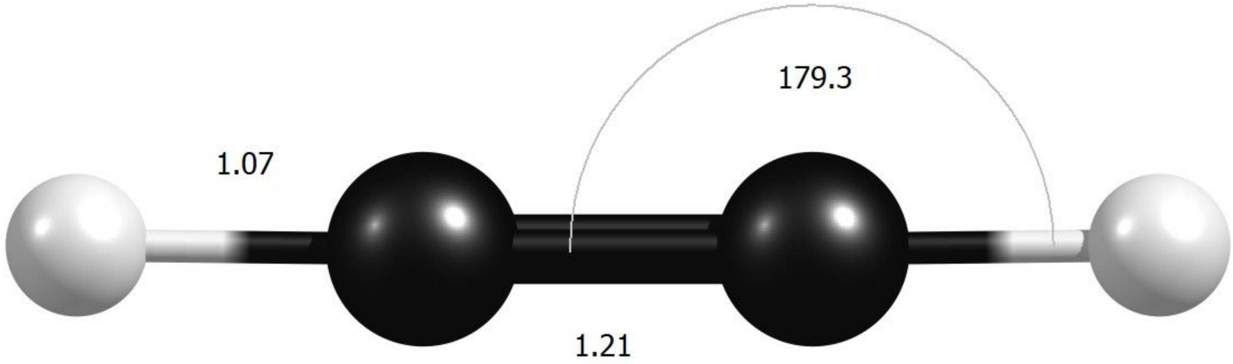

Coordinates:

6	-0.602744000	-0.001770000	0.000003000
6	0.602744000	-0.001760000	-0.000003000
1	-1.674654000	0.010602000	-0.000006000
1	1.674654000	0.010579000	0.000006000

Zero-Point Corrected Electronic Energy:
-77.261266 Hartrees

Vibrational Frequencies:

Frequency (cm^{-1})	Intensity (km/mol)
576.0362	0.0003
576.1482	0.0656
797.6794	105.6703
2078.9893	0.0047
3430.1149	114.1511
3532.1199	0.0139

Electronic Transitions:

Wavelength (nm)	Oscillator Strength
175.74	0
175.74	0
161.38	0
146.49	0
146.48	0
143.39	0.0948
143.39	0.0948
124.87	0.7017

120.26	0.1427
120.26	0.1427
119.04	0.0002
119.04	0.0002
112.33	0
110.49	0
108.62	0
108.62	0.0002
95.42	0
95.42	0
95.06	0.0006
95.05	0
91.91	0
89.2	0.4909
86.82	0.0003
86.79	0
86.09	0
82.09	0.2128
78.71	0.1394
78.7	0.1397
77.23	0.0004
75.88	0.0564
75.88	0.0565
74.63	0.0953
74.54	0
71.78	0.0115
71.78	0.0117
71.28	0.1703
70.19	0.0155
70.18	0.0155
68.66	0.0003
68.66	0
68.39	0.1402
66.44	0.0001
66.44	0
66.16	0.0009
66.16	0
66.14	0
66.14	0
64.72	0
61.3	0.7288
60.52	0.0019
60.52	0.0016

60.25	0.5068
60.24	0.5089
59.23	0.0002
58.93	0.002
58.69	0.0003
58.69	0
57.42	0.5179
57.4	0.5214
56.73	0.025
56.39	0.0002
55.79	0.0027
54.01	0.0017
51.95	0.0001
50.98	0.0145
50.98	0.0142
50.88	0.0252
50.49	0.0041
50.35	0.1315
50.34	0.1291
50.21	0.0572
49.9	0.0012
49.9	0.0012
49.59	0.0291
48.79	0
48.79	0
48.12	0.1101
47.42	0
47.41	0.0015
46.9	0
46.9	0
46.84	0.0005
46.29	0
46.27	0.0109
46.2	0.1033
46.04	0.9872
46.03	0
46.03	0.9833
45.77	0
45.77	0
45.5	0
45.49	0.0002
45.24	0
45.24	0

44.4	0.0001
44.29	0.0086
42.93	0
42.91	0.0004
42.18	0.0002
40.64	0

Acetylene^{+}
B3LYP
m = 2

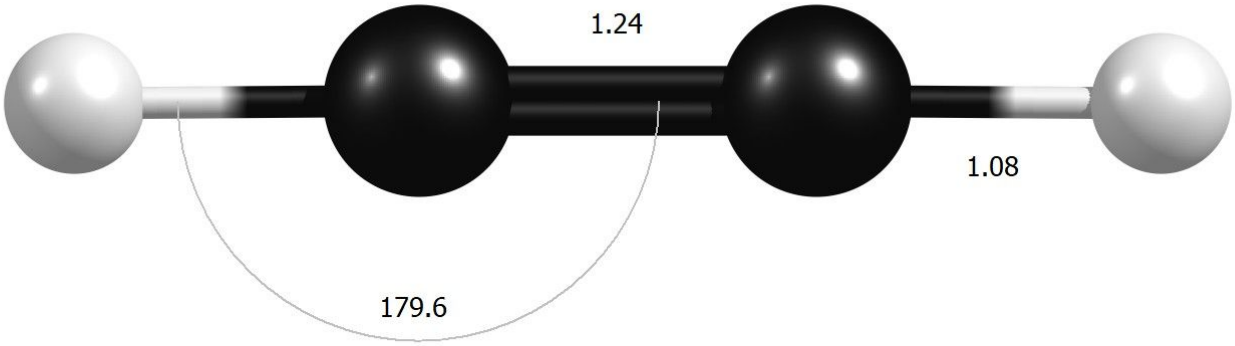

Coordinates:

6	-0.621681000	-0.001150000	-0.000002000
6	0.621680000	-0.001146000	0.000002000
1	-1.701277000	0.006894000	0.000004000
1	1.701284000	0.006885000	-0.000004000

Zero-Point Corrected Electronic Energy:
-76.926722 Hartrees

Vibrational Frequencies:

Frequency (cm^{-1})	Intensity (km/mol)
630.2142	0.0016
721.566	38.3939
793.577	0.0088
1894.2369	0.0034
3255.9473	443.4456
3354.6531	0.0208

Electronic Transitions:

Wavelength (nm)	Oscillator Strength
65290.32	0
239.01	0.0002
218.6	0.003
214.49	0
183.53	0.0006
170.46	0
166.05	0
157.14	0

122.58	0
115.15	0.6427
113.11	0.0005
110.84	0
107.28	0.0001
106.97	0
106.25	0.0003
104.81	0.013
103.19	0.0004
101.51	0.1683
101.31	0.0018
98.21	0
95.81	0.015
95.05	0.0001
94.55	0.0651
93.58	0
92.48	0
91.25	0
90.85	0
89.59	0.0451
88.54	0
88.5	0
86.32	0
85.17	0.0026
83.86	0
83.71	0
81.57	0.157
81.09	0
81.02	0.0003
80.48	0.1636
79.09	0.0006
78.19	0
77.78	0
75.97	0.0181
75.05	0.7581
72.65	0.0002
72.57	0
72.45	0
70.55	0
70.21	0
69.95	0
69.5	0
68.68	0

67.07	0.0026
66.79	0.0011
66.62	0.0003
66.6	0.0014
65.83	0.0064
65.8	0.0063
65.76	0
65.26	0.017
65.24	0.0004
65.19	0
65.12	0.0172
64.62	0
64.27	0.0133
63.77	0.0605
63.71	0.204
63.7	0.0508
63.56	0.0439
63.14	0.009
62.23	0.0313
60.79	0.0002
60.76	0.249
60.54	0
60.39	0.0006
59.96	0
59.79	0
59.66	0.354
59.6	0.2517
59.57	0.063
59.4	0
59.29	0.0649
59.18	0.2021
58.81	0.0913
58.74	0.5168
58.48	0.017
58.46	0
58.44	0
58.15	0.076
58.06	0
57.94	0
57.84	0.0011
57.83	0
57.15	0
56.89	0.0001

55.83	0.0142
55.08	0.0002
54.93	0.0002
54.7	0
54.45	0.0007
54.3	0.0005

Acetylene^{+}
M06
m = 2

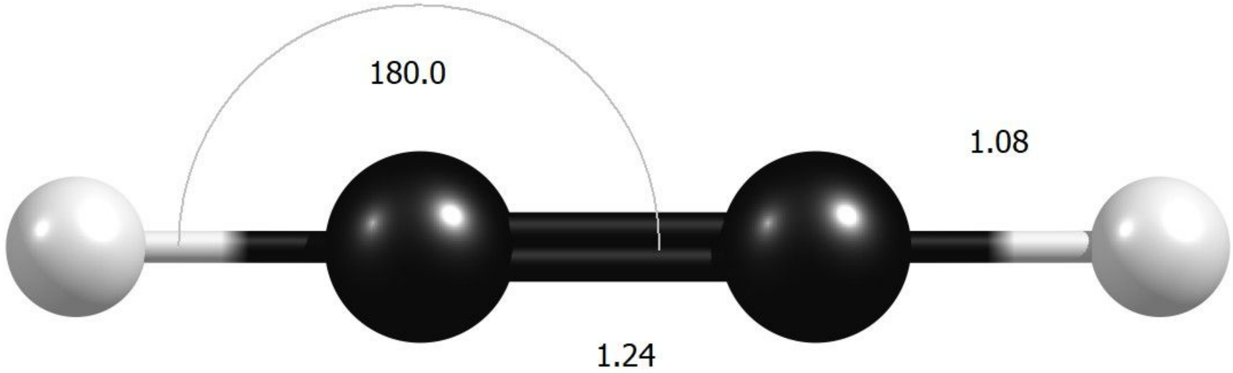

Coordinates:

6	-0.620994000	0.000096000	0.000005000
6	0.620993000	0.000105000	-0.000005000
1	-1.702700000	-0.000591000	-0.000011000
1	1.702702000	-0.000612000	0.000010000

Zero-Point Corrected Electronic Energy:
-76.865159 Hartrees

Vibrational Frequencies:

Frequency (cm^{-1})	Intensity (km/mol)
670.7237	0.0138
717.378	0.0282
756.5033	110.3046
1902.6275	0
3224.6822	479.3081
3328.9446	0.0002

Electronic Transitions:

Wavelength (nm)	Oscillator Strength
18481.05	0
235.4	0
212.81	0
210.72	0.0044
193.63	0.0003
174.54	0
174.02	0
164.9	0

126.06	0
117.4	0.6088
117.31	0.0248
114.57	0
111.91	0
110.4	0
108.76	0.0109
107.46	0.0059
106.9	0.0505
106.9	0
101.35	0.0991
101.07	0
101.05	0.0049
96.58	0
96.14	0.0552
95.53	0
94.55	0
94.29	0.0003
94.22	0
94.13	0
91.03	0
90.99	0
89.49	0.0873
88.27	0
87.46	0
87.11	0
86.94	0.0027
84.07	0
84.01	0.1619
83.98	0.0028
83.15	0
82.85	0
82.18	0.1117
80.41	0.0087
78.41	0
78.28	0.4262
76.87	0.2641
76.12	0
74.99	0
73.91	0
73.73	0
72.37	0
71.41	0

71.29	0
70.48	0.0161
70.12	0.0142
69.49	0.0193
68.97	0.0024
68.68	0.0128
68.5	0.016
67.75	0.0185
67.32	0
67.22	0
67.08	0.12
66.84	0.0251
66.54	0
66.42	0.0451
66.06	0.0524
65.88	0.0202
65.27	0.1576
64.65	0.0487
64.45	0.0338
64.22	0.0029
64.01	0
63.78	0.1811
63.62	0
63	0
62.7	0
62.64	0
62.49	0.0002
62.48	0.0032
62.25	0
62.08	0
61.71	0
61.49	0.0002
61.27	0
60.67	0
60.66	0
60.35	0
60.09	0.7019
60.03	0.7484
59.71	0
59.65	0
59.53	0.032
58.83	0
58.46	0

57.78	0.0002
57.69	0.0017
57.68	0
57.66	0.0002
56.8	0.0002
56.66	0.0064

Acetylene^{+}
M06-L
m = 2

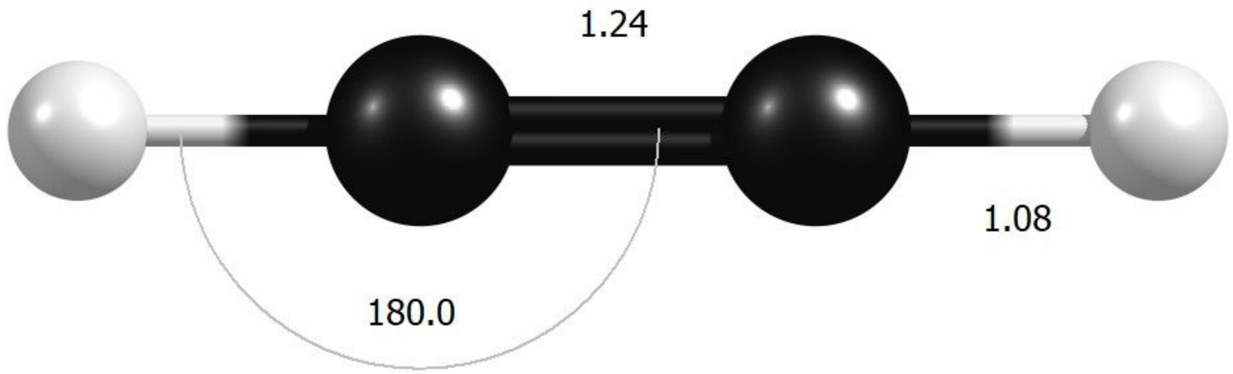

Coordinates:

6	-0.622311000	-0.000005000	0.000811000
6	0.622311000	-0.000032000	-0.000806000
1	-1.701954000	0.000080000	0.002134000
1	1.701959000	0.000140000	-0.002163000

Zero-Point Corrected Electronic Energy:
-76.916489 Hartrees

Vibrational Frequencies:

Frequency (cm^{-1})	Intensity (km/mol)
639.9464	3.1342
710.4187	20.6965
725.0328	31.1378
1898.9715	0
3256.3141	472.3835
3358.475	0

Electronic Transitions:

Wavelength (nm)	Oscillator Strength
9021.13	0
232.62	0
207.68	0
200.07	0.0045
182.93	0.0005
167.99	0
157.22	0
146.94	0

123.54	0
112.8	0.6297
112.21	0
110.06	0
107.38	0.0042
106.06	0
105.81	0
105.26	0.0058
99.74	0.0003
98.21	0.0985
97.73	0
94.01	0.061
93.14	0.0151
91.94	0
91.83	0.0005
89.04	0.0002
87.25	0.1195
87.2	0
86.3	0
86.14	0
85.82	0
85.45	0.0104
84.87	0
83.34	0
81.38	0.1853
80.48	0
80.45	0.1873
79.67	0
78.82	0
78.68	0
76.42	0.0214
76.41	0
75.95	0.783
75.41	0
73.7	0.0145
72.65	0
71.25	0
71.19	0
70.91	0.0129
70.52	0
70.36	0
69.41	0
69.01	0

67.95	0.0319
66.43	0.0088
66.41	0
65.22	0.0176
65.16	0.0115
64.82	0
64.74	0
64.5	0.039
64.31	0
64.3	0.0047
64.3	0.0005
64.16	0.08
64.14	0.001
63.98	0.0079
63.77	0.0299
63.4	0.3424
63.15	0.0266
63.13	0.1362
62.43	0.0157
60.67	0.0526
60.48	0.1827
60.42	0
59.56	0
59.55	0
59.25	0
59.11	0
58.95	0
58.69	0
58.68	0
58.57	0
58.53	0
58.32	0
58.08	0.0424
57.82	0
57.66	0.7078
57.61	0
57.57	0.5657
57.56	0
57.48	0.0008
57.44	0.0523
56.59	0
56.32	0
55.61	0.0001

55.46	0
54.9	0.0046
54.63	0.0004
54.42	0.0031
54.26	0
54.23	0.0998

Acetylene$^+$
MN15-L
m = 2

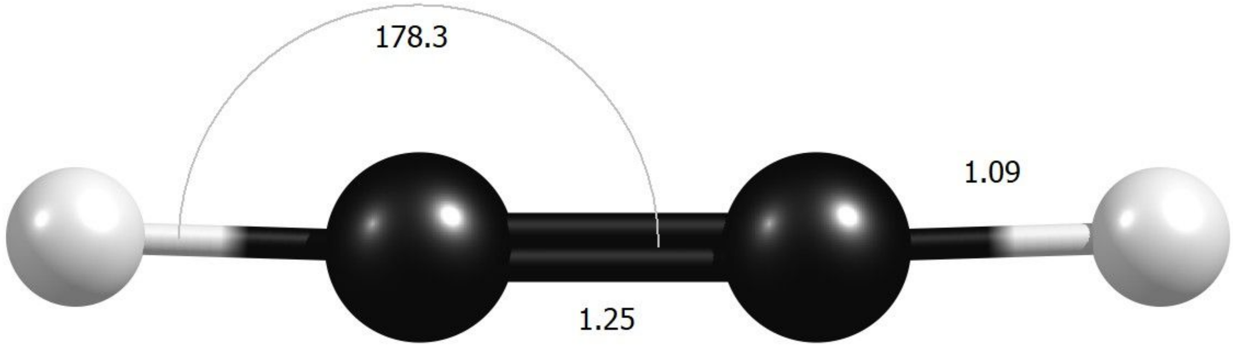

Coordinates:

6	-0.627461000	0.004714000	0.000003000
6	0.627462000	0.004714000	-0.000003000
1	-1.716054000	-0.028283000	-0.000007000
1	1.716051000	-0.028282000	0.000007000

Zero-Point Corrected Electronic Energy:
-76.860670 Hartrees

Vibrational Frequencies:

Frequency (cm^{-1})	Intensity (km/mol)
610.2384	0
725.3441	28.9852
746.184	0.1275
1897.8987	0.0538
3270.6543	487.3216
3372.5761	0.3331

Electronic Transitions:

Wavelength (nm)	Oscillator Strength
8851.63	0
224.16	0.0001
186.86	0
182.88	0.005
170.68	0
151.48	0
147.95	0
142.05	0

117.3	0
112.67	0.6602
109.35	0
105.06	0.0001
103.48	0.0224
102.9	0.0058
101.09	0.0126
100.34	0
99.25	0.0597
97.78	0.0022
96.34	0.1099
94.76	0
93.55	0.0689
91.95	0.0001
91.19	0.0294
89.99	0
88.36	0.0018
88	0
87.37	0
87.17	0.0016
86.65	0
84.45	0
83.43	0.0008
83.14	0.0734
81.59	0
81.26	0.0196
80.55	0.0009
78.65	0.0001
78.35	0.1865
76.67	0
76.28	0.0346
76.07	0.1284
75.85	0
74.76	0.0129
74.34	0.8212
72	0.0122
71.56	0
70.98	0
70.84	0
69.9	0.0005
69.11	0
68.83	0
68.22	0.0001

66.45	0.0003
65.45	0.0128
65.01	0.0085
64.94	0.0002
64.77	0.0155
64.53	0.0379
63.99	0.0001
63.94	0.0329
63.19	0.0039
63.13	0.0241
63.05	0.009
62.5	0.0026
62.46	0.1279
61.69	0.0762
61.56	0.0585
61.23	0.02
61.19	0
60.65	0
60.59	0.0182
60.22	0.3119
59.69	0
59.59	0.0086
59.19	0.0001
59.02	0.0401
58.77	0.0008
58.68	0.0001
58.4	0
58.22	0
58.18	0.0123
57.82	0.0003
57.79	0.0008
57.25	0
57.24	0.0039
56.84	0.0872
56.79	0
56.51	0
56.49	0.6375
56.46	0.5492
56.08	0
56.06	0.0917
56	0.2283
55.52	0
55.39	0.0462

54.67	0.002
54.53	0.0005
53.84	0.0039
53.81	0.0001
53.63	0.2145
53.5	0.0073

Ethylene
B3LYP
m = 1

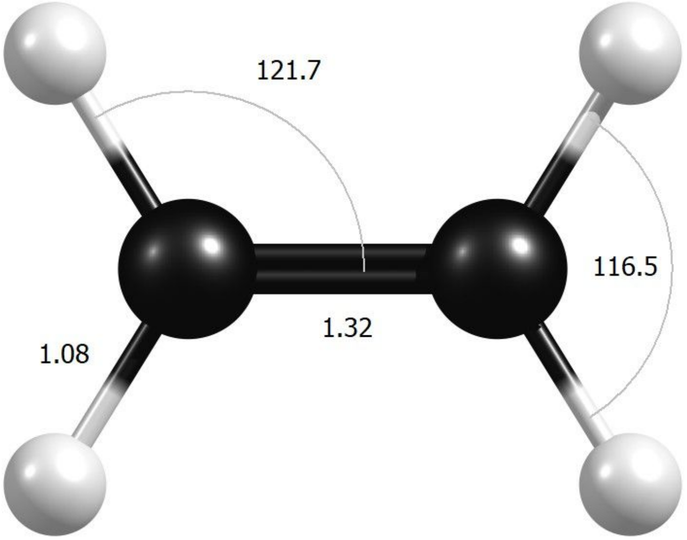

Coordinates:

6	0.000000000	0.662376000	0.000000000
6	0.000000000	-0.662373000	0.000000000
1	0.921372000	1.232409000	0.000000000
1	-0.921384000	1.232427000	0.000000000
1	0.921385000	-1.232432000	0.000000000
1	-0.921373000	-1.232423000	0.000000000

Zero-Point Corrected Electronic Energy:
-78.572809 Hartrees

Vibrational Frequencies:

Frequency (cm^{-1})	Intensity (km/mol)
837.214	0.2297
978.714	102.5818
983.2505	0.0013
1062.9816	0
1246.4274	0
1382.9646	0
1479.2773	9.3344
1692.5305	0
3130.1356	16.9388
3143.1501	0.0001
3199.1389	0

3227.3585	21.4475

Electronic Transitions:

Wavelength (nm)	Oscillator Strength
297.75	0
169.99	0
166.42	0
165.81	0.054
162.43	0.3514
156.5	0
154.82	0
153.9	0
152.72	0
150.95	0
138.43	0
133.84	0
128.97	0
128.9	0
128.77	0
128.18	0
127.08	0
125.57	0
125.02	0
124.48	0
123.75	0.0004
121.23	0
120.67	0.1405
118.11	0
117.51	0.1726
112.74	0
112.38	0
112.26	0
111.03	0
110.64	0
106.92	0
106.88	0
106.67	0
106.13	0
105.75	0
105.24	0
104.86	0
104.26	0.3412

103.76	0
102.84	0
102.56	0.2499
102.44	0
101.95	0
101.32	0.0738
101.04	0
99.62	0
98.73	0.0221
96.54	0.205
95.2	0
94.6	0
94.17	0
94.1	0
92.49	0
92.27	0
92.13	0
91.76	0.2475
91.07	0
90.59	0
90.24	0
90.05	0
90.03	0
89.05	0.0189
88.99	0
88.38	0
87.34	0
87.18	0
86.41	0.3304
86.25	0
85.53	0
84.48	0
84.4	0
83.02	0
82.8	0.3555
81.91	0
81.83	0
81.5	0
81.37	0.0277
81.29	0
81.16	0.0774
81.16	0
80.78	0.0093

80.74	0
80.59	0
80.02	0
79.81	0
79.54	0
78.35	0.0045
78.31	0
78.12	0
77.83	0
77.7	0
77.52	0
77.39	0
77.22	0
77.15	0
76.75	0
76.73	0
76.73	0.0029
76	0
75.97	0.1854

Ethylene
M06
m = 1

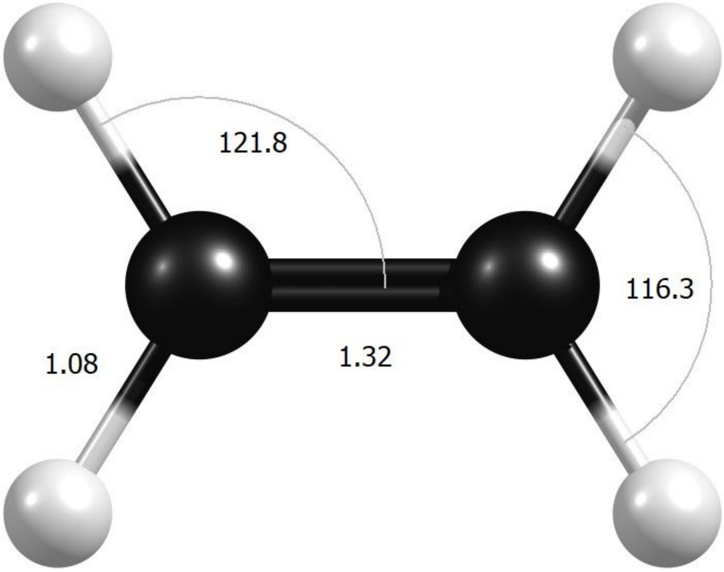

Coordinates:

6	0.000000000	0.659494000	0.000000000
6	0.000000000	-0.659490000	0.000000000
1	0.920995000	1.231612000	0.000000000
1	-0.920999000	1.231624000	0.000000000
1	0.920995000	-1.231629000	0.000000000
1	-0.920986000	-1.231636000	0.000000000

Zero-Point Corrected Electronic Energy:
-78.496390 Hartrees

Vibrational Frequencies:

Frequency (cm^{-1})	Intensity (km/mol)
813.1337	0.1976
966.1502	94.8416
975.5422	0.0002
1058.7195	0
1216.7203	0
1364.2986	0
1444.9718	8.2393
1697.1755	0
3125.8335	14.4395
3140.4983	0

3207.3354	0
3232.3097	16.1115

Electronic Transitions:

Wavelength (nm)	Oscillator Strength
290.2	0
176.91	0
173.23	0.0388
168.5	0
165.89	0
164.99	0.3301
164.74	0
161.97	0
161.97	0
157.67	0
141.56	0
140.78	0
137.59	0
136.63	0
133.4	0
132.37	0
131.41	0
129.09	0
129.08	0
128.98	0
128.83	0.0042
127.85	0.0858
124.02	0
123.17	0.1272
120.35	0
119.43	0
117.61	0
115.17	0
114.07	0
114.07	0
112.92	0
110.62	0
110.22	0
109.93	0
108.92	0
108.76	0
108.12	0

107.73	0.3401
107.47	0.2195
106.99	0
106.54	0
106.39	0
105.99	0.0515
105.27	0
104.91	0
101.97	0.0265
101.84	0
99.88	0
99.63	0.035
98.47	0
97.71	0
97.63	0
97.62	0
97.27	0.4293
96.35	0
96.2	0
95.5	0
94.05	0
93.95	0
93.94	0
92.59	0
92.46	0
92.32	0
92.24	0
91.29	0
91.27	0
91.09	0.0227
89.8	0
89.71	0.313
88.69	0
88.2	0
88.04	0
87.95	0
86.77	0.0236
85.63	0.0568
85.44	0.2206
85.08	0
83.73	0
83.56	0.0291
83.47	0

83.08	0.097
82.98	0
82.91	0
82.66	0
82.51	0
82.33	0
82.26	0
81.88	0
81.72	0
81.58	0
80.84	0.0002
80.59	0
80.42	0
80.28	0
80.06	0.0554
80.03	0
80.01	0
79.93	0
79.27	0
78.94	0

Ethylene
M06-L
m = 1

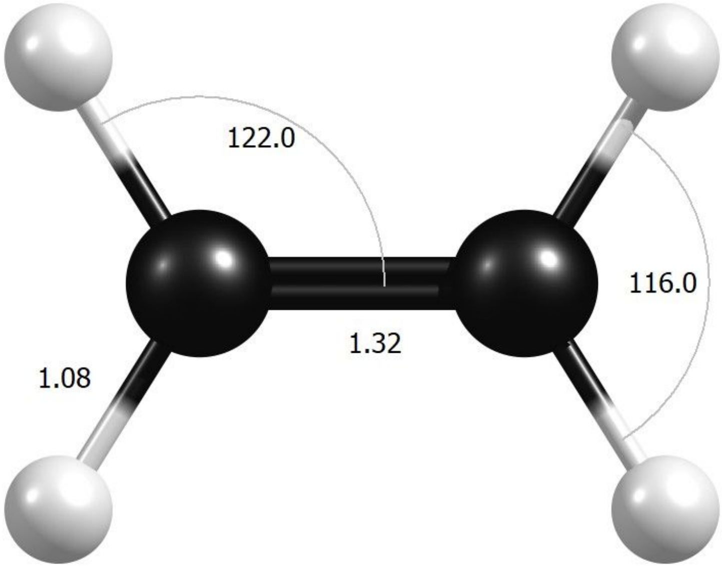

Coordinates:

6	0.000000000	0.660235000	0.000000000
6	0.000000000	-0.660231000	0.000000000
1	0.918539000	1.233641000	0.000000000
1	-0.918548000	1.233657000	0.000000000
1	0.918547000	-1.233663000	0.000000000
1	-0.918536000	-1.233658000	0.000000000

Zero-Point Corrected Electronic Energy:
-78.551080 Hartrees

Vibrational Frequencies:

Frequency (cm^{-1})	Intensity (km/mol)
824.9585	0.2346
967.441	0.0004
975.1992	92.7407
1079.8066	0
1237.6711	0
1384.6898	0
1468.1134	5.8859
1700.579	0
3139.9517	14.6589
3156.6579	0

3222.3942	0
3248.4687	18.846

Electronic Transitions:

Wavelength (nm)	Oscillator Strength
278.7	0
162.23	0
155.43	0.3335
153.24	0
151.89	0.0498
151.27	0
147.88	0
144.15	0
143.81	0
142.4	0
134.76	0
125.83	0
124.46	0
124.32	0
123.26	0
122.88	0
119.99	0
119.71	0
117.61	0.1191
115.85	0
115.13	0.0091
114.6	0.1858
114.57	0
113.7	0
111.7	0
110.57	0
107.62	0
107.17	0
107	0
104.9	0
104.8	0.3387
104.45	0
104.24	0
102.25	0
101.42	0
101.16	0
100.7	0.2786

99.8	0
99.47	0
99.27	0.0487
98.24	0
98.01	0
97.21	0
96.57	0
95.68	0
95.07	0
94.16	0.1282
93.88	0.01
93.74	0
93.36	0
93.11	0
92.92	0
91.65	0
90.92	0.3985
90.17	0
89.74	0
89.73	0
89.09	0
88.93	0
88.22	0
88.19	0
88.06	0
86.22	0
85.27	0
85.15	0
84.67	0.3402
84.66	0
84.52	0
84.39	0.0114
83.81	0
83.24	0
83.2	0
82.43	0.3126
82.22	0
81.31	0.0459
80.71	0.0932
80.31	0
79.84	0
79.3	0
79.21	0.002

79.17	0
79.16	0
79.1	0.0214
78.57	0
77.92	0
77.5	0
77.4	0
77.26	0
77.25	0.0407
77.23	0
76.58	0
76.43	0
76.33	0
76.16	0
76.14	0
75.9	0
75.84	0
75.74	0.06
75.54	0
75.41	0.0984

Ethylene
MN15-L
m = 1

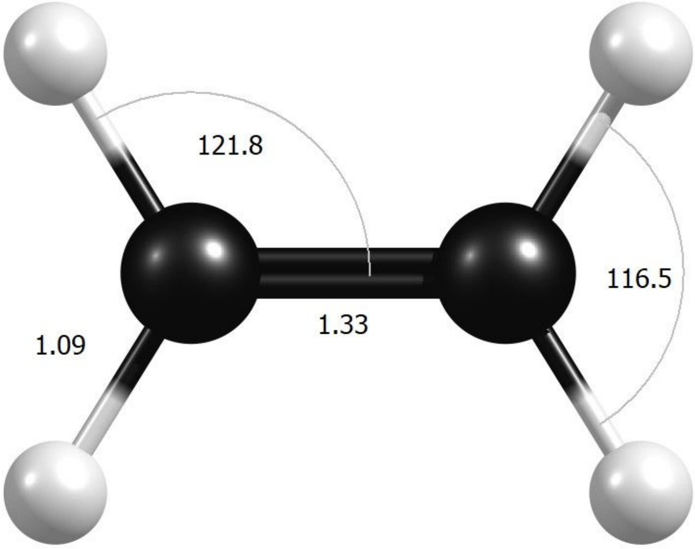

Coordinates:

6	0.000000000	0.667049000	0.000000000
6	0.000000000	-0.667048000	0.000000000
1	0.930541000	1.243430000	0.000000000
1	-0.930539000	1.243432000	0.000000000
1	0.930538000	-1.243433000	0.000000000
1	-0.930538000	-1.243435000	0.000000000

Zero-Point Corrected Electronic Energy:
-78.479022 Hartrees

Vibrational Frequencies:

Frequency (cm^{-1})	Intensity (km/mol)
816.0123	0.0207
954.8557	0
983.9394	94.3072
1084.5623	0
1242.5656	0
1388.36	0
1471.2651	4.3187
1691.4017	0
3117.879	20.2554
3137.6965	0

3200.6408	0
3231.1663	32.0024

Electronic Transitions:

Wavelength (nm)	Oscillator Strength
272.69	0
162.3	0
159.4	0.0701
157.21	0.3778
155.81	0
151.74	0
148.34	0
147.45	0
146.28	0
145.54	0
131.53	0
127.77	0
127.08	0
124.69	0
124.66	0
124.44	0
124.09	0
123.24	0
123.15	0
120.93	0
120.16	0.1587
118.58	0
117.82	0.0024
116.69	0.2045
111.06	0
110.31	0
108.9	0
108.53	0
108.22	0
107.52	0
105.6	0
105.14	0
104.41	0
103.35	0
103.32	0.3835
102.63	0
102.13	0

102.09	0
102.01	0.2891
101.45	0
101.09	0
99.62	0
99.49	0.0813
99.47	0
99.46	0
97.55	0
96.27	0.0026
95.25	0.2114
94.46	0
93.52	0
92.74	0
91.8	0
91.74	0.2935
90.39	0
90.28	0
89.9	0
89.88	0
88.96	0
88.43	0
87.9	0
86.82	0
86.77	0
86.7	0
85.95	0.0288
85.94	0
85.16	0
84.88	0
84.86	0.3197
84.17	0
84.04	0
83.49	0
82.38	0
81.05	0
81.05	0.0759
80.76	0.0373
79.85	0
79.69	0.3128
79.13	0
79.1	0
79.03	0.0239

79.01	0
78.24	0
78	0
77.93	0
77.63	0
77.21	0
77.09	0
77.03	0
76.82	0
76.62	0
76.42	0
75.98	0.008
75.9	0
75.86	0.0024
75.61	0
75.47	0
75.06	0
74.69	0.0076
74.49	0
74.35	0

Ethylene^{+}
B3LYP
m = 2

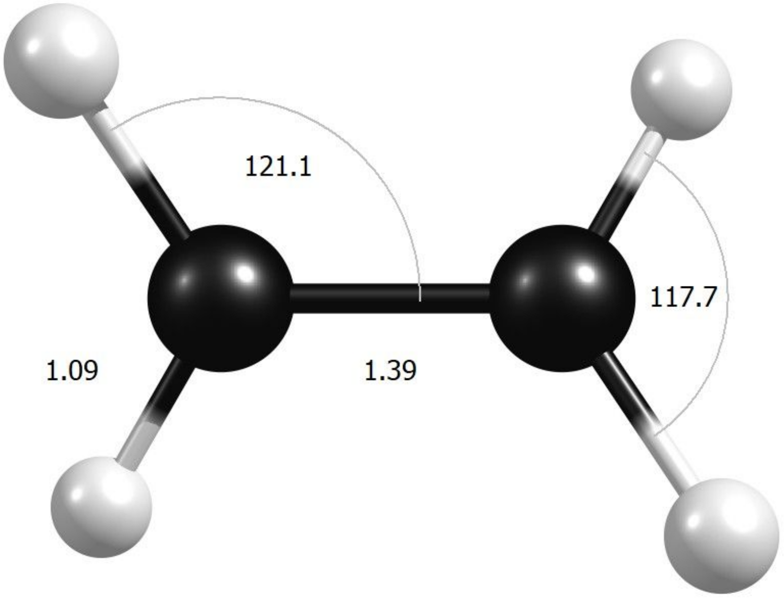

Coordinates:

6	0.693900000	0.000000000	-0.000001000
6	-0.693905000	0.000004000	0.000001000
1	-1.257573000	0.903566000	0.230739000
1	-1.257537000	-0.903584000	-0.230751000
1	1.257553000	-0.903566000	0.230752000
1	1.257587000	0.903563000	-0.230743000

Zero-Point Corrected Electronic Energy:
-78.195063 Hartrees

Vibrational Frequencies:

Frequency (cm^{-1})	Intensity (km/mol)
612.5275	0
787.7682	5.3693
888.493	40.7678
1079.1691	0.7639
1205.5192	12.4367
1274.4899	0
1431.1793	55.1674
1502.5979	0
3085.6264	0.0001

3089.4011	85.3601
3180.2591	3.3907
3200.6814	85.0507

Electronic Transitions:

Wavelength (nm)	Oscillator Strength
344.56	0
261.97	0.0005
252.24	0.0275
205.59	0.0428
177.46	0.0192
155.6	0.0023
139.66	0.133
137.22	0.007
133.05	0
127.3	0.0078
123.58	0.0124
115.54	0
114.04	0.0618
113.51	0.0065
103.28	0.0001
101.55	0.0023
99.92	0
98.41	0.0286
98.37	0.0011
97.58	0.0009
95.13	0.006
93.86	0.0008
93.82	0
93.11	0.0543
92.04	0
91.79	0.1491
90.93	0.1381
88.58	0.0343
87.62	0
87.15	0.0036
87.03	0.0012
86.48	0.0017
86.37	0
84.9	0.0009
83.87	0.0091
83.67	0.0323

83.18	0.3711
83.1	0.1774
82.55	0.0122
82.25	0
81.88	0.1757
81.64	0
81.01	0.0206
80.8	0.0743
80.28	0.0002
79.37	0.0064
78.43	0.0004
78.06	0
77.91	0
77.66	0.0037
77.52	0.0151
77.28	0.3225
77.24	0.0142
76.45	0
76.02	0.0082
75.49	0.0016
75.45	0.1692
74.98	0
74.02	0.182
73.28	0.0251
72.85	0.0002
72.76	0
72.28	0.0679
72.11	0.0172
71.8	0
71.72	0.1101
71.62	0.015
71.55	0.0301
71.19	0.061
70.84	0.0258
70.55	0.0024
70.43	0.0318
70.28	0
70.03	0.0231
69.81	0.0069
69.65	0.0001
69.56	0
69.13	0.0057
68.79	0.0009

68.44	0.044
68.41	0.0008
68.22	0.0002
67.52	0
67.52	0.022
67.31	0.1117
67.05	0.0005
66.92	0.0001
66.62	0.0052
66.57	0.0001
66.53	0.0302
66.45	0
66.44	0
66.22	0.0055
65.01	0.3396
64.92	0.0006
64.75	0
64.49	0.0005
64.29	0
64.16	0.007
63.59	0

Ethylene^{+}
M06
m = 2

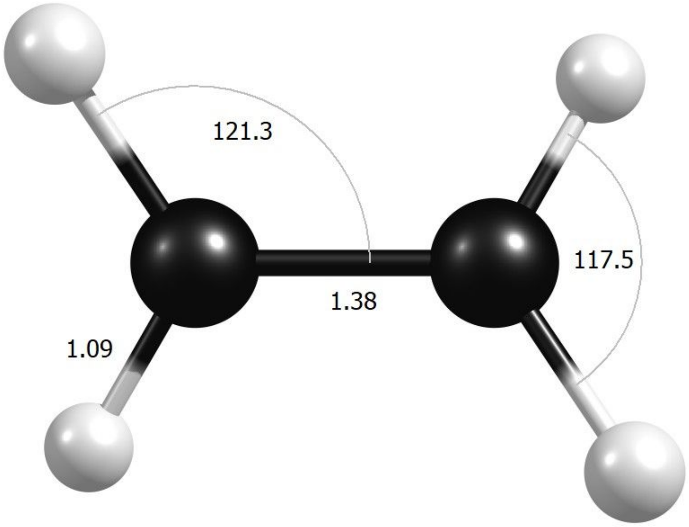

Coordinates:

6	0.692084000	0.000000000	0.000002000
6	-0.692090000	0.000000000	0.000004000
1	-1.258004000	0.905238000	0.223601000
1	-1.257961000	-0.905262000	-0.223626000
1	1.258008000	-0.905226000	0.223610000
1	1.257990000	0.905250000	-0.223623000

Zero-Point Corrected Electronic Energy:
-78.122589 Hartrees

Vibrational Frequencies:

Frequency (cm^{-1})	Intensity (km/mol)
569.8778	0
764.6853	4.5588
861.4642	35.2663
1021.4671	0.0818
1165.3727	8.8349
1270.8043	0
1387.4375	54.4435
1483.4006	0
3065.5249	0.0001
3069.0042	94.9862
3168.2273	3.1995

3186.6799	95.3619

Electronic Transitions:

Wavelength (nm)	Oscillator Strength
347.14	0
260.28	0.0337
257.54	0.0007
208.05	0.0295
176.34	0.0316
155.64	0.0041
145.55	0.0082
139.81	0.1132
132.4	0
129.49	0.0096
127.04	0.0044
120.63	0.0565
119.58	0.0041
114.58	0
107.56	0.0002
106.14	0
105.88	0.0006
102.29	0.018
102.06	0.0048
100.68	0.0082
100.01	0.0016
99.07	0
98.43	0.0001
96.14	0.1017
95.58	0.1068
94.89	0
94.89	0.025
92.43	0.045
91.26	0
90.15	0.0024
90.14	0
90.01	0.0097
89.03	0.0029
87.67	0.0021
87.38	0.1162
86.71	0
86.49	0.0539
86.16	0.0572

85.9	0.3149
85.55	0.045
85.14	0
84.61	0.0397
84.07	0.0138
83.84	0.0028
83.33	0.0137
83.17	0.108
82.19	0
81.96	0
81.14	0.0015
80.79	0.0037
80.61	0.3633
80.56	0.0006
80.03	0.0049
79.72	0.0532
79.07	0
78.2	0.0939
77.68	0.1109
77.45	0.001
76.97	0
76.89	0.0844
76.71	0.0734
76.5	0.0018
76.1	0.0019
75.34	0.0171
75.1	0.0142
74.71	0.0756
74.54	0.0266
74.33	0
74.1	0.1429
74.01	0
73.53	0.0029
73.29	0
73.06	0.0179
72.96	0.0186
72.47	0.0004
72.31	0.0001
71.99	0
71.99	0.0128
71.98	0.0064
71.38	0.0005
71.31	0.0004

70.99	0.047
70.87	0.0073
70.61	0.0531
70.52	0.0178
69.73	0.0756
69.64	0.0013
69.63	0
69.39	0.0188
69.21	0.0177
68.97	0.0461
68.78	0
68.7	0
68.31	0
68.13	0.0084
67.2	0
67.18	0.1714
67.01	0
66.47	0.0045
66.35	0

Ethylene^{+}
M06-L
m = 2

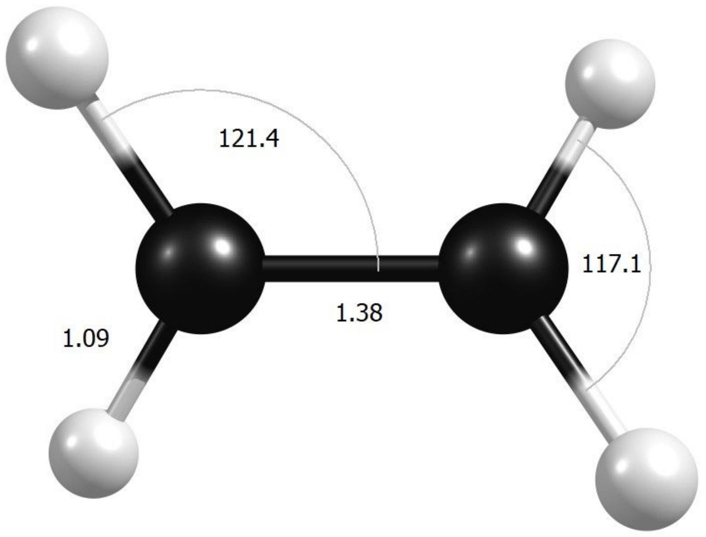

Coordinates:

6	0.690823000	-0.000002000	0.000001000
6	-0.690827000	-0.000001000	0.000001000
1	-1.259641000	0.899867000	0.235263000
1	-1.259638000	-0.899869000	-0.235267000
1	1.259658000	-0.899852000	0.235264000
1	1.259640000	0.899869000	-0.235270000

Zero-Point Corrected Electronic Energy:
-78.177431 Hartrees

Vibrational Frequencies:

Frequency (cm^{-1})	Intensity (km/mol)
640.9187	0
765.5094	4.7925
857.9626	33.5406
1021.404	0.1195
1173.1605	10.1467
1280.1205	0
1392.5297	52.7132
1481.2855	0
3080.4209	0.0005
3081.4019	95.098
3180.7948	3.5685

3200.7951	91.1645

Electronic Transitions:

Wavelength (nm)	Oscillator Strength
320.46	0
251.91	0.0222
237.31	0.0006
195.64	0.0433
174.85	0.03
152.84	0.0039
139.29	0.0134
137.08	0.1314
132.65	0
123.87	0.0012
117.48	0.0053
112.72	0
110.44	0.0088
110.27	0.082
104.69	0.001
99.6	0.0024
98.15	0.0183
95.6	0.0255
95.46	0.0083
95.19	0
91.16	0.0523
91.01	0.0771
90.62	0.1441
90.54	0
90.21	0.009
89.6	0.0034
89.24	0
86.61	0.0004
86.3	0.0076
86	0
85.24	0.0035
85.05	0.0213
84.61	0
83.67	0.2578
83.59	0.001
82.48	0.0777
82.44	0.3134
82.16	0

81.24	0.0592
81.24	0.0504
80.89	0.0003
80.25	0
79.63	0.1403
79.38	0.004
79.11	0.03
77.97	0.022
77.52	0
77.5	0.0067
77.07	0
77.02	0.0008
76.66	0.3609
76.38	0.0295
75.95	0
75.64	0.0247
75.2	0.0008
74.04	0.1321
73.9	0.0476
73.4	0.0529
72.05	0.0348
72	0
71.61	0.0015
71.54	0.1218
71.39	0.0072
71.29	0
71.2	0.1829
71.01	0.0132
71	0.0044
70.77	0.0121
70.62	0.0788
70.19	0.0217
69.97	0
69.82	0.0081
69.61	0.0123
69.31	0
69.27	0.0004
69.17	0.0203
68.64	0.0001
68.54	0.0109
68.1	0.0129
68.02	0
67.86	0.0015

67.59	0.0114
67.57	0.0007
67.23	0.0017
67.19	0.0688
66.91	0.0241
66.9	0
66.54	0.001
66.41	0.0626
65.87	0.0203
65.86	0.0462
65.77	0
65.55	0
65.14	0.0028
64.97	0.0118
64.91	0.017
64.51	0.0007
64.48	0.0319
64.25	0.0001
64.22	0

Ethylene$^+$
MN15-L
m = 2

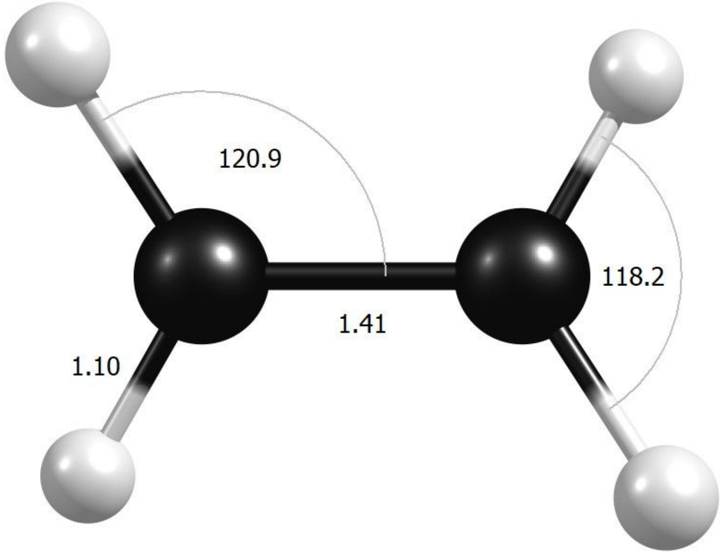

Coordinates:

6	0.705886000	0.000001000	-0.000002000
6	-0.705886000	0.000002000	0.000000000
1	-1.270387000	0.924308000	0.185651000
1	-1.270404000	-0.924303000	-0.185650000
1	1.270387000	-0.924313000	0.185656000
1	1.270407000	0.924292000	-0.185646000

Zero-Point Corrected Electronic Energy:
-78.110186 Hartrees

Vibrational Frequencies:

Frequency (cm^{-1})	Intensity (km/mol)
463.3351	0
793.8046	3.344
914.6321	31.7125
1064.9464	0.1107
1214.0345	6.3846
1279.4202	0
1438.6318	35.6917
1516.8853	0
3110.4421	72.8065
3114.3857	0.0003

3218.6882	1.7501
3238.4216	69.1064

Electronic Transitions:

Wavelength (nm)	Oscillator Strength
298.71	0
228.94	0.001
219.91	0.0411
182.49	0.0494
166.18	0.0427
144.41	0.0023
129.61	0.0084
127.66	0.0811
124.44	0
117.68	0.0171
116.35	0.0008
108.76	0.0107
108.69	0.0585
103.1	0
101.78	0.0001
98.33	0.0015
97.49	0.0308
96.64	0.006
95.68	0
95.33	0.0006
91.58	0.171
91.56	0
90.81	0.1879
90.81	0.0242
90.07	0.0293
89.7	0
89.1	0.0001
86.44	0
85.2	0.007
85.17	0.0145
84.84	0.0001
84.46	0
83.21	0.0015
82.75	0.3822
82.1	0.0446
81.59	0
81.49	0.0307

81.44	0.0416
81.1	0.0006
80.66	0.1784
80.32	0
79.48	0.112
79.42	0.0891
79.26	0.0692
78.72	0.0038
78.2	0
77.74	0
77.13	0.0215
76.98	0.0238
76.76	0.0382
76.64	0.0446
76.23	0.3128
75.78	0.021
75.4	0
74.38	0.0002
73.84	0.1479
73.7	0.0039
73.7	0
73.4	0.1738
72.19	0.0053
72.07	0.0107
71.38	0.0199
71.33	0.0182
70.88	0
70.81	0.0261
70.67	0
70.3	0.1423
70.18	0.0035
69.86	0.0376
69.6	0.0801
69.48	0.0643
69.02	0.0158
68.43	0
68.18	0.0001
68.07	0.0074
67.89	0.0064
67.76	0.006
67.6	0.0031
66.85	0
66.82	0.0177

66.63	0.0268
66.48	0.0412
66.32	0
66.15	0.0112
66.05	0.039
65.83	0
65.76	0.037
65.72	0.0007
65.02	0
64.95	0
64.89	0.0002
64.78	0.0124
64.24	0.0039
63.88	0.0077
63.75	0
63.6	0.2102
62.91	0.0173
62.53	0.0122
62.47	0.0001
62.37	0

$Zn^+(C_2H_2)$ Isomer 1
B3LYP
m=2

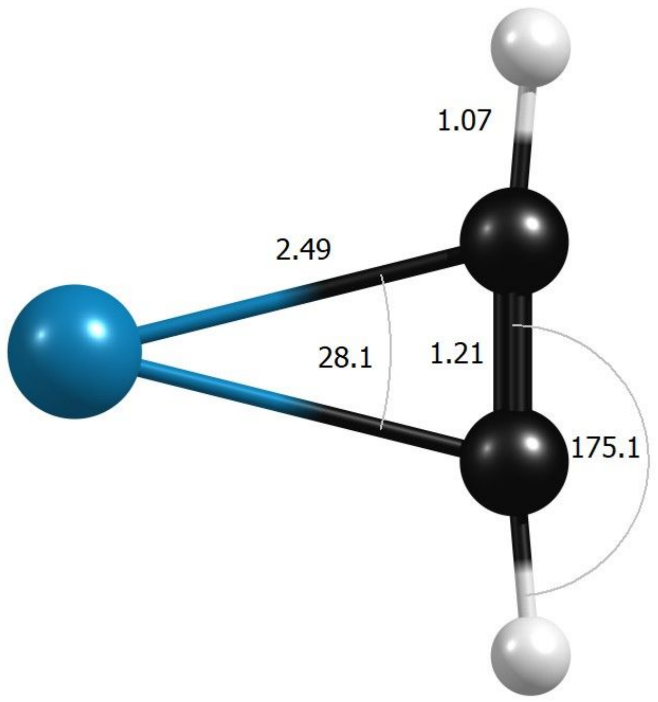

Coordinates:

30	0.773741000	-0.000001000	0.000000000
6	-1.645072000	-0.604388000	0.000000000
6	-1.645054000	0.604394000	-0.000001000
1	-1.735756000	-1.671725000	0.000002000
1	-1.735735000	1.671731000	0.000003000

Zero-Point Corrected Electronic Energy:
-1856.486908 Hartrees

Vibrational Frequencies:

Frequency (cm^{-1})	Intensity (km/mol)
48.8365	6.5145
204.7084	13.867
625.8715	0
696.0643	4.7942
779.0227	94.5479
860.7114	123.6695
2013.5054	70.1081
3332.0116	247.1801

3434.3933	1.7649

Electronic Transitions:

Wavelength (nm)	Oscillator Strength
363.32	0.0007
347.5	0.0255
267.74	0.0739
253.05	0.1176
240.88	0
230.03	0.0118
225.12	0
210.03	0
209.67	0.0224
189.63	0.4295
187.33	0.0362
170.9	0.0139
167.02	0.0461
159.03	0
155.06	0.0001
154.8	0.0498
153.49	0.0226
153.15	0.0007
152.77	0.0016
152.57	0.0011
152.54	0
151.37	0.0528
147.59	0.0009
147.52	0
145.39	0
142.45	0.001
141.25	0.0001
139.69	0
138.66	0.1197
135.82	0.0018
134.18	0.3848
133.75	0.0545
132.46	0.0059
129.1	0.0001
127.32	0.0003
127.28	0.002
126.83	0
121.5	0.0007

121.45	0.0002
121.2	0.0226
120.51	0
119.93	0.0284
119.01	0.0235
118.64	0
118.54	0.0092
118.17	0.0897
115.96	0.0883
115.41	0.0013
113.38	0.0008
112.71	0.0936
110.99	0.001
110.65	0.0135
110.56	0.0032
110.33	0.0153
110.09	0.0007
110.08	0
109.97	0.0027
109.82	0.0034
108.82	0.0106
106.87	0
106.76	0.0658
106.67	0.0002
106.45	0.0387
106.39	0.0252
106.03	0.0385
105.7	0.038
105.45	0
104.95	0.0286
104.45	0.0117
104	0.0026
103.59	0.0003
103.35	0.165
102.16	0.0188
101.9	0.0129
101.68	0.0008
101.61	0
101.57	0.0142
101.32	0.0001
100.96	0.0072
100.36	0.0005
99.59	0.0011

98.93	0.0111
98.2	0
97.52	0.0001
97.18	0
97.14	0.0067
97.07	0.0008
97.04	0.0045
96.85	0.0539
96.55	0.0346
96.49	0.0313
96.46	0.0007
96.42	0
95.79	0.0111
95.76	0
95.55	0
95.54	0.0001
95.19	0.0172
94.69	0.0001
94.1	0.0127

$Zn^+(C_2H_2)$ Isomer 2
B3LYP
m=4

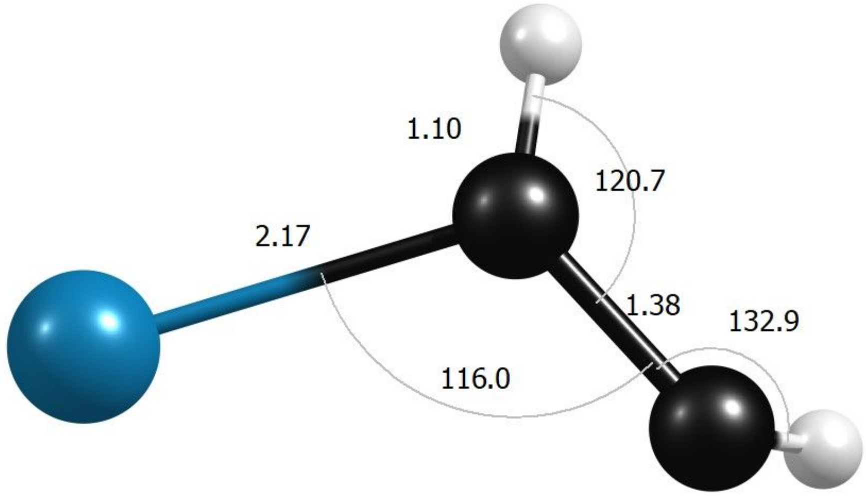

Coordinates:

30	0.832766000	-0.054298000	0.009794000
6	-1.244836000	0.570298000	-0.125132000
6	-2.182289000	-0.439927000	-0.089804000
1	-1.421869000	1.518912000	0.400008000
1	-2.998371000	-0.672200000	0.595792000

Zero-Point Corrected Electronic Energy:
-1856.360719 Hartrees

Vibrational Frequencies:

Frequency (cm^{-1})	Intensity (km/mol)
167.111	4.0823
306.8539	0.6257
391.5749	214.3238
838.9548	20.2985
947.414	7.8508
1036.7985	49.451
1359.3759	5.0077
3013.2561	13.8485
3116.1297	35.9373

Electronic Transitions:

Wavelength (nm) Oscillator Strength

634.11	0.0014
530.06	0.0398
380.72	0.0274
284.86	0.0685
275.59	0.0711
252.01	0.1635
241.04	0.0006
228.91	0.0259
212.41	0.0018
204.71	0.0526
201.52	0.0637
193.83	0.0124
189.53	0.1737
172.87	0.0052
170.21	0.1371
166.95	0.0061
166.74	0.0134
165.76	0.0004
165.73	0.0019
164.61	0.0293
159.09	0.0034
158.35	0.0117
155.81	0.0677
154.79	0.0201
149.87	0.0349
148.14	0.0066
146.39	0.0023
146.04	0.0068
145.31	0.001
145.27	0.0017
145.18	0.0078
143.97	0.02
143.64	0.0099
143.22	0.0285
139.83	0.019
138.19	0.0246
136.68	0.0124
134.68	0.0016
134.3	0.0005
133.93	0.0398
132.88	0.0998
131.73	0.1422
131.08	0.0739

130.47	0.0492
128.26	0.0047
126.6	0.0045
125.06	0.0195
122.57	0.0296
121.24	0.0034
120.68	0.0127
120.62	0.0216
119.87	0.0134
119.12	0.0231
117.58	0.0051
117.37	0.0056
117.07	0.0031
116.92	0.0006
116.91	0.0014
116.67	0.0184
116.1	0.0136
115.96	0.0154
115.57	0.011
115.45	0.0098
115.36	0.0028
115.22	0.0215
114.69	0.0253
113.56	0.021
112.31	0.0088
112.25	0.0209
111.07	0.019
110.62	0.0208
109.87	0.0166
108.31	0.0124
108.24	0.0126
107.35	0.0034
106.29	0.005
106.24	0.0294
105.89	0.0005
105.14	0.0021
104.86	0.0048
104.25	0.0158
103.96	0.0005
103.78	0.0024
103.66	0.0035
103.43	0.0164
103.34	0.0112

103.12	0.009
103.11	0.0074
103.06	0.0205
102.86	0.004
102.6	0.0245
102.47	0.0158
102.18	0.01
102	0.0014
101.75	0.0067
101.43	0.0776
100.77	0.0151
100.53	0.0018
100.45	0.0135
100.36	0.0138

$Zn^+(C_2H_2)$ Isomer 3
B3LYP
m=2

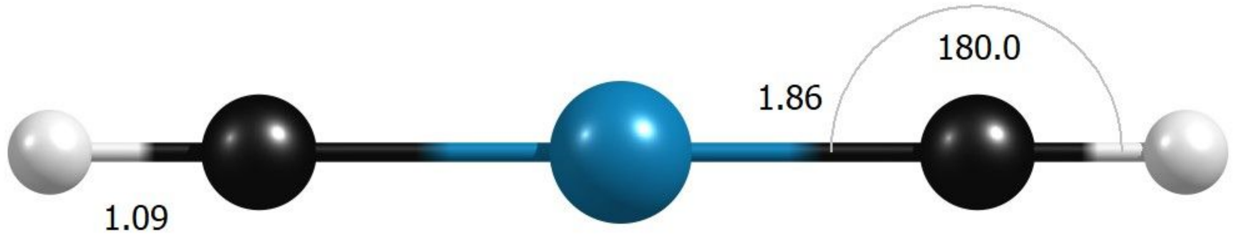

Coordinates:

30	0.004153000	-0.000043000	0.000045000
6	1.859926000	0.000040000	0.012956000
6	-1.877318000	0.000035000	-0.013174000
1	2.944580000	0.000424000	0.020645000
1	-2.964815000	0.000423000	-0.020687000

Zero-Point Corrected Electronic Energy:
-1856.274679 Hartrees

Vibrational Frequencies:

Frequency (cm^{-1})	Intensity (km/mol)
134.2923	3.1647
212.3076	59.0982
328.5563	2.18
447.0424	14.4539
574.3895	32.7165
669.5831	194.5481
703.1984	0.4245
3189.3096	131.0358
3211.149	113.9807

Electronic Transitions:

Wavelength (nm)	Oscillator Strength
29579.53	0
2188.2	0
959.97	0.072
677	0
396.39	0.0506
334.94	0.0008
322.83	0

296.45	0.0004
285.64	0.0595
258.69	0
246.54	0.026
240.79	0.0038
232.79	0.0022
223.19	0.0202
221.72	0.0006
213.9	0.001
193.25	0.0108
189.21	0
185.55	0.0039
184.22	0
177.41	0
169.28	0.0038
165.83	0.0207
161.98	0.0047
154.33	0
151.43	0.0065
148.77	0.0001
147.62	0
147.56	0
147.55	0
145.65	0.005
145.37	0.0005
144.53	0.0609
142.74	0.0061
140.63	0.0226
138.1	0.0001
138.09	0.0008
137.61	0
136.82	0.0106
136.7	0.0039
136.58	0.0278
134.73	0.0181
133.74	0.0176
133.62	0.0006
130.23	0.037
127.33	0.0001
126.55	0.0004
126.37	0.002
124.99	0
123.21	0.338

123.08	0
122.73	0.0002
122.64	0
122.43	0
121.95	0.0012
120.97	0.0073
120.95	0.3138
120.26	0.0011
120.16	0
119.24	0.0688
118.78	0
118.73	0.0002
118.6	0.004
117.68	0.0046
117.66	0.0051
117.31	0.0513
117.3	0.0312
117.24	0
116.76	0.2598
116.29	0.0521
116.29	0.0228
115.34	0.0013
115.27	0.0011
114.86	0
114.56	0.0031
114.29	0.0688
113.87	0
113.07	0.0082
113.03	0.0044
112.76	0.031
111.95	0.0093
111.41	0.0303
111.38	0.0896
111.29	0.0518
110.8	0
110.41	0.0068
109.65	0.0053
107.49	0
106.95	0.0014
106.04	0.0092
105.79	0.0147
105.36	0.0073
105.35	0.1017

105.31	0.1049
103.23	0.0033
102.34	0.0171
101.44	0.0003
101.14	0
100.85	0.0391
99.84	0.0004

$Zn^{+}(C_2H_2)$ Isomer 4
B3LYP
m=4

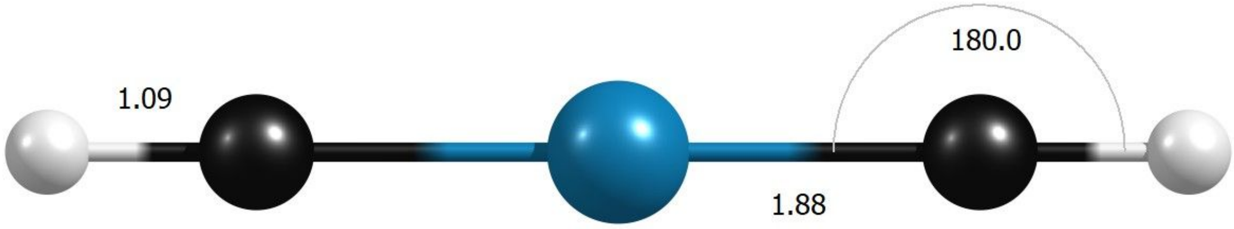

Coordinates:

30	0.000001000	-0.000030000	-0.000008000
6	-0.080433000	0.377669000	-1.840338000
6	0.080425000	-0.377569000	1.840366000
1	-0.126862000	0.595851000	-2.903130000
1	0.126882000	-0.595557000	2.903199000

Zero-Point Corrected Electronic Energy:
-1856.279342 Hartrees

Vibrational Frequencies:

Frequency (cm^{-1})	Intensity (km/mol)
40.2641	0.0005
129.1692	2.8345
176.3771	69.9125
509.4337	20.379
586.0279	0
645.1739	206.9174
691.2035	0.0008
3199.7415	235.162
3200.6972	0.0173

Electronic Transitions:

Wavelength (nm)	Oscillator Strength
90774.44	0
1557.19	0
859.67	0.103
328.46	0.0013
298.52	0
254.47	0
250.58	0.026

250.29	0
237.99	0.0016
225.72	0
222.82	0.0246
205.49	0.0058
200.17	0.0059
192.47	0
187.39	0
187.12	0
183.06	0.0068
180.24	0
176.07	0.0707
171.12	0
164.9	0
161.26	0
150.32	0
150.31	0
148.62	0
148.44	0
147.93	0
146.72	0
143.46	0.0655
143.13	0.0039
142.76	0.045
138.51	0
137.01	0
135.3	0
134.15	0.0097
131.66	0
131.18	0.0057
130.55	0.0011
130.49	0.0014
130.34	0.002
130.11	0.0588
126.56	0
125.75	0.0714
125.42	0
124.8	0
124.4	0.0055
124.18	0
124.07	0.0001
123.69	0
123.57	0.0047

122.47	0
121.45	0.0106
120.82	0.6927
120.72	0
120.15	0
119.73	0.4629
118.62	0
118.48	0.0056
118.18	0
117.51	0
117.08	0
117.07	0
116.58	0.0402
116.44	0.0385
116.09	0.0137
115.8	0
115.36	0
115.1	0
114.7	0.0004
114.22	0
114.02	0.0273
113.89	0
113.41	0
112.74	0
112.16	0
112.12	0
112	0
110.75	0.038
110.45	0
110.31	0
110.26	0
110.18	0
109.95	0
108.35	0.0792
107.54	0
107.27	0.0419
107.2	0
106.87	0.016
106.21	0.0791
105.09	0
103.2	0
102.69	0.0021
102.66	0

101.15	0.3915
101.03	0.0742
100.23	0
99.35	0
99.34	0
99.27	0.0432
99.05	0.0611

$Zn^{+}(C_2H_2)$ Isomer 1
M06
m=2

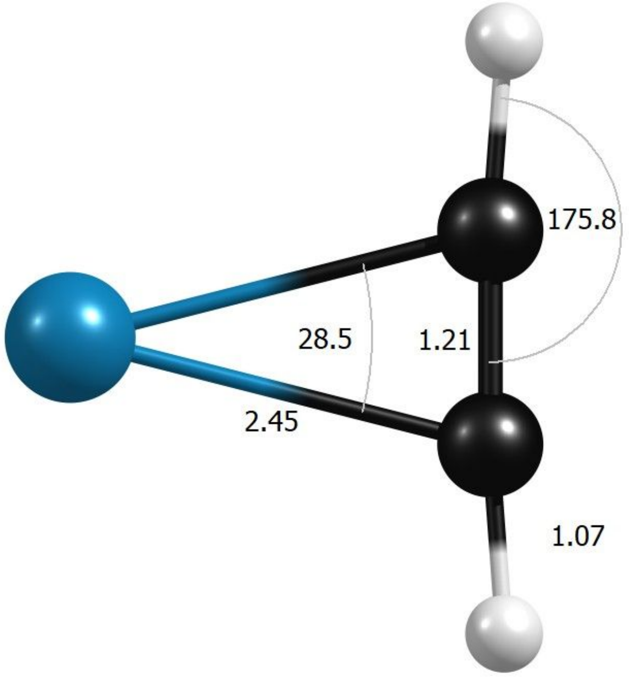

Coordinates:

30	0.759208000	0.000014000	0.000000000
6	-1.615582000	-0.604098000	0.000004000
6	-1.615735000	0.604038000	-0.000003000
1	-1.694029000	-1.674254000	-0.000010000
1	-1.694317000	1.674180000	0.000004000

Zero-Point Corrected Electronic Energy:
-1856.329252 Hartrees

Vibrational Frequencies:

Frequency (cm^{-1})	Intensity (km/mol)
40.1219	6.3146
214.676	13.6398
659.6984	0
711.8632	4.7639
793.9316	89.5472
870.0577	112.9361
2008.7705	75.9132
3307.3988	248.1469

3413.3674	0.5996

Electronic Transitions:

Wavelength (nm)	Oscillator Strength
350.41	0.0012
347.48	0.0159
268.95	0.0835
243.96	0
241.47	0.1013
227.35	0
225.06	0.0084
216.02	0
206.24	0.024
189.17	0.0283
186.43	0.3764
175.9	0.0023
169.06	0.0032
163.66	0.0445
163.3	0.0055
160.36	0
151.49	0.0375
150.28	0.0017
150.22	0.0007
149.96	0.0136
149.67	0
149.42	0.0365
148.85	0
146.35	0
144.37	0.0013
143.57	0.002
142.08	0.0021
141.3	0.0115
140.28	0
140.1	0.0015
136.82	0.114
135.9	0.27
134.87	0.0088
132.03	0.048
130.06	0.0601
129.72	0
129.3	0
128.12	0.0783

128.06	0.0721
126.11	0.0002
125.06	0
123.38	0.008
122.92	0.009
122.9	0
121.09	0
119.47	0.0811
118.45	0.006
118.43	0.014
117.72	0.0246
115.89	0
115.69	0.0129
115.01	0.0136
114.69	0.0672
112.66	0.0019
112.47	0.0033
111.25	0.0133
110.26	0.0077
109.09	0.0119
107.92	0.0003
107.92	0.0393
107.05	0.0039
106.48	0
106.47	0.0058
106.31	0.0661
106.18	0.0008
106.01	0.0017
105.95	0.0016
105.7	0
105.69	0.0293
105.25	0.0052
104.88	0
104.54	0.0158
104	0
103.89	0.0162
103.71	0.0191
103.45	0.0826
103.42	0.0624
103.2	0.065
102.88	0.0151
101.43	0
100.85	0.0154

100.33	0.0018
99.8	0
99.71	0
99.61	0
99.26	0.0023
99.23	0.0055
99.11	0
99.06	0.0034
98.92	0
98.33	0.0177
98.01	0.0034
97.85	0.0086
97.74	0.0019
97.73	0.0013
97.09	0.0128
96.52	0.0012
96.4	0
96.32	0.0078
96.06	0.0018

$Zn^+(C_2H_2)$ Isomer 2
M06
m=4

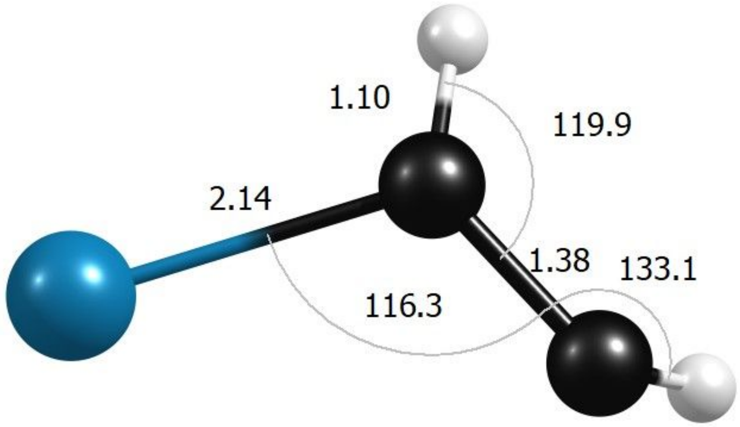

Coordinates:

30	0.823350000	-0.054998000	0.010099000
6	-1.222191000	0.568035000	-0.132500000
6	-2.168383000	-0.431793000	-0.090379000
1	-1.385323000	1.500319000	0.426556000
1	-2.971724000	-0.667837000	0.607738000

Zero-Point Corrected Electronic Energy:
-1856.206329 Hartrees

Vibrational Frequencies:

Frequency (cm^{-1})	Intensity (km/mol)
172.9664	3.7289
325.9983	6.2465
411.929	184.8343
831.4432	18.2829
951.0087	11.1216
1032.2532	40.9367
1368.1386	6.7717
3015.4941	16.6442
3111.5547	40.4519

Electronic Transitions:

Wavelength (nm)	Oscillator Strength
596.78	0.0167
528.72	0.0123

382.01	0.0346
280.01	0.0525
273.19	0.0533
249.85	0.1753
234.51	0.0007
224.49	0.0197
213.83	0.0043
205.07	0.0709
203.75	0.0678
201.93	0.0657
185.97	0.0289
180.53	0.146
171.36	0.0136
169.01	0.0505
168.28	0.0013
164.86	0.0141
162.67	0.0102
155.88	0.02
154.68	0.0354
153.26	0.019
149.42	0.0027
149.02	0.003
148.76	0.0012
148.7	0.0004
148.13	0.0127
147.41	0.01
146.77	0.0145
145.5	0.0122
144.19	0.0071
143.89	0.0332
142.17	0.0203
141.42	0.0098
141.23	0.0084
140.41	0.0068
139.68	0.0104
139.29	0.0045
138.5	0.0214
138.43	0.0006
138.26	0.0159
137.81	0.0131
135.15	0.008
134.94	0.0284
133.53	0.0071

132.67	0.1093
132.34	0.1043
130.31	0.098
128.52	0.0045
127.63	0.0006
125.4	0.0145
123.9	0.0281
123.58	0.0084
122.67	0.0006
122.35	0.0182
121.61	0.036
121.19	0.0056
120.33	0.0344
118.25	0.0105
117.47	0.0092
117.17	0.0216
115.31	0.0211
114.78	0.0239
113.36	0.0045
112.93	0.0136
112.7	0.0333
112.6	0.0015
112.33	0.0231
112.02	0.0081
111.96	0.0141
111.58	0.0205
110.8	0.0066
110.73	0.0046
109.94	0.0133
108.94	0.0186
108.72	0.0359
107.89	0.0138
107.32	0.0079
107.26	0.0018
106.92	0.0049
106.86	0.0023
106.66	0.003
106.31	0.0211
105.34	0.0098
104.69	0.0023
104.39	0.0117
103.82	0.0146
103.55	0.0048

102.98	0.0008
102.84	0.012
102.63	0.0065
102.02	0.0405
101.41	0.0263
101.15	0.0009
100.89	0.0032
100.63	0.018
100.52	0.0012
100.23	0.0047
100.11	0.0086
99.84	0.0202

$Zn^+(C_2H_2)$ Isomer 3
M06
m=2

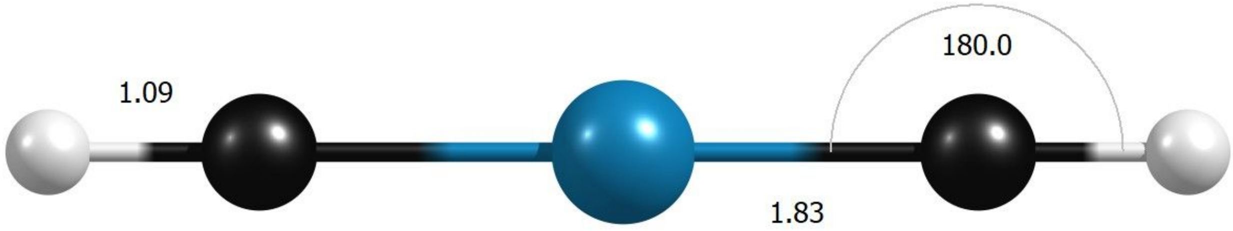

Coordinates:

30	0.000000000	0.000000000	0.007454000
6	0.000000000	0.000000000	1.840622000
6	0.000000000	0.000000000	-1.871327000
1	0.000000000	0.000000000	2.922345000
1	0.000000000	0.000000000	-2.961723000

Zero-Point Corrected Electronic Energy:
-1856.114947 Hartrees

Vibrational Frequencies:

Frequency (cm^{-1})	Intensity (km/mol)
135.838	4.1919
148.2478	2.3799
191.9315	53.9605
329.5236	0.8589
485.6711	17.2341
576.2999	194.3872
697.1756	320.9654
720.1024	1.5807
3142.2889	118.4292
3263.7589	102.8649

Electronic Transitions:

Wavelength (nm)	Oscillator Strength
25329.81	0
3060.62	0
1178.53	0.0606
874.27	0
445.83	0.0755
330.72	0.0006

288.96	0
264.05	0
252.79	0.0008
251.23	0.0269
248.45	0.0549
244.67	0.0019
240.57	0.0008
230.24	0.0187
213.94	0.0004
212.93	0.0027
195.42	0
191.85	0.0122
191.48	0
188.77	0
187.13	0.0048
172.71	0.0192
162.1	0.0065
158.04	0.0299
155.32	0.0236
153.76	0.0051
150.07	0
147.88	0.0067
146.95	0.0032
146.64	0.0024
144.91	0.0052
144.54	0.0006
143.78	0
142.48	0.0149
141.71	0.0163
140.92	0
140.68	0
140.16	0
140.15	0
140.06	0
139.51	0
138.95	0.0004
137.19	0.0062
136.91	0.0177
135.02	0.0002
134.98	0.0002
134.82	0
134.39	0.0168
133.65	0.0005

133	0.0005
129.47	0.0688
129.17	0.0022
128.06	0.0004
123.92	0.4504
123.16	0.003
122.98	0.0006
121.95	0.0025
121.88	0.0056
121.64	0
121.47	0.0496
121.12	0
121.08	0.0017
120.89	0.0027
119.97	0
119.63	0.0018
119.45	0.0724
119.39	0.0744
119.22	0.0523
118.57	0.0007
117.56	0.002
117.22	0.0128
116.74	0
116.39	0.0513
114.95	0.3564
114.73	0.01
113.93	0.085
112.16	0.0136
111.83	0.0022
111.22	0.0461
111.08	0.0046
110.08	0.0325
108.55	0.0746
107.73	0.0043
107.06	0.0001
107.04	0
106.82	0
106.53	0.0027
106.49	0.0001
106.48	0
106.43	0.0036
106.33	0.0148
106.09	0.0029

105.31	0
105.25	0.0036
104.89	0.0282
104.52	0.0398
104.43	0.0626
103.74	0.0081
102.73	0.2884
102	0.1922

$Zn^+(C_2H_2)$ Isomer 4
M06
m=4

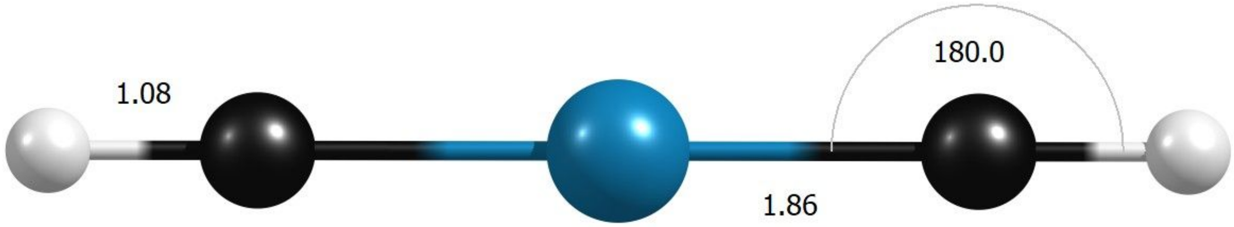

Coordinates:

30	-0.000002000	0.000067000	0.000011000
6	1.864734000	-0.000201000	-0.000027000
6	-1.864725000	0.000135000	-0.000032000
1	2.948067000	-0.000170000	0.000005000
1	-2.948062000	-0.001445000	0.000020000

Zero-Point Corrected Electronic Energy:
-1856.126203 Hartrees

Vibrational Frequencies:

Frequency (cm^{-1})	Intensity (km/mol)
131.3961	0.0016
139.6254	1.4454
220.4953	64.0127
361.3364	0.8083
602.9458	11.3246
619.4749	0
678.1085	255.4728
3243.8797	207.7086
3244.4789	0.309

Electronic Transitions:

Wavelength (nm)	Oscillator Strength
73062.41	0
1939.65	0
860.08	0.1048
307.46	0.0005
273.58	0
250.78	0.0229
244.8	0

244.15	0
226.24	0.007
225.17	0
215.53	0.0302
204.93	0.0037
198.28	0
190.19	0
187.55	0
180.89	0.0731
179.31	0.0098
170.34	0
170.11	0.0088
165.31	0
164.12	0
160.01	0
159.06	0.0307
157.21	0
156.52	0.0313
156.47	0
146.35	0
144.6	0
144.59	0
143.78	0
143.76	0
143.74	0.0269
143.61	0
143.44	0.0001
142.46	0
141.64	0
139.15	0
138.54	0.0296
138.04	0
137.31	0.0286
133.52	0.0031
132.28	0
131.69	0
130.51	0
129.26	0.0092
126.91	0
124.94	0.0017
123.11	0
121.97	0.4022
121.53	0

121.38	0.003
121.37	0
120.51	0.0012
120.43	0.0006
120.39	0
119.62	0
119.4	0
119.38	0.2196
118.64	0.075
118.53	0
117.64	0.0216
117.61	0.0834
117.57	0
116.84	0.0017
116.82	0.0017
116.53	0
116.06	0
115.65	0
115.37	0.4048
115.17	0
114.6	0.0028
114.59	0
113.45	0
112.86	0
112.28	0
112.08	0.0638
111.6	0.0345
109.54	0.0243
109.18	0.057
108.69	0.0177
106.78	0.0963
106.72	0.0001
106.69	0
105.54	0
105.53	0
104.93	0
104.57	0
104.28	0
104.04	0
103.06	0
102.72	0
102.11	0.3147
101.94	0

101.7	0
101.66	0
101.55	0
101.33	0.3972
100.83	0.0099
100.63	0
100.46	0

$Zn^+(C_2H_2)$ Isomer 1

M06-L

m=2

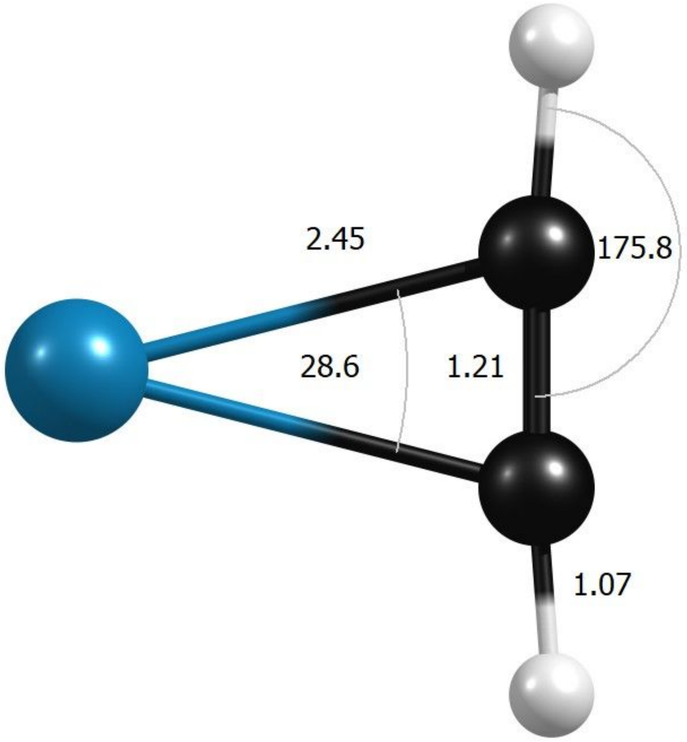

Coordinates:

30	0.758043000	-0.000005000	0.000000000
6	-1.613215000	0.604812000	0.000001000
6	-1.613269000	-0.604791000	0.000000000
1	-1.691144000	1.672861000	-0.000002000
1	-1.691250000	-1.672834000	0.000000000

Zero-Point Corrected Electronic Energy:

-1856.363057 Hartrees

Vibrational Frequencies:

Frequency (cm^{-1})	Intensity (km/mol)
21.5965	6.7087
214.183	14.393
622.8565	0
687.4769	3.8047
804.1444	85.9759
876.3238	109.9309
2007.4073	62.898

3332.1437	242.9165
3436.8508	1.5136

Electronic Transitions:

Wavelength (nm)	Oscillator Strength
362.46	0.0239
343.73	0.0008
260.27	0.0731
249.1	0
248.97	0.1313
221.16	0.017
220.65	0
208.8	0.0306
202.38	0
192.96	0.0306
183.73	0.3749
170	0.0214
164.23	0.0258
162.98	0
162.93	0.0024
161.47	0.0001
161.44	0.0002
160.17	0.0627
159.15	0.0023
153	0
152.2	0.1153
149.77	0.0001
146.06	0.0004
144.07	0.0002
143.61	0
139.4	0
137.14	0.1215
135.47	0.0159
135.39	0
133.71	0
132.67	0.3437
130.46	0.0001
130.06	0
129.31	0.0279
126.25	0.0083
125.72	0.0013
125.67	0

123.83	0
120.13	0.0012
119.56	0.0129
119.32	0.0002
119.31	0.0087
119.07	0.0002
118.97	0
118.87	0.0002
118.66	0
117.67	0.0075
117.47	0.0022
117.21	0
116.85	0
116.61	0.0248
116.57	0.0008
116.47	0
115.95	0.0082
115.72	0.0201
114.82	0.0048
114.56	0.0171
113.93	0.2031
113.48	0.0032
113.16	0.051
111.62	0.0153
110.32	0.0942
107.64	0.0039
105.98	0.0005
105.79	0.0016
105.71	0.001
105.13	0.0036
105.1	0.0063
104.82	0
104.39	0.0001
104.38	0
103.49	0.0048
103.39	0.0011
103.32	0.0231
103.2	0.0015
103.08	0
102.75	0
102.72	0.0019
102.71	0
102.63	0.0419

102.44	0.0026
102.15	0.0277
102.13	0.0129
101.89	0
101.84	0.016
101.66	0.006
101.59	0
101.58	0.1203
101.43	0.0554
101.08	0
100.94	0
100.27	0.0289
100.13	0.0016
100.01	0.0808
99.68	0.0015
98.92	0.0778
97.51	0.0012
97.38	0
96.93	0.0006
96.37	0.0002

$Zn^+(C_2H_2)$ Isomer 2
M06-L
m=4

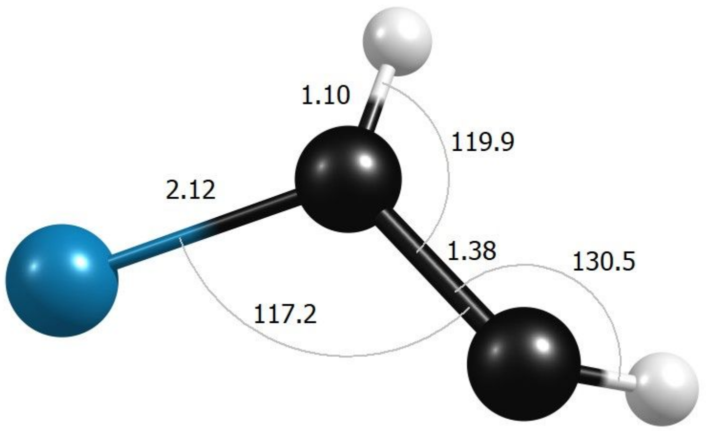

Coordinates:

30	0.817538000	-0.055715000	0.010219000
6	-1.206768000	0.563649000	-0.139387000
6	-2.173370000	-0.421972000	-0.098894000
1	-1.331352000	1.477622000	0.456972000
1	-2.913969000	-0.656239000	0.666135000

Zero-Point Corrected Electronic Energy:
-1856.244922 Hartrees

Vibrational Frequencies:

Frequency (cm^{-1})	Intensity (km/mol)
187.3878	3.2922
336.8462	3.2638
448.2327	162.9751
848.3483	21.4035
941.4369	21.2034
1053.5161	33.1682
1339.5024	22.973
3029.5448	18.7673
3111.6676	36.7829

Electronic Transitions:

Wavelength (nm)	Oscillator Strength
535.26	0.0382
424.32	0.0251

375.15	0.0352
269.52	0.0681
266.27	0.0768
244.14	0.1934
221.14	0.0106
216.17	0.007
213.13	0.0196
205.94	0.0373
201.2	0.0501
185.55	0.0521
182.31	0.0226
179.86	0.0051
179.43	0.0007
179.32	0.0013
178.6	0.0071
176.47	0.1101
166.34	0.0297
163.99	0.0601
157.48	0.031
155.64	0.0292
153.95	0.0215
153.48	0.0223
152.93	0.0028
152.55	0.006
152.17	0.0128
150.98	0.0042
150.16	0.0267
148.18	0.0167
147.41	0.0258
143.53	0.0144
142.36	0.0119
141.2	0.0223
140.18	0.013
138.59	0.0039
137.57	0.0367
136.38	0.0121
135.5	0.0223
133.75	0.0411
133.05	0.0609
132.57	0.0105
132.33	0.0034
132.27	0.0019
131.95	0.0157

131.66	0.0724
130.87	0.1006
129.17	0.009
129	0.0172
125.91	0.0066
125.36	0.0216
125.14	0.0104
124.77	0.0106
124.62	0.022
124.4	0.0042
124.34	0.0073
124.23	0.0087
122.88	0.01
121.45	0.1167
119.61	0.0186
118.47	0.0088
117.45	0.0229
116.38	0.013
115.51	0.0126
114.59	0.0305
113.26	0.0113
112.95	0.0071
110.58	0.0101
110.36	0.001
110.07	0.0109
109.94	0.0192
109.73	0.0008
109.61	0.01
109.31	0.0084
109.15	0.005
108.75	0.0131
108.37	0.0024
108.09	0.0311
107.62	0.0075
107.43	0.0083
106.26	0.0768
105.93	0.0017
105.75	0.0033
105.59	0.0018
105.23	0.0065
104.86	0.0023
104.66	0.0047
104.54	0.0131

104	0.008
103.92	0.0061
103.63	0.0002
103.44	0.0067
103.4	0.003
103.17	0.0141
102.8	0.0029
102.62	0.0328
102.41	0.0162
102.29	0.0305
102.13	0.0079
101.95	0.0271

$Zn^+(C_2H_2)$ Isomer 3
M06-L
m=2

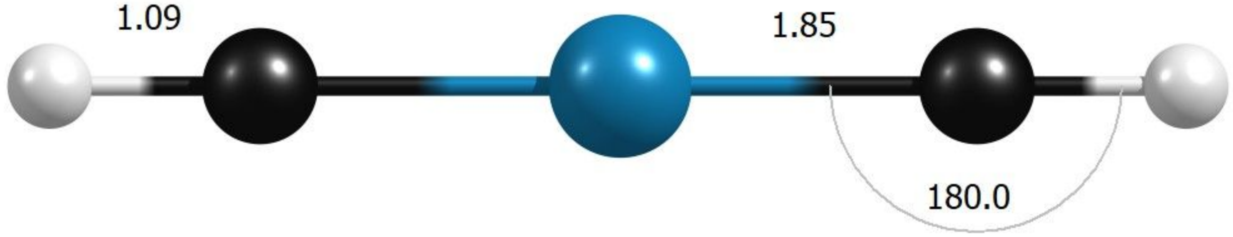

Coordinates:

30	0.003071000	0.000023000	0.000006000
6	1.850214000	0.000028000	0.009546000
6	-1.862575000	-0.000027000	-0.009562000
1	2.932746000	-0.000463000	0.015056000
1	-2.950708000	-0.000245000	-0.015143000

Zero-Point Corrected Electronic Energy:
-1856.162602 Hartrees

Vibrational Frequencies:

Frequency (cm^{-1})	Intensity (km/mol)
149.8636	0.9728
172.1461	44.7612
322.315	1.8978
352.1827	3.5326
584.61	11.8375
691.1125	115.7953
696.3704	4.0895
3185.2263	111.2937
3254.7785	77.2226

Electronic Transitions:

Wavelength (nm)	Oscillator Strength
9530.82	0
1688.55	0
887.57	0.0569
544.85	0
433.11	0.0515
352.92	0
316.15	0.0006

308.38	0.0786
287.73	0.0007
260.98	0.0006
233.92	0.0143
220.97	0.0003
218.74	0.017
209.59	0.0267
206.06	0.0002
198.58	0.0007
190.74	0.0026
188.55	0.0119
172.84	0.0036
171.52	0
171.2	0
167.62	0
167.6	0
166.27	0
165.94	0.0053
162.46	0
162.39	0.001
158.34	0.026
158.1	0.0004
152.6	0
149.92	0
149.81	0.0007
149.69	0.0007
148.42	0
145.92	0.0162
145.2	0.0017
139.04	0.0077
137.16	0.0034
136.98	0.0257
135.97	0.0075
135.01	0.0319
133.25	0.0007
133.21	0.0004
131.98	0.0032
131.23	0
130.42	0.0347
130.07	0.0194
129.75	0.0066
129.18	0
128.84	0.0246

125.91	0
125.43	0.0131
124.3	0
124.26	0.0005
123.97	0.0026
123.8	0.0018
123.58	0.0009
123.37	0
122.38	0.0014
122.17	0.0017
121.83	0.0305
121.77	0
121.44	0.0003
121.38	0.0003
119.99	0
119.68	0.2277
118.33	0.013
118.26	0.0035
117.81	0.001
117.26	0.0001
116.33	0.0052
115.61	0.0127
115.09	0.5643
114.94	0.0041
114.44	0.4657
113.8	0.0024
112.53	0
111.54	0.0139
111.27	0.003
110.8	0
110.05	0.0432
108.6	0.0107
106.58	0.0014
106.22	0.0164
106.08	0.0565
105.29	0.0003
105.21	0.0009
104.49	0.025
104.15	0.0019
104.07	0
103.44	0.0043
102.92	0.0052
102.86	0.0013

101.43	0
101.12	0
100.38	0.5057
100.23	0.0597
99.4	0.0113
98.99	0.0002
98.96	0

$Zn^+(C_2H_2)$ Isomer 4
M06-L
m=4

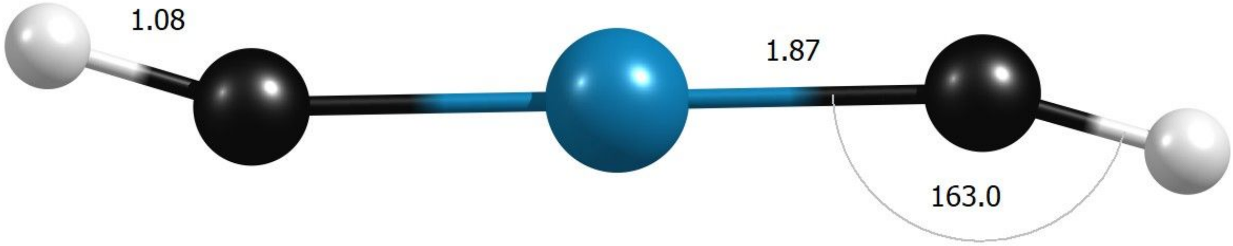

Coordinates:

30	-0.000024000	-0.000821000	-0.003072000
6	-1.874227000	-0.058900000	0.004974000
6	1.873801000	0.060465000	0.004663000
1	-2.917161000	0.237541000	0.016578000
1	2.920435000	-0.222296000	0.017759000

Zero-Point Corrected Electronic Energy:
-1856.170426 Hartrees

Vibrational Frequencies:

Frequency (cm^{-1})	Intensity (km/mol)
141.5744	2.6027
148.9079	2.4125
231.779	10.3725
248.292	46.6403
526.9318	18.0967
589.9595	0.0016
664.451	101.8103
3220.9033	76.8
3225.0749	59.8298

Electronic Transitions:

Wavelength (nm)	Oscillator Strength
4194.24	0
1384.97	0
755.82	0.0982
308.97	0.0012
294.04	0
246.67	0
234.47	0

224	0.0348
214.45	0.0003
211.41	0
208.42	0.0392
200.26	0.0043
196.14	0.0149
186.48	0.0061
183.42	0
172.4	0
172.37	0.0003
172.32	0.0028
172	0.0001
171.44	0.0001
170.06	0
169.61	0.0055
169.48	0.0916
166.64	0
160.6	0
160.09	0
157.01	0
141.04	0.0027
140.68	0.0001
140.18	0.0323
138.59	0.001
138.44	0.0014
138.42	0.0005
138.18	0.0004
137.05	0.005
136.19	0.0424
135.85	0.0049
132.14	0.0032
131.72	0
131.53	0.0417
131.36	0
131.16	0
131.09	0.0017
131.01	0.0009
129.85	0.0183
129.72	0
128.65	0.0004
128.53	0.0311
127.22	0.0001
124.84	0

123.93	0
123.77	0.0003
123.32	0
122.77	0
122.77	0
121.92	0
121.45	0
121.39	0.0017
120.85	0
119.21	0.0002
119.03	0.0007
118.75	0
117.74	0.0859
117.07	0.0005
117.04	0.0002
116.95	0.6146
116.1	0.0005
115.67	0.0003
115.48	0.5328
114.6	0.0004
114.27	0.0033
113.63	0
112.79	0.047
112.62	0.001
112.39	0.0017
111.94	0.0026
110.67	0
110.49	0.0132
108.98	0
108.53	0.119
105.57	0.1142
105.04	0
104.78	0
104.73	0.0001
104.01	0.0001
103.92	0.0142
103.28	0.0018
102.94	0.0201
102.89	0.0816
102.54	0
101.82	0.0126
100.75	0.0008
100.13	0.0001

100.13	0
100.1	0
99.85	0.0076
99.4	0
99.35	0
98.97	0.0014
98.68	0

$Zn^+(C_2H_2)$ Isomer 1
MN15-L
m=2

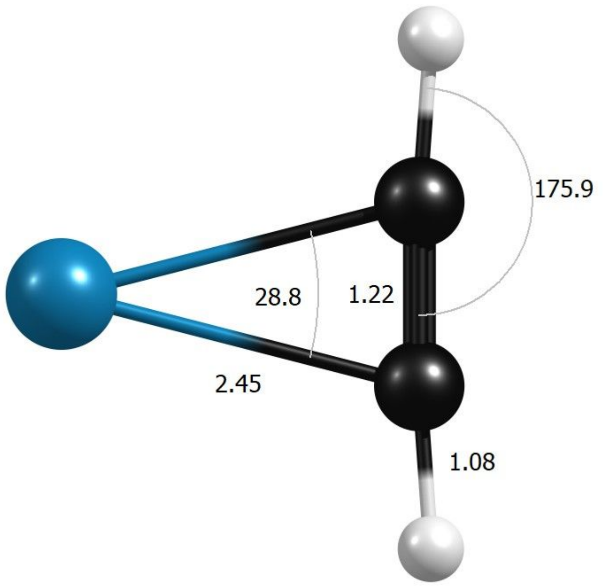

Coordinates:

30	0.758957000	0.000002000	0.000000000
6	-1.615373000	0.609308000	-0.000003000
6	-1.615358000	-0.609315000	0.000001000
1	-1.692178000	1.688263000	0.000009000
1	-1.692145000	-1.688272000	0.000001000

Zero-Point Corrected Electronic Energy:
-1856.349166 Hartrees

Vibrational Frequencies:

Frequency (cm^{-1})	Intensity (km/mol)
108.7748	7.7631
209.5782	17.0047
586.3774	0
662.8024	2.1873
809.557	90.1083
882.6558	130.951
2014.4124	72.3765
3335.4185	285.9412
3442.1724	2.3054

Electronic Transitions:

Wavelength (nm)	Oscillator Strength
379.78	0.0405
303.99	0.0007
272.68	0.1463
243.43	0.0698
233.3	0
225.98	0.0211
212.9	0
199.76	0
198.82	0.0547
189.3	0.0015
187.75	0.3859
169.98	0.0239
169.17	0.1063
157.82	0.0017
155.27	0.0179
153.24	0.0056
151.8	0.0287
150.61	0.0009
150.25	0
150.16	0.0001
149.9	0.0025
149.18	0.0789
147.42	0
142.82	0
138.16	0.0892
137.6	0
136.39	0.0476
136.33	0
133.7	0.3435
133.56	0
132.86	0.0001
132.22	0.0005
130.23	0.0252
130.08	0.0087
127.3	0.0031
126.82	0.0555
126.37	0.009
122.84	0.0461
121.58	0
120.95	0
119.16	0.0038
119.06	0

119	0.0153
118.43	0
118.31	0
118.25	0
117.86	0.0293
116.75	0
116.5	0.0316
115.47	0.0229
115.41	0.0039
115.28	0.0005
115.06	0
114.96	0.0104
114.25	0.0075
113.72	0.0005
113.42	0.1493
112.77	0.0009
112.41	0.1463
110.53	0.0461
108.74	0.0166
108.11	0.0019
107.92	0.0005
107.64	0.0076
107.49	0.0047
107.09	0.0247
106.88	0.0068
106.14	0
106.06	0.0003
105.26	0.0357
104.51	0.0034
104.48	0.0436
103.96	0.0063
102.67	0.0123
101.95	0.1232
101.81	0
100.86	0.0432
100.45	0.043
100.42	0.0226
100.37	0
100.18	0.0016
100.13	0.0055
100.11	0.0003
99.97	0.0504
99.83	0

99.67	0.0018
99.64	0
99.54	0.003
99.46	0
99.35	0.0015
99.31	0.0006
99.2	0
99	0.0002
98.99	0.0071
98.75	0
98.54	0.0031
98.32	0
98.31	0.0295
97.95	0
97.62	0.0012

$Zn^+(C_2H_2)$ Isomer 2
MN15-L
m=4

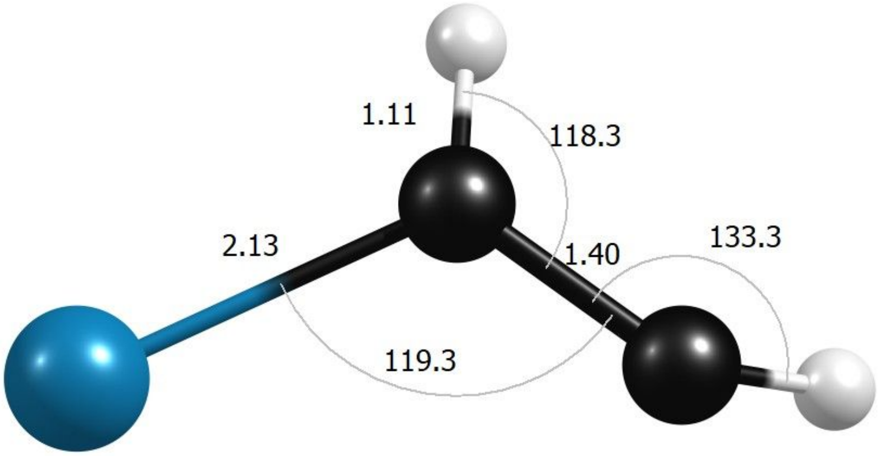

Coordinates:

30	-0.831650000	0.055108000	0.010487000
6	1.202515000	-0.544795000	-0.123390000
6	2.207810000	0.427675000	-0.081706000
1	1.402522000	-1.529059000	0.351554000
1	3.085019000	0.578552000	0.564406000

Zero-Point Corrected Electronic Energy:
-1856.226753 Hartrees

Vibrational Frequencies:

Frequency (cm^{-1})	Intensity (km/mol)
172.3742	5.3973
332.7024	52.9539
379.3787	154.2671
848.301	19.8009
942.4034	14.3711
1066.928	26.7956
1329.9789	12.6001
3007.1611	11.0672
3136.6568	42.7582

Electronic Transitions:

Wavelength (nm)	Oscillator Strength
568.7	0.0458

457.36	0.012
363.38	0.0283
300.86	0.1004
280.58	0.1019
233	0.1063
217.56	0.0199
215.7	0.0269
212.13	0.0162
205.97	0.029
192.24	0.0748
190.28	0.1212
181.92	0.0045
180.85	0.0146
180.49	0
179.92	0.0084
179.08	0.006
177.58	0.0616
170.27	0.1749
157.52	0.0322
156.93	0.0209
155.7	0.0401
153.49	0.0208
151.49	0.0063
150.9	0.0217
148.36	0.0203
146.18	0.0105
145.58	0.0013
144.3	0.0002
144.12	0.0008
143.86	0.0034
142.5	0.009
141.91	0.0223
141.76	0.0052
140.22	0.0387
136.43	0.0001
136.11	0.0283
135.35	0.0013
135.05	0.0299
134.15	0.0005
133.54	0.033
132.76	0.0149
132.46	0.0077
132.15	0.0009

132.06	0.0009
131.19	0.0742
130.37	0.072
129.5	0.114
128.91	0.0527
127.44	0.0169
126.26	0.0052
124.64	0.0197
123.17	0.0014
122.36	0.004
122.21	0.0058
121.85	0.0566
121.59	0.0153
120.3	0.013
119.47	0.0371
119.14	0.0088
116.99	0.0286
116.76	0.015
115.52	0.0134
114.77	0.0282
114.02	0.0169
113.31	0.0179
112.9	0.045
112.12	0.0062
111.4	0.0132
111.02	0.0112
109.41	0.0142
109.3	0.0033
109.15	0.0264
108.98	0.0065
108.48	0.0019
108.39	0.002
108.19	0.0012
108.12	0.0019
107.71	0.0066
107.38	0.0295
107.18	0.0028
106.65	0.0068
106.55	0.0029
106.23	0.0043
106.05	0.0235
105.72	0.0169
105.56	0.0344

105.44	0.0288
105.09	0.0079
104.85	0.0238
104.73	0.0303
104.45	0.004
103.59	0.0048
103.34	0.021
103.12	0.016
102.88	0.0051
102.63	0.0237
102.29	0.034
101.98	0.0469
101.84	0.0107

$Zn^+(C_2H_2)$ Isomer 3
MN15-L
m=2

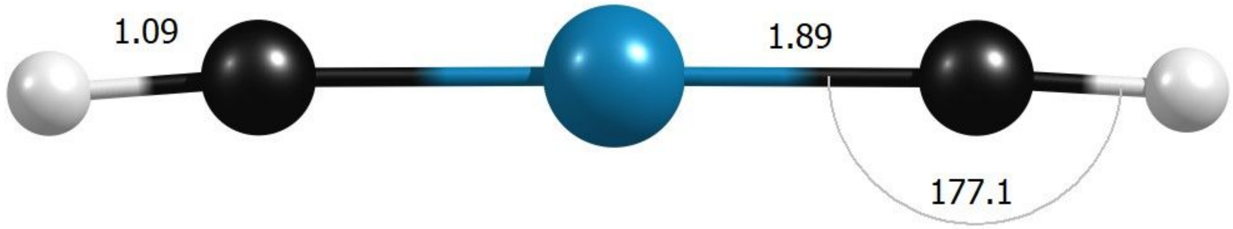

Coordinates:

30	0.005703000	-0.006122000	-0.000021000
6	1.859126000	0.000909000	0.000065000
6	-1.882788000	0.004669000	0.000050000
1	2.950344000	0.082827000	-0.000061000
1	-2.979468000	0.067364000	-0.000008000

Zero-Point Corrected Electronic Energy:
-1856.145758 Hartrees

Vibrational Frequencies:

Frequency (cm^{-1})	Intensity (km/mol)
143.8761	2.7638
207.2971	35.3804
346.2532	6.4624
421.4229	1.2563
573.9884	25.9209
694.2725	100.5187
711.8192	0.5824
3195.623	175.0932
3222.1309	121.6911

Electronic Transitions:

Wavelength (nm)	Oscillator Strength
7489.8	0
1455.02	0
842.8	0.0561
565.76	0
430.49	0.0839
306.77	0
282.38	0.0813

279.12	0.0018
267.51	0.0042
252.97	0.0007
227.28	0.0117
223.77	0.0155
222.88	0.0362
204.93	0.0075
203.6	0.0022
201.24	0.0201
188.49	0.0051
186.19	0.0026
186	0.0187
182.94	0.0004
170.07	0.0002
167.95	0.022
160.66	0.0062
157.41	0.0051
156.89	0
156.83	0.0009
156.14	0
152.67	0.0027
149.85	0.0015
149.73	0.0001
147.95	0.0004
145.68	0.0026
144.89	0.0021
144.84	0.0003
143.8	0.0206
143.49	0.0002
143.1	0.0423
142.81	0.0087
140.45	0.0253
139.01	0.0338
138.68	0.0166
134.17	0.0284
131.28	0.0041
130.97	0.0174
130.05	0.0008
129.35	0.0007
129.3	0.0007
128.55	0.0003
127.73	0.0009
127.32	0.0003

127.16	0
126.14	0
125.62	0
124.46	0.0343
124.19	0
124.15	0.0023
122.79	0.2603
122.26	0.0123
120.67	0.0085
120.4	0
120.1	0.0059
119.74	0.0003
119.26	0.0001
119.25	0.0006
118.41	0
118.28	0.0005
118.26	0
118.1	0.0008
117.96	0
117.72	0.0164
117.4	0.001
117.06	0.0001
116.97	0
116.36	0.3508
116.25	0.046
116.2	0.0111
114.08	0.4096
113.84	0.102
112.84	0.0018
112.13	0.033
111.92	0.0313
111.67	0.1241
111.02	0.072
110.73	0.0006
109.99	0.0393
109.1	0.0161
109.06	0
107.41	0.036
107	0.036
106.7	0.008
105.81	0
104.92	0.0234
104.92	0.0402

104.35	0.0169
103.86	0.0079
103.4	0.0271
102.78	0.0096
102.03	0.08
101.39	0
99.93	0.228

$Zn^+(C_2H_2)$ Isomer 4
MN15-L
m=4

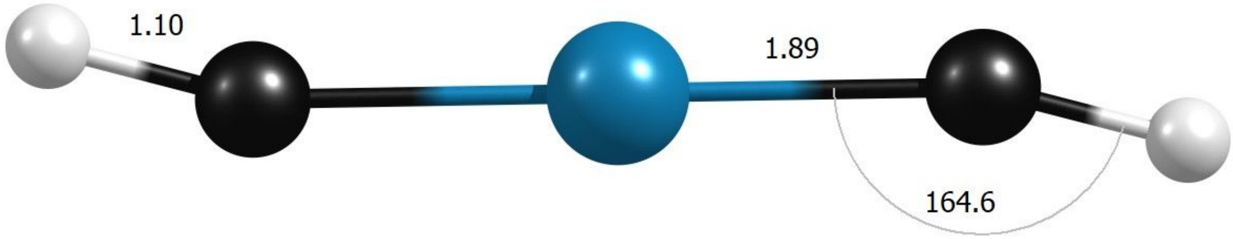

Coordinates:

30	-0.000001000	-0.000038000	0.000846000
6	-1.887941000	-0.053926000	-0.001366000
6	1.887921000	0.053981000	-0.001361000
1	-2.953625000	0.207319000	-0.004497000
1	2.953784000	-0.206512000	-0.004514000

Zero-Point Corrected Electronic Energy:
-1856.152300 Hartrees

Vibrational Frequencies:

Frequency (cm^{-1})	Intensity (km/mol)
125.7405	1.5932
139.5134	0.1959
222.1649	0.011
236.5337	53.0774
530.3255	14.0347
583.6383	0.0001
656.0908	127.4143
3195.8087	247.5705
3197.9562	0.0319

Electronic Transitions:

Wavelength (nm)	Oscillator Strength
4083.92	0
1226.86	0
746.97	0.1107
279.73	0.0032
276.81	0
239.86	0
232.46	0

224.83	0.0047
221.92	0.051
215.88	0
210.42	0.0333
201.98	0.0378
195.23	0.0031
183.19	0.0068
181.68	0
180.94	0
180.62	0
176.91	0.0032
173.64	0.0999
170.68	0
163.64	0
163.6	0
163.49	0
162.87	0
162.43	0
156.86	0
152.97	0
143.79	0.0578
142.98	0
139.69	0.0368
139.33	0
137.7	0.0014
136.73	0.0101
136.39	0.0015
136.29	0.0006
135.48	0
134.35	0
134.27	0.0179
134.19	0.0204
131.29	0.0189
130.12	0.0174
129.88	0.0148
129.71	0.0001
128	0
127.46	0.0164
127.46	0.0037
126.94	0
126.54	0.0035
125.9	0.07
124.23	0

123.94	0
123.71	0.0006
123.43	0
120.1	0.0164
119.86	0
119.65	0
119.23	0
119.22	0
118.45	0.9075
117.71	0
117.62	0.0002
117.25	0.0589
117.1	0
116.95	0
116.58	0.0001
116.23	0.0166
115.66	0.1118
115.59	0.0011
114.84	0
113.82	0
113.68	0
113.18	0.0356
113.15	0.0008
112.76	0.0105
112.63	0
112.6	0
111.67	0.0448
111.48	0
111.31	0
111.25	0
110.98	0
110.49	0.1465
110.38	0.0001
108.37	0
107.97	0.0006
107.51	0.0017
107.23	0.0096
107.06	0
106.62	0
105.9	0.1077
103.64	0.024
103.38	0
102.71	0.3678

101.25	0.138
101.22	0.0015
100.25	0
100.25	0
100.23	0.1067
99.47	0
99.1	0

$Zn^+(C_2H_4)$ Isomer 1
B3LYP
m=2

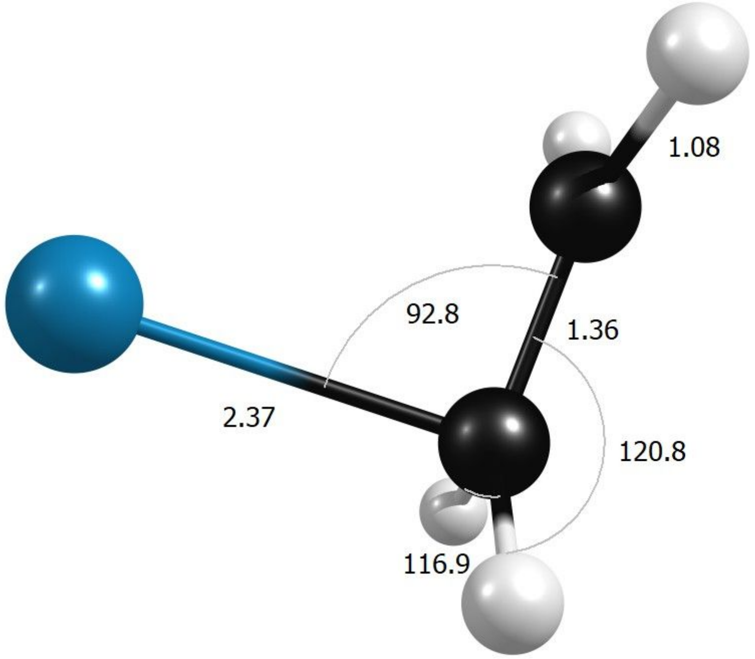

Coordinates:

30	0.871188000	-0.050852000	-0.000001000
6	-1.870247000	-0.538939000	0.000000000
6	-1.366092000	0.720031000	0.000003000
1	-2.089416000	-1.061208000	-0.924473000
1	-2.089394000	-1.061236000	0.924466000
1	-1.269410000	1.280731000	-0.925781000
1	-1.269391000	1.280725000	0.925790000

Zero-Point Corrected Electronic Energy:
-1857.726048 Hartrees

Vibrational Frequencies:

Frequency (cm^{-1})	Intensity (km/mol)
110.336	1.1872
205.4665	6.4224
337.9454	0.0014
840.5961	0.7799
1027.1443	34.7766
1029.0443	0.0101

1074.2788	68.2618
1246.6906	0.197
1341.3969	30.1554
1477.8184	20.0477
1609.2648	30.8073
3105.6485	6.6836
3139.2321	2.5843
3193.6352	4.44
3236.808	7.0695

Electronic Transitions:

Wavelength (nm)	Oscillator Strength
396.18	0.0089
296.85	0.1086
268.16	0.088
254.24	0.0016
248.23	0.0828
217.51	0.2154
189.51	0
188.6	0.0124
185.99	0.2444
172.4	0.001
165.51	0.1305
162.85	0.0007
160.51	0.2655
158.06	0.0007
153	0.0083
152.82	0.0013
151.62	0.001
151.44	0.0031
151.28	0.0015
151.2	0.0286
150.93	0.0303
149.66	0.0655
149.04	0.0608
147.07	0.0446
143.11	0.006
140.54	0.0048
138.59	0.0003
136.72	0.0188
135.69	0.002
134.91	0.0005

132.3	0.0005
131.29	0.003
130.58	0.0031
129.23	0.0024
127.34	0.0031
126.98	0.0196
126.57	0.01
125.93	0.0154
122.31	0.0075
121.41	0.0013
120.94	0.0099
120.54	0.0007
118.13	0.0012
117.13	0.0062
116.67	0.0109
115.82	0.0105
114.79	0.0018
114	0.0042
113.9	0.0031
113.41	0.0032
113.17	0.0084
112.33	0.0021
111.76	0.0044
111.73	0.0062
111.58	0.0006
111.47	0.0061
111.43	0.0013
110.97	0.0011
110.73	0.0192
110.29	0.0076
109.96	0.0001
109.75	0.0005
108.32	0.0015
108.25	0.0219
107.97	0.0006
107.85	0.0076
107.68	0.0016
107.65	0.0052
107.45	0.0186
107.18	0.0051
106.81	0.0014
106.09	0.0669
106.07	0.0046

105.03	0.0024
104.77	0.0026
103.81	0.0063
103.57	0.0054
103.4	0
103.24	0.0079
103.01	0
102.89	0.0014
102.55	0.0227
102.04	0.0961
101.92	0.1204
101.9	0.0004
101.65	0.0774
101.35	0.0015
101.17	0.1348
100.8	0.0018
99.34	0.0001
99.27	0.0041
99.06	0.0009
99.02	0.027
98.9	0.0106
98.8	0.0224
98.28	0.0021
97.72	0.008
97.57	0.0001
97.22	0.0017
97.21	0.0169

$Zn^+(C_2H_4)$ Isomer 2
B3LYP
m=4

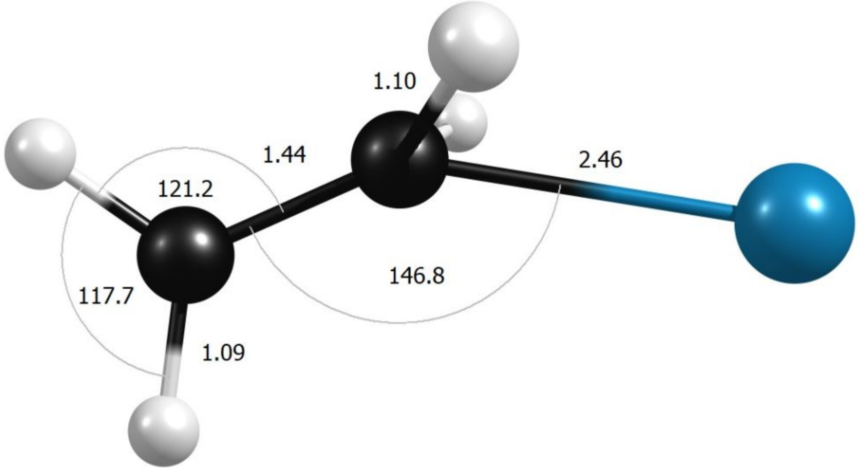

Coordinates:

30	1.076465000	-0.046713000	0.000000000
6	-2.661983000	-0.224661000	0.000000000
6	-1.345471000	0.354891000	0.000000000
1	-3.551020000	0.399323000	0.000002000
1	-2.801140000	-1.302273000	-0.000006000
1	-0.948535000	0.761488000	-0.946333000
1	-0.948534000	0.761476000	0.946336000

Zero-Point Corrected Electronic Energy:
-1857.602329 Hartrees

Vibrational Frequencies:

Frequency (cm^{-1})	Intensity (km/mol)
79.9368	0.1453
122.1183	0.006
167.8088	39.4981
457.6447	63.9088
668.3679	4.7048
689.7367	38.7088
918.7357	9.1768
1003.0491	0.4808
1142.1489	1.801
1379.5028	5.121
1384.379	10.8373

2897.248	102.187
2960.8531	1.3831
3094.0101	34.9162
3188.4773	8.8822

Electronic Transitions:

Wavelength (nm)	Oscillator Strength
358.07	0.0004
346.84	0.0141
317.4	0.0351
314.46	0.0317
279.11	0.0375
277.93	0.0156
249.83	0.0003
243.13	0
220.63	0.274
203.3	0.0764
202.13	0.2245
201.68	0.047
184.86	0.018
182.23	0.0966
177.03	0.0147
176.57	0.0131
174.29	0.002
168.9	0.0043
167.96	0.0107
166.14	0.0028
165.4	0.0001
165.16	0.001
164.18	0.1077
164.12	0.0022
164.05	0.0004
161.07	0.0213
155.22	0.001
155.2	0.0061
152.28	0.0064
149.8	0.0026
143.54	0.0048
142.86	0.005
140.15	0.0143
139.56	0.0001
138.44	0.0338

137.86	0.0008
135.83	0.004
135.01	0.0029
134.13	0.0564
133.9	0
132.24	0.0002
131.7	0.0301
130.88	0.0216
130.21	0.0213
130.02	0.0124
129.16	0.0024
129.13	0.0131
128.21	0.0116
128.19	0.0279
127.81	0.0017
127.56	0.0006
126.21	0.0145
126.01	0.0886
123.11	0.018
122.4	0.0336
121.96	0.0142
120.41	0.0008
120.35	0.0364
119.96	0.0084
119.08	0.034
118.71	0.002
118.21	0.0011
116.87	0.0131
115.17	0.0111
115.05	0.0226
114.13	0.0139
113.62	0.004
113.29	0.0139
112.4	0.017
112.26	0.0216
111.64	0.0117
111.57	0.0058
110.79	0.0032
110.59	0.0147
110.54	0.0401
110.1	0.0006
110.02	0.002
109.9	0.0002

109.24	0.0466
108.57	0.0105
107.93	0.0882
107.28	0.0014
106.93	0.0009
106.18	0.0017
105.46	0.0012
105.2	0.0009
104.86	0.0044
104.6	0.0125
104.54	0.0024
103.92	0.0152
102.97	0.0392
102.48	0.0082
101.57	0.0698
101.32	0.0135
101.26	0.0047
101.1	0.0029
101.04	0.0027
101	0.0149
100.94	0.0095
100.71	0.0091

$Zn^+(C_2H_4)$ Isomer 3
B3LYP
m=4

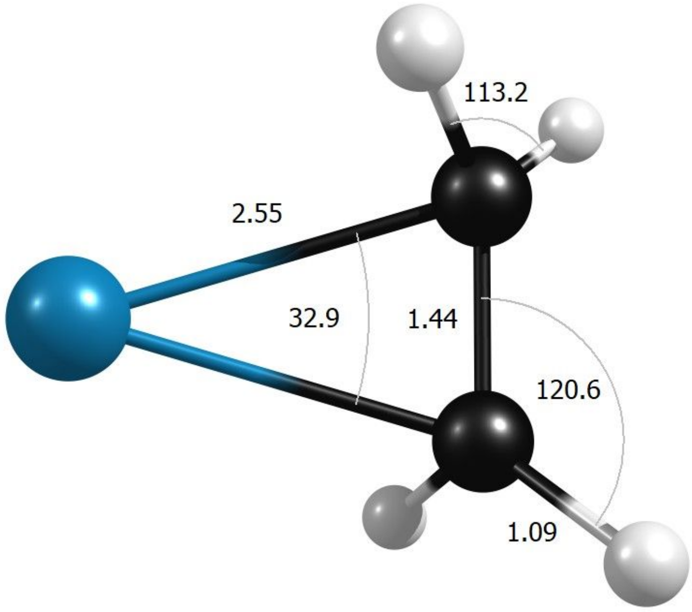

Coordinates:

30	-0.862181000	0.000002000	0.000000000
6	1.583662000	0.720349000	-0.036783000
6	1.583648000	-0.720355000	0.036779000
1	1.129330000	1.364860000	0.726706000
1	1.129266000	-1.364869000	-0.726685000
1	2.301507000	1.243921000	-0.669547000
1	2.301477000	-1.243948000	0.669543000

Zero-Point Corrected Electronic Energy:
-1857.606563 Hartrees

Vibrational Frequencies:

Frequency (cm^{-1})	Intensity (km/mol)
63.7009	0.4169
171.0555	33.2636
171.5442	4.833
556.9951	67.2313
621.3828	41.4044
748.7485	0.127
890.2661	5.1192
915.9423	5.0274

1118.7875	0.0158
1385.7872	42.8626
1416.1695	7.4635
2997.3734	38.496
2998.8351	17.6163
3101.4452	21.6655
3104.7936	20.2841

Electronic Transitions:

Wavelength (nm)	Oscillator Strength
303.58	0.0455
300.51	0.0001
264.15	0.0469
256.86	0.0152
252.12	0
236.21	0.0211
231.73	0.0193
215.98	0.001
209.13	0.0888
208.21	0.398
189.85	0.0922
178.83	0.0016
175.13	0.0072
174.92	0.0021
174.15	0.015
167.98	0.0701
167.46	0.0027
166.26	0.0041
165.64	0.0009
165.38	0.001
165.32	0.0019
165.05	0.0033
163.86	0.0578
161.2	0.0013
160.57	0.0029
159.55	0.0371
154.79	0.0798
149.62	0.0246
148.52	0.0003
146.81	0.0013
145.03	0.1533
134.54	0.0081

134.37	0.0032
134.24	0.0488
133.46	0.0047
132.92	0.0001
132.49	0.0004
131.57	0.003
130.22	0.0048
130.14	0.0317
129.36	0.0273
128.25	0.0058
127.37	0.0213
125.48	0.0005
125.35	0.0091
123.43	0.0023
122.7	0.0057
121.35	0.0024
121.34	0.0004
121.21	0.0027
120.61	0.0001
118.83	0.0187
118.59	0.0756
118.27	0.0074
117.71	0.0602
115.59	0.0008
114.27	0.0201
113.81	0.0003
113.61	0.0262
113.49	0.0063
113.39	0.0007
112.82	0.0003
112.69	0.0204
112.48	0.0184
112.45	0.0043
112.1	0.0258
111.94	0.0097
111.53	0.0133
111.52	0.012
111.34	0.0023
111.27	0.0153
111.12	0.0034
111.06	0
110.95	0.0087
110.92	0.0101

110.53	0.0246
110.26	0.0035
110.03	0.0001
109.41	0.0061
109.19	0.0386
108.81	0.0062
108.58	0.0001
108.39	0.0037
107.86	0.0022
107.24	0.001
106.75	0.0291
106.1	0.0306
105.59	0.0082
105.05	0.0007
104.77	0.0003
104.62	0.0004
103.85	0.0362
103.4	0.0377
103.24	0.0025
102.89	0.0075
102.21	0.0126
102.09	0.0107
102.04	0.031
101.93	0.0004
101.47	0.0004

$Zn^+(C_2H_4)$ Isomer 4
B3LYP
m=2

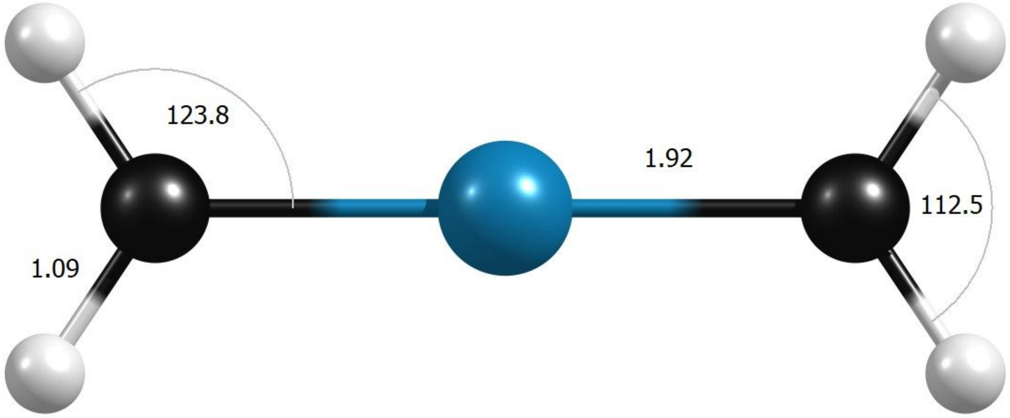

Coordinates:

30	0.000000000	0.000004000	0.000001000
6	-1.917160000	-0.000019000	-0.000011000
6	1.917156000	-0.000007000	0.000000000
1	-2.522004000	-0.904999000	0.000015000
1	-2.521844000	0.905079000	0.000021000
1	2.521923000	-0.905055000	-0.000003000
1	2.521947000	0.905015000	-0.000009000

Zero-Point Corrected Electronic Energy:
-1857.612924 Hartrees

Vibrational Frequencies:

Frequency (cm^{-1})	Intensity (km/mol)
128.04	3.7684
136.6014	3.0387
303.8431	0
534.8402	0
613.1555	199.3445
615.9844	0
679.1623	46.56
797.9093	42.526
887.3427	0
1399.4098	55.1241
1409.6846	0
3104.0188	119.4215
3105.9205	0.0147

3198.9766	0.1456
3199.3132	22.9061

Electronic Transitions:

Wavelength (nm)	Oscillator Strength
933.01	0.1029
572.1	0.0003
433.82	0
336.82	0.0058
333.76	0
260.39	0
252.59	0.002
250.71	0
235.86	0
232.69	0.0331
228.33	0
216.74	0
216.24	0
194.94	0
169.57	0
167.58	0
166.88	0.0008
164.65	0
161.99	0
155.66	0
155.55	0
155.34	0
154.52	0
154.42	0
154.08	0
150.25	0.0007
147.44	0.0141
146.45	0
144.4	0
142.73	0.2712
140.6	0
138.86	0.0024
138.79	0.0004
137.64	0.0125
137.47	0
137.21	0.3448
137.21	0

135.25	0.0666
134.43	0
131.91	0.1415
131.45	0
130.46	0
128.59	0.0128
127.3	0.0057
125.87	0
125.85	0
125.04	0
124.91	0
124.83	0
122.87	0
121.8	0.1109
121.44	0.0026
120.39	0
120.23	0
119.16	0
118.69	0.1867
117.65	0
117.53	0.0097
117.16	0.1668
117.05	0.0005
116.22	0.0039
116.19	0
115.21	0
114.05	0
112.73	0
112.38	0.1081
112.05	0
110.43	0.0071
109.8	0.0034
109.76	0
109.61	0.0074
109.61	0
108.48	0
108.46	0
108.09	0
106.15	0.0019
105.58	0
105.35	0
104.82	0
104.6	0.0226

104.3	0.0805
104.2	0.8108
103.59	0
102.75	0
102.13	0.0014
101.96	0
101.12	0.0003
101.09	0.022
100.24	0
99.87	0
99.78	0.058
99.72	0.0002
99.65	0.0012
99.53	0
99.37	0
99.36	0
98.87	0
98.59	0.001
98.26	0
98.2	0

$Zn^+(C_2H_4)$ Isomer 5
B3LYP
m=4

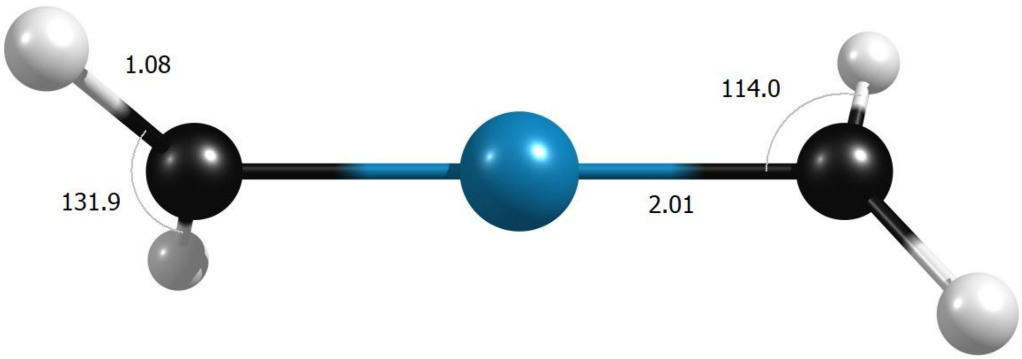

Coordinates:

30	-0.000002000	-0.000004000	0.000004000
6	-2.012575000	0.000009000	-0.000009000
6	2.012595000	0.000006000	-0.000006000
1	-2.453874000	0.633966000	0.759492000
1	-2.453883000	-0.633968000	-0.759491000
1	2.453892000	-0.759578000	0.633857000
1	2.453800000	0.759618000	-0.633899000

Zero-Point Corrected Electronic Energy:
-1857.566230 Hartrees

Vibrational Frequencies:

Frequency (cm^{-1})	Intensity (km/mol)
58.7311	0.0571
58.734	0.0571
103.2573	0.0001
379.3578	72.0002
379.4564	71.9969
389.0545	254.3802
423.622	0
558.429	11.4399
558.4386	11.4336
1062.2864	904.8417
1239.0863	0
3091.9258	221.8395
3095.3252	0.0033
3292.9	68.0354

3292.9017	68.0484

Electronic Transitions:

Wavelength (nm)	Oscillator Strength
549.57	0.1379
270.34	0.0045
270.32	0.0045
266.79	0.0074
266.79	0.0074
237.19	0.0083
237.18	0.0083
199.22	0
199.13	0
195.74	0.0001
195.67	0.0313
178.61	0.0048
176.52	0
176.4	0
174.34	0
174.34	0
173.95	0
173.94	0
165.35	0
155.85	0.038
155.85	0.038
155.41	0.7064
153.58	0.0001
153.58	0.0001
151.4	0.0003
151.4	0.0003
145.38	0
143.94	0.168
142.36	0
139.03	0.0007
139.02	0.0007
133.92	0
133.9	0
132.4	0.0944
132.39	0.0943
128.01	0.1169
128.01	0.1169
127.73	0

126.37	0.027
124.85	0
122.53	0.001
121.77	0.0002
121.77	0.0002
120.98	0.0027
120.89	0
119.4	0.021
119.4	0.021
118.4	0
118.39	0
118.39	0
118.04	0
117.82	0.0022
117.82	0.0022
117.54	0.0133
117.54	0.0133
117.32	0
117.29	0.0556
115.79	0.0581
115.14	0
114.69	0.0003
114.69	0.0003
113.82	0.0419
113.82	0.0419
113.7	0
113.64	0
113.59	0
112.89	0.0203
110.8	0.0324
110.8	0.0324
109.55	0.0046
109.55	0.0046
108.95	0.0248
108.33	0
108.25	0
107.86	0.005
107.79	0
107.04	0.0008
106.87	0
106.85	0
106.83	0.0291
106.83	0.0291

105.9	0
105.64	0.011
105.63	0.0109
104.78	0
104.42	0.2752
103.16	0
102.67	0
102.29	0
102.17	0.0006
101.71	0.0281
100.73	0.0001
100.73	0.0001
100.21	0
99.92	0
99.65	0
99.56	0.0099
99.56	0.0099
99.54	0
99.06	0.1505

$Zn^+(C_2H_4)$ Isomer 1
M06
m=2

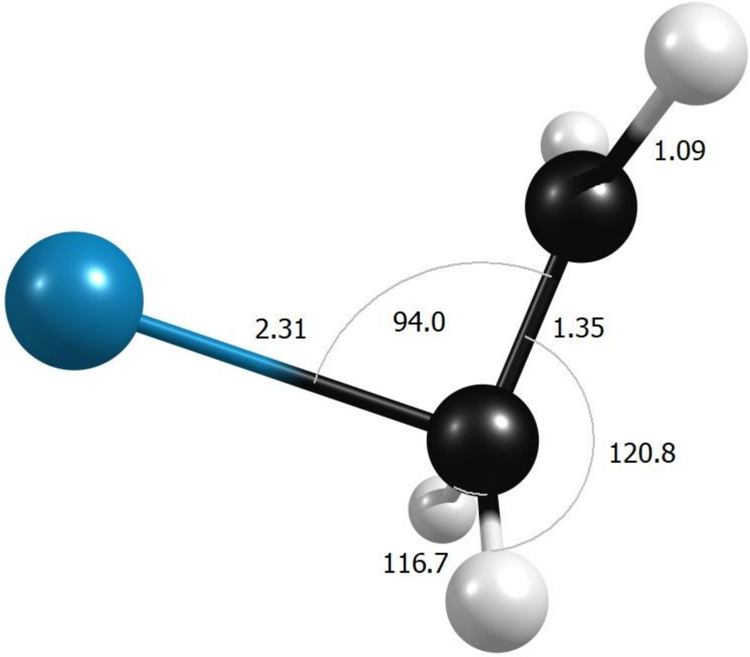

Coordinates:

30	0.855060000	0.054943000	0.000000000
6	-1.858233000	0.522801000	-0.000001000
6	-1.316832000	-0.718072000	-0.000003000
1	-2.090018000	1.041155000	0.925101000
1	-2.090013000	1.041175000	-0.925092000
1	-1.210704000	-1.279495000	0.926162000
1	-1.210682000	-1.279495000	-0.926161000

Zero-Point Corrected Electronic Energy:
-1857.560485 Hartrees

Vibrational Frequencies:

Frequency (cm^{-1})	Intensity (km/mol)
118.8381	1.0431
219.5764	4.2311
333.9276	0.0282
814.3082	0.5178
1000.4165	28.999
1011.4708	0.0896

1047.8107	57.6708
1212.5673	0.1767
1320.6629	23.6553
1433.8942	19.9747
1585.2219	34.671
3091.3275	8.0254
3122.6363	4.637
3188.8398	5.5089
3229.3184	9.3499

Electronic Transitions:

Wavelength (nm)	Oscillator Strength
398.96	0.0058
301.32	0.1324
257.24	0.0425
254.73	0.0313
241.23	0.0665
213.31	0.196
190.4	0.1304
188.37	0
187.19	0.0146
175.69	0.0014
174.24	0.1089
165.08	0.1867
164.54	0.0007
160.29	0.014
157.41	0.0005
156.88	0.1599
152.12	0.0009
150.38	0.014
149.19	0.0038
148.73	0.1377
148.46	0.0001
148.46	0.0041
148.38	0.0021
146.66	0.0009
145.39	0.0163
143.14	0.0058
142.25	0.0058
141.92	0.0022
139.74	0.0032
138.19	0.0133

137.39	0.0016
136.38	0.0021
136.32	0.0182
135.05	0.0022
132.64	0.0008
130.08	0.055
128.82	0.002
127.87	0.0134
127.39	0.0049
122.88	0.0112
122.74	0.008
122.19	0.0006
122.17	0.0004
121.03	0.0005
120.74	0.0069
119.24	0.0027
118.78	0.0028
118.13	0.0027
117.94	0.0017
117.35	0.0187
114.71	0.0024
114.69	0.006
114.6	0.0024
114.06	0.0042
113.72	0.0039
113.1	0.0006
112.69	0.0027
111.78	0.0235
111.01	0.0023
110.12	0.0007
109.72	0.0007
109.53	0.0025
108.23	0.0075
107.96	0.0015
107.79	0.0041
107.59	0.0064
107.43	0.0307
107.37	0.0288
107.28	0.0317
106.91	0.1236
106.6	0.0189
106.38	0.0021
106.24	0.0049

105.6	0.0026
105.2	0.0033
105.1	0.0009
104.9	0.0315
104.88	0.0068
104.71	0.0002
104.38	0.0515
104.06	0.0194
103.9	0.0132
102.93	0.0069
102.83	0.0081
102.4	0.0012
102.11	0.0008
102.08	0.0038
101.74	0.0828
101.31	0.0037
101.27	0
100.8	0
100.58	0.0133
100.56	0.0006
100.35	0.0002
100.25	0.0045
99.81	0.0054
99.46	0.0063
99.16	0.0009
99	0
98.92	0.0198

$Zn^+(C_2H_4)$ Isomer 2
M06
m=4

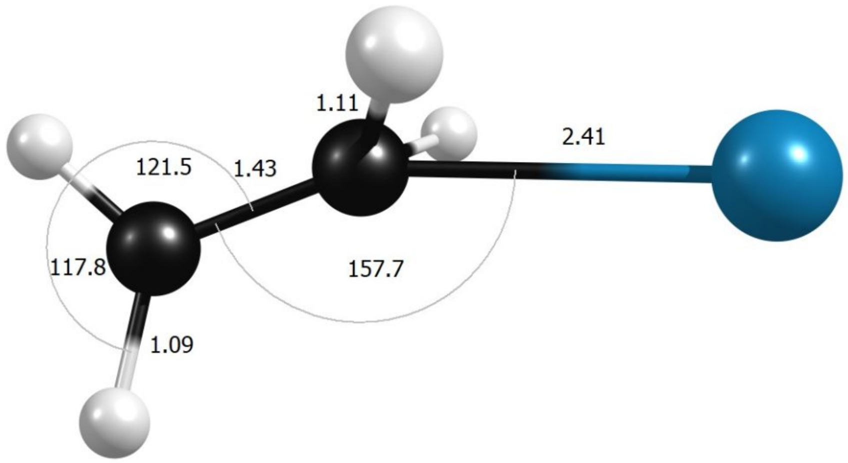

Coordinates:

30	1.074808000	-0.034951000	0.000001000
6	-2.692488000	-0.152723000	0.000000000
6	-1.316143000	0.237280000	-0.000005000
1	-3.490767000	0.583546000	0.000057000
1	-2.971181000	-1.203203000	-0.000028000
1	-0.865274000	0.580445000	-0.952262000
1	-0.865221000	0.580395000	0.952243000

Zero-Point Corrected Electronic Energy:
-1857.436826 Hartrees

Vibrational Frequencies:

Frequency (cm^{-1})	Intensity (km/mol)
85.8669	0.6514
157.6923	0.1668
186.9224	42.9378
426.703	52.1726
620.6982	26.7628
660.862	1.8322
885.9524	2.6457
1018.0551	1.1529
1166.4898	3.3922
1355.9799	4.1304
1359.3722	4.7698
2857.0973	164.7865

2929.4002	1.632
3088.8469	31.7933
3193.518	11.0564

Electronic Transitions:

Wavelength (nm)	Oscillator Strength
344.05	0.0002
332.7	0.0272
299.5	0.0181
298.11	0.0265
256.47	0.0425
251.57	0.0001
250.69	0.01
227.56	0
222.21	0
218.47	0.1815
200.8	0.1104
199.38	0.1569
196.24	0.1069
184.21	0.0188
181.56	0.0134
179.51	0.0003
177.57	0
177	0.0381
172.52	0.0055
171.64	0.0004
168.51	0.0028
166.95	0.1853
164.58	0.0003
162.43	0.0394
161.45	0.0013
160.28	0.0024
159.89	0
159.07	0.0067
158.12	0.0016
157.81	0.0013
157.01	0.0004
156.75	0
156.63	0.0001
154.76	0.0003
152.92	0.027
151.16	0.0013

149.61	0.0051
144.14	0.0016
143.19	0.0008
141.7	0.0057
139.07	0.0129
135.75	0.0073
135.24	0.0173
133.75	0.1248
132.42	0.0168
130.88	0.0241
130.12	0.0051
128.99	0.0224
126.83	0.0028
126.68	0.0027
126.55	0.0086
126.42	0.0069
125.41	0.0068
125.09	0.0239
125.08	0.0007
124.82	0.0197
124.53	0.0019
123.46	0.0305
122.61	0.0003
122.2	0.0187
121.76	0.0086
120.9	0.0194
120.14	0.0389
120.1	0.0457
117.37	0.0094
117.2	0.0001
116.66	0.0157
116.61	0.0163
116.29	0.0061
115.14	0.0069
114.47	0.0007
114.35	0.0296
114.05	0.0001
113.29	0.0005
112.29	0.0239
112.19	0.011
111.27	0.0137
110.62	0.0968
110.46	0.0286

110.11	0.0007
109.36	0.0002
109.02	0.0093
108.38	0.0037
107.19	0.0481
106.59	0.0053
105.94	0.0157
105.79	0.0613
105.62	0.0002
105.39	0.0327
104.93	0.0016
104.92	0.0123
104.76	0.0024
104.67	0.0004
104.21	0.0011
104.07	0.0121
103.6	0.0056
103.52	0.0252
102.42	0.0611
101.74	0.0269
101.19	0.076

$Zn^+(C_2H_4)$ Isomer 3
M06
m=4

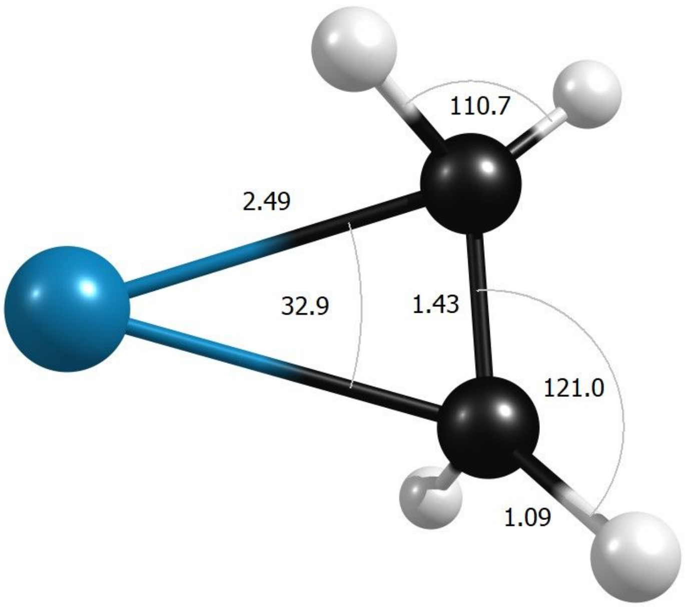

Coordinates:

30	-0.851765000	0.012182000	0.001194000
6	1.618405000	0.698416000	-0.026885000
6	1.521776000	-0.731059000	0.041941000
1	1.399890000	1.336214000	0.833781000
1	0.809886000	-1.337760000	-0.555937000
1	2.170556000	1.186036000	-0.830155000
1	2.331530000	-1.354079000	0.426146000

Zero-Point Corrected Electronic Energy:
-1857.442728 Hartrees

Vibrational Frequencies:

Frequency (cm^{-1})	Intensity (km/mol)
132.2875	4.178
183.8494	9.1476
211.9784	27.4419
523.3069	38.341
595.2322	44.0883
743.4015	0.1436
863.1107	7.426
898.1185	1.5613

1146.9791	0.2424
1368.6006	41.6736
1401.5838	4.8149
2872.5206	57.6317
3037.7422	21.4322
3090.1199	43.5758
3132.7915	11.331

Electronic Transitions:

Wavelength (nm)	Oscillator Strength
297.43	0.0116
296.18	0.017
256.99	0.0256
250.53	0.018
239.56	0.0074
236.02	0.0133
225.39	0.0107
216.97	0.0035
211.16	0.2048
203.7	0.1872
186.37	0.0922
185.65	0.0249
183.28	0.0187
181.14	0.0113
178.74	0.0018
173.24	0.0063
170.54	0.0043
168.79	0.0075
167.18	0.0099
165.89	0.1151
163.38	0.0044
161.88	0.0022
161.52	0.0096
160.53	0.0072
159.74	0.0009
158.78	0.0047
158.57	0.0006
158.37	0.0019
158.04	0.0151
155.47	0.0256
154.45	0.1134
149.68	0.0239

149.32	0.0071
147.12	0.0153
146.71	0.0167
143.91	0.0037
143.06	0.0356
140.88	0.0273
139.77	0.0028
138.58	0.0095
138.28	0.0279
137.19	0.014
134.97	0.0434
130.65	0.0092
130.32	0.0052
129.6	0.0083
129	0.0047
127.82	0.0067
124.21	0.0025
122.46	0.0124
121.82	0.0014
121.47	0.0186
121.27	0.0013
120.64	0.0156
120.53	0.0155
119.88	0.025
119.19	0.0448
118.72	0.0236
118.34	0.0183
117.95	0.002
117.37	0.0006
117.35	0.0194
116.96	0.003
116.67	0.0082
115.93	0.0246
115.86	0.0158
115.64	0.0154
114.74	0.0488
114.2	0.0454
113.15	0.0107
112.13	0.0129
111.51	0.0248
110.78	0.0184
110.43	0.0145
110.16	0.0225

109.8	0.0161
109.66	0.0114
108.91	0.0149
108.55	0.0053
107.99	0.0028
107.8	0.0239
107.28	0.0115
107.09	0.0021
106.82	0.0021
106.68	0.0089
105.58	0.0122
105.17	0.005
104.65	0.026
104.58	0.0117
104.29	0.0088
103.91	0.0222
103.8	0.0046
103.7	0.0008
103.4	0.0036
103.16	0.0045
102.59	0.0116
102.32	0.0034
101.9	0.0057
101.31	0.0054
100.86	0.0105

$Zn^+(C_2H_4)$ Isomer 4
M06
m=2

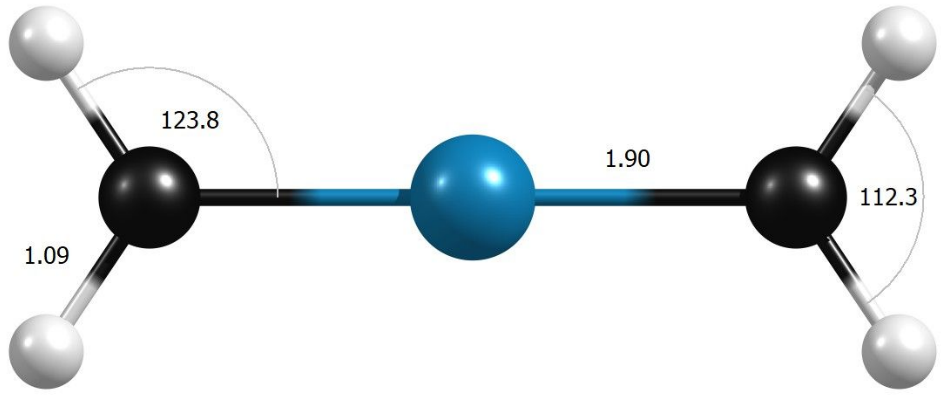

Coordinates:

30	0.000001000	-0.000006000	0.000154000
6	-1.901647000	0.000011000	-0.000246000
6	1.901644000	0.000009000	-0.000240000
1	-2.508319000	-0.904874000	-0.000370000
1	-2.508323000	0.904892000	-0.000464000
1	2.508342000	-0.904862000	-0.000477000
1	2.508292000	0.904907000	-0.000383000

Zero-Point Corrected Electronic Energy:
-1857.450931 Hartrees

Vibrational Frequencies:

Frequency (cm^{-1})	Intensity (km/mol)
128.5072	4.794
136.6018	3.5564
288.6055	0
553.6029	0
599.1361	0
616.5314	337.7271
661.4732	40.8222
741.1759	29.9689
861.1529	0
1354.5805	60.9899
1363.7289	0
3091.1623	210.8303
3093.9947	0

3199.7399	0.0001
3199.9691	31.5183

Electronic Transitions:

Wavelength (nm)	Oscillator Strength
980.42	0.1048
516.45	0.0002
409.9	0
336.25	0.0063
314.89	0
249.92	0
249.16	0
245.1	0.0192
240.37	0
231.02	0.0158
219.62	0
219.25	0
209.79	0
201.11	0
175.95	0
165.64	0
165.16	0
163.69	0.0094
163.14	0.005
161	0
159.77	0
156.61	0
148.05	0.0028
147.21	0
146.89	0
146.61	0
145.57	0
145.55	0
145.42	0.0194
145.17	0
144.65	0
143.86	0.3898
142.03	0
139.86	0
138.47	0
137.03	0.0267
134.32	0.1221

131.58	0.0268
131.47	0
130.13	0.0536
129.98	0.0009
129.57	0.0103
129.19	0
128.86	0
128.06	0
125.24	0
125.2	0.0172
124.2	0.1102
123.45	0
123.33	0.0202
122.36	0
122.32	0
122.07	0.0013
121.64	0.0253
120.02	0
120	0
119.52	0.1156
119.07	0
118.43	0.0269
118.21	0
116.73	0
116.67	0
116.58	0
116.58	0
116.35	0
116.12	0
115.85	0
114.78	0.062
114.15	0.0045
113.91	0
113.73	0.0163
113.52	0
113.1	0.1182
112.3	0.1888
110.92	0
110.18	0
109.95	0.2864
109.57	0.0474
109.55	0.282
108.94	0

108.87	0
107.84	0.3777
107.64	0.0443
106.69	0.0013
106.34	0.0032
105.95	0
105.9	0
105.4	0
105.31	0.0001
104.25	0.0222
102.92	0
102.56	0.0029
102.49	0
102.23	0
102.06	0.0002
100.97	0
100.72	0
100.48	0
100.47	0
100.04	0.1761

$Zn^{+}(C_2H_4)$ Isomer 5
M06
m=4

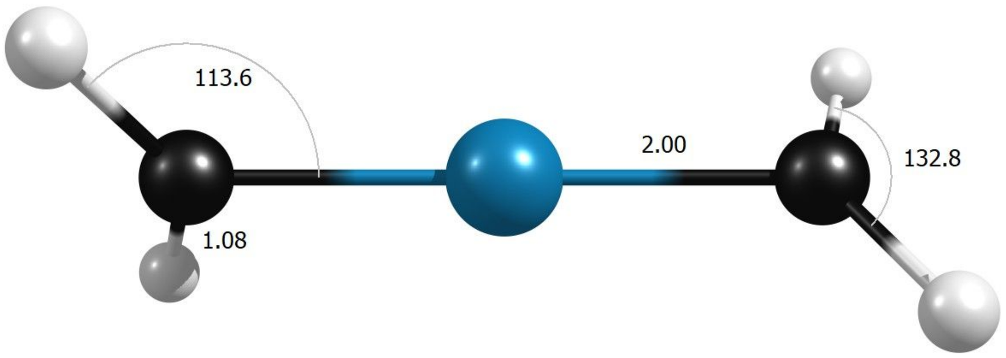

Coordinates:

30	-0.000003000	0.000001000	0.000002000
6	-1.996342000	-0.000001000	-0.000004000
6	1.996358000	-0.000003000	-0.000004000
1	-2.429749000	0.687188000	0.716398000
1	-2.429748000	-0.687195000	-0.716401000
1	2.429760000	-0.716521000	0.687065000
1	2.429732000	0.716533000	-0.687073000

Zero-Point Corrected Electronic Energy:
-1857.405614 Hartrees

Vibrational Frequencies:

Frequency (cm^{-1})	Intensity (km/mol)
65.8479	2.8202
68.2622	0.0049
68.2666	0.0049
283.7815	763.3886
318.6236	60.9295
318.6957	60.9302
435.4574	0
561.8107	12.9272
561.8266	12.9298
968.9545	1252.2693
1204.0393	0
3081.0104	175.4037
3081.7286	0.0009
3284.3203	76.1336

3284.3319	76.1392

Electronic Transitions:

Wavelength (nm)	Oscillator Strength
585.31	0.1587
273.41	0.0017
273.41	0.0017
255.35	0.0114
255.34	0.0114
235.58	0.0087
235.57	0.0087
203.77	0
203.59	0
197.62	0
196.99	0.0496
179.05	0
178.9	0
168.92	0.0058
164.9	0
162.74	0
162.74	0
162.26	0.0002
162.26	0.0002
161.9	0.0137
161.9	0.0137
154.28	0.6348
154.18	0.0028
154.18	0.0029
152.85	0.0218
152.85	0.0218
151.34	0.0036
151.33	0.0036
147.59	0
147.41	0
144.67	0
140.77	0
140.59	0.0474
139.74	0.1164
139.73	0.0005
135.26	0.0045
135.25	0.0045
133.5	0.1543

133.5	0.1543
130.49	0
130.32	0.0021
130.32	0.0021
126.45	0
126.23	0.0118
126.15	0
125.66	0
123.73	0.0003
121.98	0.1915
121.63	0.0025
121.63	0.0025
120.93	0
120.02	0.0218
119.51	0
117.62	0.0008
117.62	0.0008
116.66	0.0075
116.66	0.0076
115.94	0.0042
115.94	0.0041
115.7	0.0198
115.7	0.0199
115.48	0.0477
115.48	0.0477
113.56	0
113.4	0.0218
113.37	0
113.04	0.0084
113.04	0.0083
112.8	0
112.8	0
112.36	0.0341
112.36	0.0341
111.84	0
111.67	0.0536
110.51	0.0383
110.5	0.0383
110.41	0
109.5	0
109.31	0.0034
109.31	0
109.3	0

108	0.0107
107.19	0
107.08	0.002
106.45	0
106.44	0
106.26	0.003
106.26	0.003
106.18	0.0273
106.17	0
105.26	0
104.9	0.1022
104.9	0.1022
104.09	0
103.66	0
103.61	0.3275
103.16	0
102.88	0.0001
102.88	0.0001
102.33	0.0037

$Zn^+(C_2H_4)$ Isomer 1
M06-L
m=2

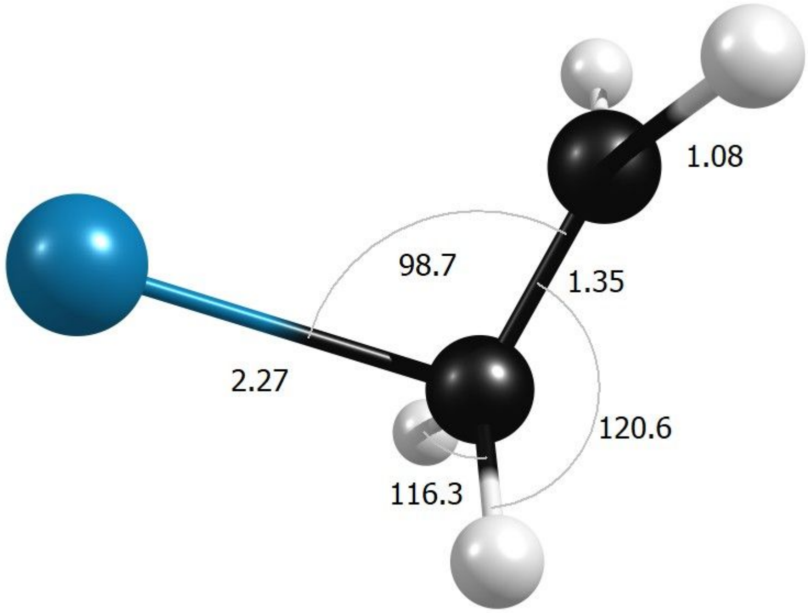

Coordinates:

30	0.859939000	0.062874000	0.000000000
6	-1.917879000	0.484803000	-0.000001000
6	-1.271490000	-0.705974000	-0.000002000
1	-2.190045000	0.985175000	0.922378000
1	-2.190043000	0.985198000	-0.922367000
1	-1.140941000	-1.264787000	0.923802000
1	-1.140925000	-1.264793000	-0.923801000

Zero-Point Corrected Electronic Energy:
-1857.598108 Hartrees

Vibrational Frequencies:

Frequency (cm^{-1})	Intensity (km/mol)
135.993	0.4874
232.3882	3.5597
334.7293	0.273
827.4054	0.5436
970.7534	20.8024
1026.7316	0.1688
1047.7177	54.0963
1233.9279	0.3058

1327.4134	12.9696
1455.229	15.3717
1590.7189	14.0977
3102.2718	9.3228
3140.9692	6.0612
3200.9383	5.5309
3249.6578	6.0948

Electronic Transitions:

Wavelength (nm)	Oscillator Strength
406.17	0.0188
288.13	0.1303
263.43	0.0132
257.78	0.0891
243.86	0.0961
226.29	0.175
191.31	0.0028
187.19	0
178.92	0.2256
170.92	0.0014
166.95	0.0022
163.18	0.1258
162.62	0.0088
162.61	0
161.93	0.0023
160.38	0.0782
158.87	0.0037
157.15	0.1635
155.02	0.0003
152.36	0.0128
150.99	0.0021
150.27	0.0031
147.99	0.1442
145.68	0.0549
144.95	0.0112
139.76	0.0116
138.98	0.0008
138.88	0.0041
137.43	0.0043
134.18	0.0091
132.32	0.0025
129.96	0

128.64	0
128.39	0.0081
127.12	0.011
126.54	0.0028
125.89	0.0057
123.67	0.0024
123.58	0.0062
122.86	0.0001
122.57	0.0011
122.57	0.0021
121.36	0.0031
121.35	0
120.32	0.0189
120.02	0.0009
119.41	0.0024
119.32	0.0009
118.97	0.0018
118.27	0.0545
117.04	0.0002
116.98	0.0142
116.51	0.0143
116.2	0.013
116	0.0247
114.91	0.0071
114.77	0.0028
113.96	0.0069
113.43	0.0058
112.24	0.0021
111.21	0.0211
110.93	0.0061
110.43	0.0102
109.48	0
109.24	0.0005
108.91	0.0003
108.79	0.0187
108.66	0.0036
108.26	0.0132
107.72	0.004
107.4	0.0004
106.84	0.0007
105.98	0
105.9	0.0315
105.74	0.0016

105.65	0.0074
105.05	0.0004
104.76	0.0058
104.69	0.0095
104.49	0.0757
104.27	0.0054
103.93	0.0009
103.65	0.0048
103.35	0.0018
103.19	0
102.86	0.0112
102.52	0.1422
102.29	0.0012
102.23	0.0215
102.12	0.0062
101.92	0.0244
101.66	0.007
101.5	0.0009
100.89	0.0121
100.84	0.0964
100.67	0.0012
100.43	0.0017
99.88	0.0336
99.47	0.058
99.18	0.0015

$Zn^+(C_2H_4)$ Isomer 2
M06-L
m=4

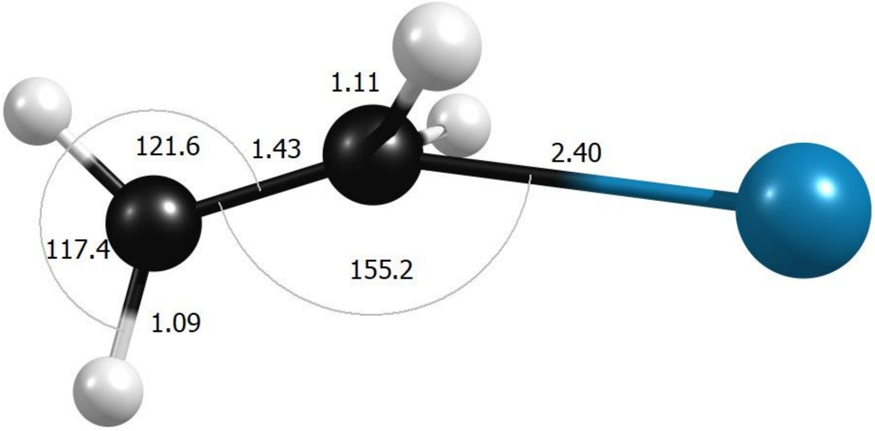

Coordinates:

30	1.067542000	0.038262000	0.000000000
6	-2.670676000	0.168262000	0.000000000
6	-1.308889000	-0.262656000	0.000000000
1	-3.491843000	-0.541253000	-0.000002000
1	-2.922825000	1.224266000	0.000003000
1	-0.867108000	-0.632258000	0.944643000
1	-0.867107000	-0.632253000	-0.944644000

Zero-Point Corrected Electronic Energy:
-1857.479710 Hartrees

Vibrational Frequencies:

Frequency (cm^{-1})	Intensity (km/mol)
82.5854	0.0329
181.7835	0.1451
191.9896	42.8566
406.3889	49.6866
653.08	23.0289
678.563	2.1899
895.3336	2.4261
1027.9084	1.3857
1179.3554	2.1187
1369.3461	7.9476
1382.3623	3.5469
2883.697	140.4689

2953.3405	1.5876
3104.8353	33.9055
3208.7752	10.7517

Electronic Transitions:

Wavelength (nm)	Oscillator Strength
346.85	0.0003
336	0.0227
295.32	0.0406
290.39	0.0406
283.47	0.0263
259.66	0.0347
241.05	0.0009
227.21	0
212.75	0.1267
207.45	0.1243
202.19	0.4061
188.61	0.0015
182.02	0.0183
177.32	0.0143
174.86	0.0011
174.41	0
173.67	0.0002
173.42	0.0013
172.56	0
169.4	0.0429
167.57	0
165.56	0.1419
163.34	0.0022
162.56	0.0122
159.88	0
156.64	0.0025
155.78	0.0012
154.24	0.0024
149.1	0.001
146.93	0.0396
144.21	0.0011
142.2	0.0011
139.81	0.037
139.44	0.001
139.24	0.0115
138.33	0.0012

136.78	0.0002
135.62	0.0179
134.13	0.0601
132.84	0.0065
131.6	0.0006
130.03	0.0209
129.51	0.0095
128.92	0.0276
128.7	0.0032
128.17	0.0025
127.71	0.0043
127.01	0.0156
126.18	0.048
125.42	0.0002
125.31	0.0269
125.13	0.0141
124.64	0.0107
124.36	0.0584
124.33	0.0002
123.42	0.0025
123.02	0.0002
122.87	0.0296
120.73	0.0005
119.57	0.0089
119.27	0.0207
118.51	0.0018
118.4	0
117.97	0.0089
117.77	0.0082
117.37	0.0064
116.97	0.0107
116.33	0.0246
116.24	0.0348
114.03	0.0439
113.42	0.0018
113.01	0.0014
112.09	0.0079
111.64	0.0219
111.16	0.0216
110.97	0.0064
110.91	0.0047
110.76	0.0053
110.66	0

110.27	0.0005
110.23	0.0237
109.69	0.0309
108.76	0.0276
108.57	0.0604
107.24	0.0012
107.12	0.056
106.47	0.0037
105.44	0.017
104.95	0.0103
104.8	0.0014
104.8	0.0242
104.57	0.0083
104.39	0.0017
104.3	0.0103
103.92	0.0003
103.73	0
103.35	0.0046
103.31	0.0039
103.21	0.0012
102.94	0.0019

$Zn^+(C_2H_4)$ Isomer 3
M06-L
m=4

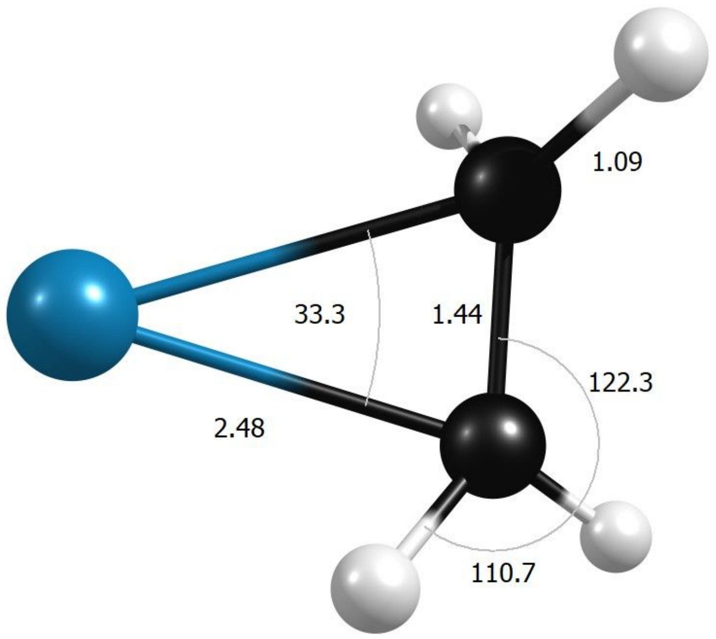

Coordinates:

30	-0.845204000	0.010348000	-0.001311000
6	1.593867000	0.702132000	0.028975000
6	1.516487000	-0.729326000	-0.043544000
1	2.152511000	1.194260000	0.823962000
1	0.829057000	-1.343521000	0.567783000
1	1.379204000	1.339163000	-0.831340000
1	2.333211000	-1.337188000	-0.433671000

Zero-Point Corrected Electronic Energy:
-1857.485417 Hartrees

Vibrational Frequencies:

Frequency (cm^{-1})	Intensity (km/mol)
106.2804	2.4609
180.1174	15.7725
200.194	21.8676
511.9533	39.4038
601.8958	40.702
749.6934	0.1921
871.6839	6.2596

912.2361	1.7796
1148.4024	0.1669
1380.6534	41.1075
1416.2779	4.3973
2922.0209	45.7549
3059.155	22.501
3114.8637	37.825
3149.7856	9.4441

Electronic Transitions:

Wavelength (nm)	Oscillator Strength
289.96	0.0554
280.3	0.0009
252.47	0.0562
248.12	0.0017
238.55	0.018
234.61	0.0163
224.65	0.0292
211.34	0.0809
200.8	0.0125
199.37	0.3059
190.03	0.1992
178.96	0.0007
176.91	0.0002
176.05	0.0011
175.21	0.0003
174.26	0.0026
172.44	0.0061
170.12	0.0026
167.08	0.0098
164.74	0.0025
162.99	0.059
160.9	0.052
159.63	0.01
158.07	0.068
156.13	0.0522
154.01	0.005
151.26	0.0093
144.79	0.0135
143.79	0.0048
140.85	0.0046
139.64	0.0365

136.99	0.0066
135.52	0.0318
134.26	0.0044
133.85	0.0238
132.67	0.0059
131.31	0.0034
129.88	0.0263
129.58	0.0164
128.89	0.0152
128.2	0.0226
127.79	0.0048
127.21	0.0034
126.86	0.0064
126.71	0.003
126.18	0.0015
125.25	0.0019
125.2	0.0016
125.06	0.0075
124.74	0.0104
124.46	0.0063
124.18	0.0049
124.08	0.0083
124.01	0.002
123.86	0.0175
123.05	0.0158
122.36	0.0214
122.23	0.0177
121.72	0.023
120.99	0.0312
119.93	0.0292
119.69	0.0281
117.84	0.0015
117.09	0.0022
116.68	0.0303
116.02	0.025
115.83	0.0435
115.2	0.0198
114.89	0.01
113.61	0.0099
113.44	0.0088
113.32	0.0078
111.77	0.0173
110.49	0.0079

110	0.0113
109.26	0.0047
109.04	0.043
108.39	0.0044
108.12	0.0337
107.07	0.0122
106.9	0.0041
106.64	0.0008
106.42	0.0011
106.22	0.0017
106.1	0.0006
105.6	0.0092
105.5	0.0047
105.3	0.0097
105	0.0016
104.68	0.0027
104.52	0.0069
104.27	0.014
103.57	0.0019
103.47	0.0048
103.37	0.002
103.29	0.0153
103.22	0.0131
102.98	0.0225
102.85	0.0066
102.52	0.0272

$Zn^+(C_2H_4)$ Isomer 4
M06-L
m=2

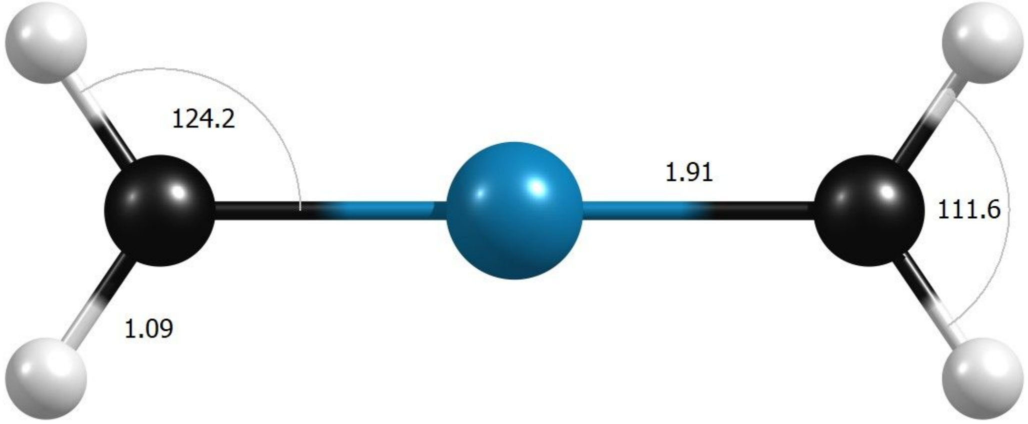

Coordinates:

30	0.000001000	-0.000001000	-0.000001000
6	-1.908103000	0.000000000	-0.000009000
6	1.908097000	-0.000004000	0.000009000
1	-2.520588000	-0.900586000	0.000031000
1	-2.520574000	0.900592000	0.000025000
1	2.520601000	-0.900563000	-0.000014000
1	2.520560000	0.900599000	-0.000009000

Zero-Point Corrected Electronic Energy:
-1857.496829 Hartrees

Vibrational Frequencies:

Frequency (cm^{-1})	Intensity (km/mol)
130.9034	3.7379
138.6863	2.9148
302.3193	0
541.7208	0
597.6634	0
625.6671	117.0864
663.9106	41.4025
763.9759	33.9411
856.1035	0
1376.3233	29.9178
1387.0114	0
3104.5744	107.6042

3106.538	0.0096
3212.8422	0.5656
3212.9403	19.6391

Electronic Transitions:

Wavelength (nm)	Oscillator Strength
821.91	0.1015
497.81	0.0003
396.5	0
324.61	0
297.35	0.0072
263.38	0
261.3	0.0074
248.44	0
227.13	0
213.7	0
208.35	0
206.85	0.025
206.73	0
183.92	0
177.45	0
177.13	0
175.8	0
175.78	0
171.93	0
170.51	0
167.23	0
158.1	0
157.66	0.0008
150.81	0.0001
150.7	0.0008
150.56	0.0011
149.21	0
149.13	0
148.46	0
146.15	0.003
144.66	0.0034
140.48	0.0079
139.91	0
139.8	0
135.81	0
135.76	0

135.7	0
134.54	0.6062
134.01	0
133.54	0
133.38	0
132.21	0
131.71	0.1881
130.05	0
127.89	0
126.9	0
126.89	0.0304
124.54	0.0036
122.98	0.027
120.3	0
119.61	0
119.1	0.1521
118.41	0
118.28	0
118.26	0.1834
116.94	0.005
116.79	0
116.71	0
116.47	0
114.47	0.0006
114.14	0
113.29	0.0637
112.9	0
112.74	0.0763
110.92	0
110.64	0
109.18	0.0379
109.1	0
108.58	0.0427
108.39	0.1587
107.84	0
107.52	0.2369
106.69	0
106.6	0
106.26	0.0173
105.74	0
105.45	0
105.27	0
104.64	0.0917

104.05	0.0027
103.8	0
103.66	0
103.04	0.2141
102.6	0.001
101.58	0.1513
101.5	0.0531
101.27	0.0001
101.21	0.0013
100.61	0
100.47	0
100.26	0.0262
100.18	0
99.65	0
99.64	0
99.39	0
99.32	0.0018
99.03	0.0166
99	0
98.64	0.0097
98.61	0.0116

$Zn^+(C_2H_4)$ Isomer 5
M06-L
m=4

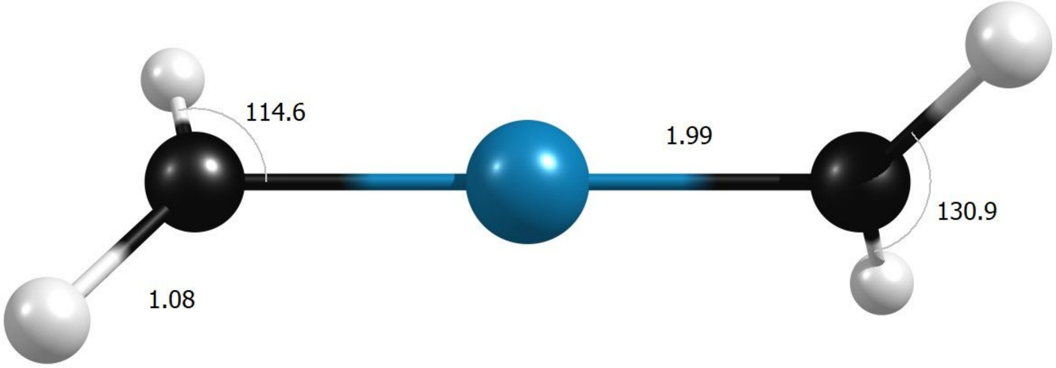

Coordinates:

30	0.000000000	-0.000028000	0.000024000
6	-1.987619000	0.000051000	-0.000040000
6	1.987618000	0.000063000	-0.000055000
1	-2.437986000	-0.701840000	0.691458000
1	-2.437900000	0.701961000	-0.691574000
1	2.437946000	-0.691517000	-0.701890000
1	2.437940000	0.691553000	0.701873000

Zero-Point Corrected Electronic Energy:
-1857.447796 Hartrees

Vibrational Frequencies:

Frequency (cm^{-1})	Intensity (km/mol)
40.7821	1.6148
40.7855	1.6146
151.7405	0.1014
241.3131	55.352
241.3153	55.3526
325.2539	591.1965
440.553	0
540.1654	13.8771
540.1664	13.8769
1006.6379	1300.3769
1227.9596	0
3082.051	0.0044
3082.0907	149.9207
3284.016	64.0246

3284.0167	64.0247

Electronic Transitions:

Wavelength (nm)	Oscillator Strength
583.92	0.1343
292.62	0.0021
292.62	0.0021
255.62	0.0102
255.62	0.0102
220.14	0.0072
220.14	0.0072
201.2	0.0175
197.93	0
197.92	0
197.08	0
197.08	0
191.22	0
190.64	0
187.16	0
185.16	0.0897
171.01	0
170.86	0
159.43	0.0015
159.03	0
157.91	0
146.65	0.806
146.62	0.0093
146.62	0.0091
144.09	0.0068
144.09	0.0069
143.45	0.0244
143.45	0.0244
140.39	0.0014
140.39	0.0014
140.18	0
132.84	0
132.47	0
132.28	0
131.82	0.004
131	0
130.6	0.0602
130.6	0.0601

130.4	0.0197
129.71	0.0015
129.71	0.0015
129.09	0
129.09	0
128.4	0
128.23	0
127.5	0.0654
127.5	0.0654
127.26	0
127.16	0.0835
124.07	0.0729
124.07	0.0728
123.05	0
123.05	0
117.79	0.0099
117.28	0
116.49	0.0022
116.49	0.0022
113.99	0
113.81	0
113.44	0
113.04	0.072
112.33	0
112.24	0
112.17	0.0005
112.17	0.0005
111.82	0.0275
111.82	0.0275
109.9	0
109.72	0
109.33	0.0189
109.24	0
108.86	0.0317
108.26	0
108.26	0
107.4	0.0005
107.4	0.0005
106.53	0
106.39	0.0446
106.39	0.0446
106.17	0
106	0

104.83	0.3396
104.7	0.0399
104.7	0.0399
103.94	0
103.94	0.0001
103.61	0
102.74	0.0155
102.29	0.0525
102.29	0.0525
102.15	0
101.93	0.0205
101.93	0.0205
101.48	0.0007
101.06	0
100.1	0
99.77	0.0115
99.77	0.0115
97.74	0.0767
97.2	0

$Zn^{+}(C_2H_4)$ Isomer 1
MN15-L
m=2

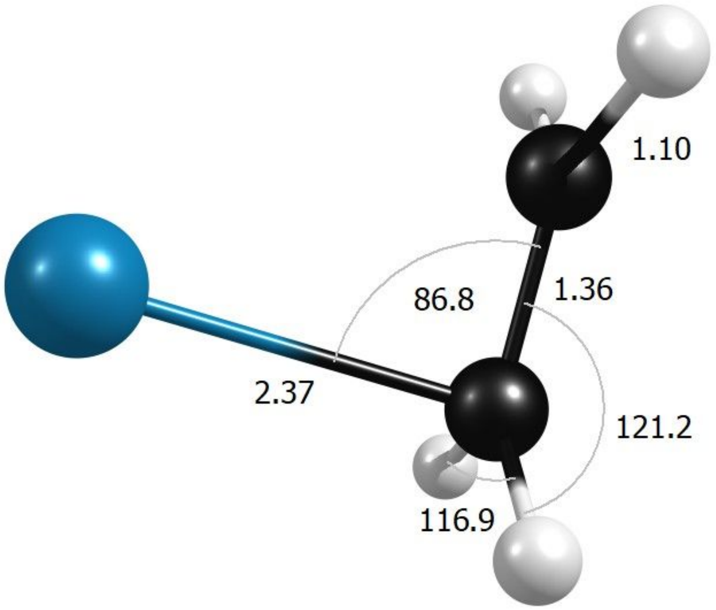

Coordinates:

30	0.847841000	0.040139000	0.000000000
6	-1.762864000	0.583876000	0.000000000
6	-1.393901000	-0.728878000	0.000000000
1	-1.928973000	1.131291000	0.935176000
1	-1.928972000	1.131289000	-0.935177000
1	-1.318345000	-1.298370000	0.936325000
1	-1.318341000	-1.298364000	-0.936328000

Zero-Point Corrected Electronic Energy:
-1857.571286 Hartrees

Vibrational Frequencies:

Frequency (cm^{-1})	Intensity (km/mol)
73.7624	2.812
199.8255	10.0683
309.927	0.57
817.2177	0.2609
1004.5724	18.3419
1062.5071	0.0097
1075.2457	93.4849
1240.48	0.1374

1357.0416	30.5618
1465.5457	12.9561
1610.4087	38.3496
3099.195	7.0184
3128.1432	2.4837
3198.1212	2.9036
3237.199	6.6356

Electronic Transitions:

Wavelength (nm)	Oscillator Strength
421.98	0.0188
291.96	0.1128
278.43	0.0697
247.16	0.0724
231.91	0.0003
220.67	0.1568
187.62	0.352
182.7	0
173.19	0.007
169.79	0.0004
169.17	0.0011
166.26	0.1201
161.2	0.3095
154.2	0.0135
152.29	0.0319
151.7	0.0044
151.24	0.0066
150.66	0.0021
150.62	0.0015
150.23	0.1095
149.7	0.0247
149.21	0.0091
147.58	0.0495
144.33	0.0002
143.07	0.0003
139.87	0.0095
137.65	0.0008
136.61	0.0049
133.54	0.0037
132.68	0.0061
132.35	0.0002
131.7	0.0016

130.34	0.017
130.26	0.0154
129.78	0.0264
125.98	0.0097
125.82	0.0014
125.06	0.0178
123.04	0.0074
122.75	0.0115
122.17	0.001
121.98	0.0016
121.49	0.0026
121.46	0.0021
121.28	0.0002
120.08	0.0001
119.74	0.0006
118.82	0.002
118.36	0.0007
118.29	0.0056
117.88	0.0056
117.27	0.0593
116.32	0.0013
115.77	0.0206
115.74	0.0035
115.52	0.017
113.53	0.001
112.42	0
112.18	0.0141
111.88	0.0046
111.51	0.0082
110.81	0.0198
110.62	0.0203
110.36	0.008
109.79	0.0097
109.66	0.0014
109.22	0.0049
108.34	0.0012
108.22	0.0008
108.19	0.0018
107.89	0.0001
107.01	0.0036
106.64	0.0048
106.61	0.0078
105.56	0.0043

104.89	0.0018
104.63	0.0178
104.62	0.0016
104.32	0.0067
104	0.0141
103.85	0.0881
103.3	0.0186
103.07	0.0053
102.64	0.0001
102.34	0
102.06	0.1827
101.61	0.0649
101.09	0.0195
100.99	0.0001
100.76	0.1487
100.52	0.0002
100.21	0.0007
100.01	0.0003
99.9	0.0164
99.49	0.0326
99.26	0.0027
99.2	0.0021
98.78	0.0001
98.48	0.0118
98.48	0.0021

$Zn^+(C_2H_4)$ Isomer 2
MN15-L
m=4

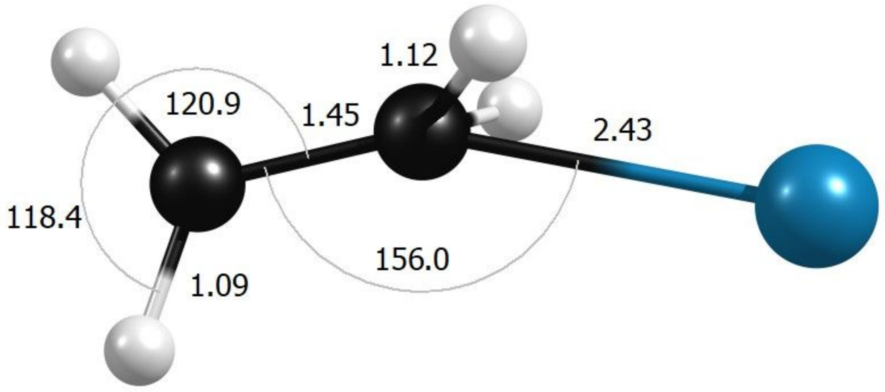

Coordinates:

30	1.081596000	0.037531000	0.000000000
6	-2.709114000	0.166043000	0.000001000
6	-1.326588000	-0.257739000	0.000000000
1	-3.521023000	-0.566429000	0.000001000
1	-2.966989000	1.228581000	0.000004000
1	-0.872819000	-0.618961000	0.954733000
1	-0.872827000	-0.618947000	-0.954745000

Zero-Point Corrected Electronic Energy:
-1857.449808 Hartrees

Vibrational Frequencies:

Frequency (cm^{-1})	Intensity (km/mol)
97.812	0.1054
174.3956	40.7972
186.4791	0.2826
430.2114	46.4137
641.0729	5.2782
659.6742	17.4272
912.5759	1.7983
1024.2911	1.9782
1164.5164	0.4912
1391.5908	6.396
1394.3628	4.7196
2866.038	178.5156
2939.6056	0.7217

3125.5718	23.3859
3236.1111	8.5555

Electronic Transitions:

Wavelength (nm)	Oscillator Strength
385.13	0.0003
373.68	0.036
316.41	0.0536
313.5	0.0418
258.18	0.0026
244.98	0.0252
236.99	0.0136
228.44	0.0903
225.69	0.1308
211.3	0.4453
202.71	0.0058
198.46	0.0001
175.87	0.233
175.23	0.0024
169.88	0.0196
167.33	0.0098
166.35	0.0523
161.68	0.0064
160.56	0.0003
160.23	0
159.96	0.0005
159.21	0.0004
157.05	0.0251
156.77	0.0012
155.4	0.006
152.81	0.0071
152.6	0.0031
150.58	0.0023
148.16	0.0002
146.89	0.0303
143.78	0.0018
143.75	0.0028
142.12	0.002
142.11	0.0155
138.46	0.015
138.46	0
136.1	0.0008

135.15	0.0007
132.56	0.0063
130.71	0.0085
130.46	0.0037
130.02	0.0014
129.05	0.0035
128.34	0.0018
128.24	0.0208
127.01	0.0108
126.62	0.0264
126.13	0.0801
125.34	0.0047
124.55	0.0585
124.24	0.002
124.18	0.0121
121.6	0
121.46	0.001
121.38	0.039
120.84	0.0114
120.63	0.0129
120.37	0.1145
118.9	0.0154
118.42	0.0096
118.4	0.0013
117.51	0.0354
117.38	0.0048
117.29	0.0355
117.03	0.0019
116.55	0.0144
116.37	0.0005
116.15	0
115.98	0.027
113.5	0.0025
112.48	0.0034
112.44	0.0268
112	0.0404
111.85	0.003
110.83	0.0309
110.24	0.0054
110.21	0.0018
109.81	0.0035
108.69	0.0328
108.59	0.076

107.61	0.0035
107.44	0.0003
107.38	0.0021
107.09	0.0004
106.95	0.0349
106.83	0.0085
106.48	0.0316
106.24	0.002
105.95	0.0082
105.86	0.0247
105.16	0.0039
105.12	0.0006
104.97	0.0051
104.94	0.018
104.71	0.0117
104.31	0.0067
104.07	0.0087
104.05	0.0146
103.83	0.0076
103.62	0.0009

$Zn^+(C_2H_4)$ Isomer 3
MN15-L
m=4

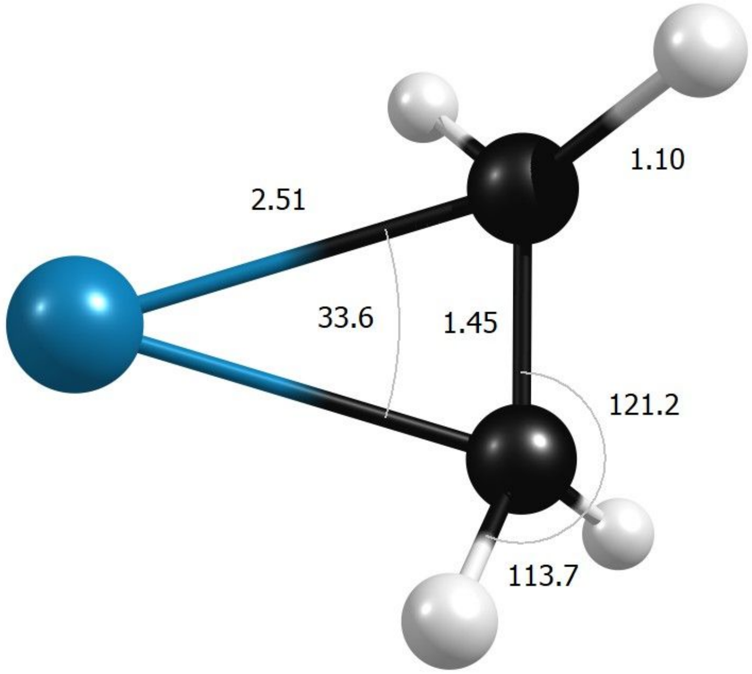

Coordinates:

30	-0.847017000	-0.000002000	0.000000000
6	1.558628000	0.726480000	0.034546000
6	1.558643000	-0.726475000	-0.034546000
1	2.267393000	1.266088000	0.676158000
1	1.086038000	-1.361209000	0.739522000
1	1.086021000	1.361202000	-0.739530000
1	2.267424000	-1.266068000	-0.676150000

Zero-Point Corrected Electronic Energy:
-1857.456431 Hartrees

Vibrational Frequencies:

Frequency (cm^{-1})	Intensity (km/mol)
112.8495	0.0497
182.2838	31.4442
217.8244	7.2736
545.4465	47.8343
597.0962	28.5999
734.9069	3.906

880.1958	3.5218
916.2863	2.0636
1135.7301	0.0041
1393.9426	33.5373
1427.9388	3.5705
3013.3838	36.0119
3015.7694	9.9955
3148.0029	18.8089
3150.6961	16.8346

Electronic Transitions:

Wavelength (nm)	Oscillator Strength
323.98	0.0697
308.88	0.0002
269.91	0.0723
258.7	0.0306
224.96	0.0866
218.98	0.0001
213.64	0.0169
212.41	0.0118
204.93	0.0155
201.4	0.4542
196.48	0.0975
166.77	0.0947
165.57	0.0003
164.87	0.0163
164.23	0.0001
164.14	0.0001
163.94	0.0006
163.93	0.0024
161.89	0.0084
160	0.0012
158.24	0.0019
157.3	0.0041
156.98	0.0118
156.02	0.0027
155.69	0.0145
148.3	0.0078
147.27	0.0103
145.99	0.0013
145.63	0.0108
144.25	0.0004

142.48	0.2092
134.41	0.0501
134.22	0.0017
133.65	0.0071
133.02	0.0015
131.8	0.0089
131.74	0.0218
130.66	0.0038
130.57	0.0141
129.79	0.0326
129.65	0.0112
128.17	0.0643
127.47	0.0138
124.62	0.0031
123.99	0.002
122.49	0.0016
121.13	0.0004
121.02	0.0075
120.9	0
120.66	0.0106
120.59	0.0193
120.09	0.0012
119.62	0.0071
119.39	0.0029
118.89	0.003
118.65	0.0005
118.53	0.0044
118.33	0.0132
118.08	0.0009
117.97	0.0002
117.69	0.0017
117.42	0.0002
116.59	0.0225
116.33	0.0562
116	0.1197
114.63	0.0114
113.63	0.0312
113.31	0.0004
112.62	0.0026
112.35	0.0245
111.89	0.0359
111.27	0.0003
111.01	0.0115

110.71	0.0136
109.65	0.0483
109.55	0.001
109.47	0.001
109.12	0.0043
108.64	0.0135
108.54	0.0002
108.29	0.0295
107.92	0
107.85	0.0216
107.44	0.0043
107.13	0.0001
106.22	0.0002
106.03	0.0044
105.97	0.0097
105.19	0.0004
105.18	0.0233
105	0.0041
104.61	0.0434
103.38	0.0126
103.08	0
102.41	0.0048
102.38	0.0276
102.15	0.0067
102.04	0.0034
101.98	0.0089
101.62	0.0028

$Zn^+(C_2H_4)$ Isomer 4
MN15-L
m=2

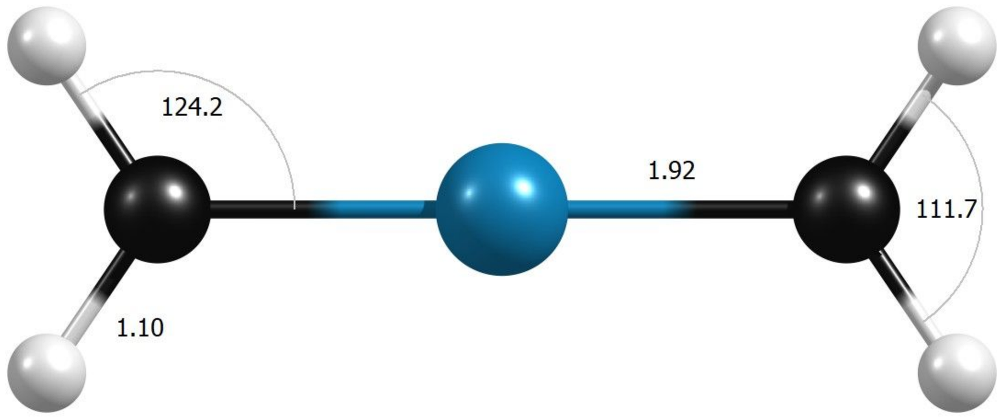

Coordinates:

30	-0.000001000	-0.000006000	-0.000001000
6	-1.922578000	0.000010000	0.000014000
6	1.922584000	0.000009000	-0.000002000
1	-2.540984000	-0.910867000	-0.000063000
1	-2.540989000	0.910888000	-0.000003000
1	2.540995000	-0.910868000	0.000035000
1	2.540974000	0.910903000	-0.000025000

Zero-Point Corrected Electronic Energy:
-1857.467715 Hartrees

Vibrational Frequencies:

Frequency (cm^{-1})	Intensity (km/mol)
126.3105	2.6794
135.5126	2.1985
310.2175	0
537.2285	0
587.2357	0
625.5013	103.5988
653.4201	45.9602
781.6555	31.8135
864.2017	0
1391.0204	24.4815
1400.0436	0
3093.7463	160.2836
3097.8443	0.0013

3200.2556	17.4572
3200.4551	0.0318

Electronic Transitions:

Wavelength (nm)	Oscillator Strength
754.52	0.1177
426.81	0.0006
357.13	0
307.34	0
277.04	0.0115
245.38	0
243.8	0.0012
230.53	0
219.18	0
218.11	0.0566
211.68	0
200.69	0
194.52	0
190.26	0
168.5	0
167.79	0
167.78	0
164.82	0
163.29	0
163.05	0
159.91	0
159.43	0
159.36	0.001
157.02	0.0029
156.58	0
148.1	0
146.71	0
144.22	0.0105
144.01	0.0033
142.9	0.0015
141.44	0.0051
141.33	0
140.67	0
138.22	0.0007
136.76	0.5709
136.53	0.2051
136.17	0

134.03	0
133.66	0.0654
131.56	0
130.87	0
130.49	0.0331
128.65	0
126.6	0
126.6	0
125.41	0
125.14	0
123.81	0
122.91	0.0154
122.73	0.0021
121.1	0.1833
120.99	0
120.44	0
118.97	0.0957
118.73	0
118.54	0
118.3	0.1867
117.61	0
116.98	0
116.52	0.0003
114.2	0.0539
113.93	0
113.52	0.0702
112.88	0
112.7	0.0102
112.06	0.0022
110.78	0
110.31	0.0082
110.3	0
108.96	0
107.95	0
107.39	0
106.83	0.0088
106.45	0
106.34	0
105.88	0.0296
105.87	0
105.83	0.0235
105.03	0
104.56	0

104.41	0.0011
103.63	0.0004
103.45	0.0032
103.43	0.2635
102.54	0.0323
102.25	0
102.1	0.1197
101.94	0.3701
101.66	0
101.31	0.0492
101.27	0
101.12	0
100.63	0.0034
100.58	0
100.5	0
100.41	0.0198
100.4	0.0128
100.02	0.0021
100	0.0327
99.78	0

$Zn^{+}(C_2H_4)$ Isomer 5
MN15-L
m=4

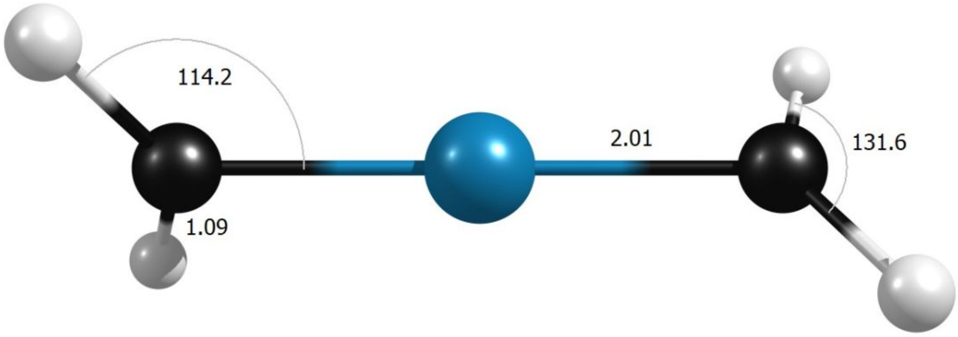

Coordinates:

30	0.000001000	-0.000058000	0.000065000
6	-2.010684000	0.000129000	-0.000142000
6	2.010678000	0.000106000	-0.000112000
1	-2.458759000	0.705604000	0.703750000
1	-2.458745000	-0.705526000	-0.703862000
1	2.458868000	-0.703832000	0.705245000
1	2.458627000	0.704097000	-0.705567000

Zero-Point Corrected Electronic Energy:
-1857.412295 Hartrees

Vibrational Frequencies:

Frequency (cm^{-1})	Intensity (km/mol)
20.5069	1.5311
20.5391	1.5296
93.1167	0
268.3849	54.9898
268.3916	54.9707
369.1534	367.0304
430.0338	0
532.243	13.9082
532.2477	13.9036
1022.3459	1141.2122
1229.9244	0
3100.9417	180.467
3102.5058	0
3313.1369	67.8176
3313.1381	67.8001

Electronic Transitions:

Wavelength (nm)	Oscillator Strength
553.4	0.1515
261.29	0.0069
261.28	0.0069
251.96	0.0108
251.95	0.0108
232.97	0.0199
232.96	0.0199
198.5	0
197.58	0.0183
196.28	0
196	0
187.06	0
187.06	0
186.37	0
186.33	0
186.05	0.0734
166.41	0
159.03	0.0346
159.02	0.0346
158.18	0
158.04	0
152.71	0.5791
152.36	0.0074
152.36	0.0074
147.49	0
145.93	0.0001
145.92	0.0001
144.4	0
143.28	0.2665
140.55	0.0011
140.55	0.0011
135.79	0.1613
135.79	0.1613
132.79	0
132.78	0
131.65	0.0265
131.54	0
129.49	0.0676
129.49	0.0676

124.72	0.0007
124.72	0.0007
124.45	0
124.34	0.0026
122.11	0.0003
122.11	0.0003
121.24	0
121.17	0.021
121.16	0.021
120.86	0.0304
120.36	0.005
120.36	0.005
120.29	0
120.08	0.0287
119.95	0
118.73	0
115.8	0
115.73	0.0237
115.31	0
115.22	0.0087
115.22	0.0086
115.11	0
115.04	0.0001
115.03	0.0076
115.03	0.0076
114.59	0.0312
114.59	0.0311
112.06	0.0004
111.46	0.0001
111.46	0.0001
111.38	0.0477
111.02	0.0383
111.02	0.0383
109.81	0.0038
109.41	0
108.74	0
107.57	0
107.15	0.0011
106.78	0.0118
106.78	0.0118
106.58	0
106.15	0.0163
106.15	0.0163

105.7	0
105.47	0
104.94	0
104.89	0.0318
104.45	0
104.08	0.2242
103.52	0
103.25	0
103.08	0
102.42	0.0002
102.42	0.0002
101.51	0.0312
101.22	0.0001
101.22	0.0001
100.74	0
100.06	0.1597
99.96	0.0666
99.96	0.0666

$Zn^+(C_2H_4)$ (Symmetry Constrained C_{2v})
B3LYP
m=2

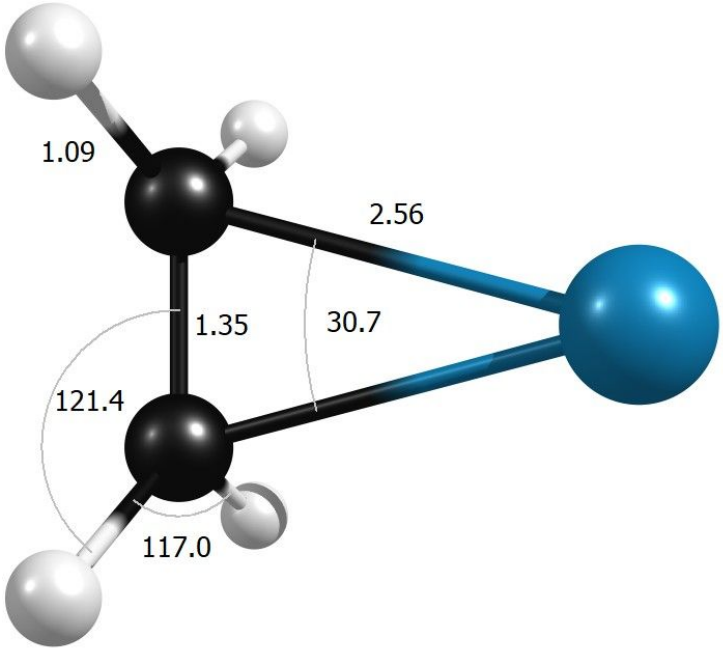

Coordinates:

6	0.000000000	0.676039000	-1.604689000
6	0.000000000	-0.676039000	-1.604689000
1	0.925147000	1.241332000	-1.650406000
1	-0.925147000	1.241332000	-1.650406000
1	0.925147000	-1.241332000	-1.650406000
1	-0.925147000	-1.241332000	-1.650406000
30	0.000000000	0.000000000	0.861930000

Zero-Point Corrected Electronic Energy:
-1857.725820 Hartrees

Vibrational Frequencies:

Frequency (cm^{-1})	Intensity (km/mol)
-88.7118	3.9779
184.9911	8.1838
324.5553	0.4598
839.916	0.615
1038.3038	0
1047.452	6.8347
1079.7775	104.7974

1247.902	0
1360.5989	40.6976
1480.5234	18.3237
1625.1859	52.8696
3123.9287	7.0804
3129.2179	0.0073
3208.1662	0
3229.9243	9.8823

Electronic Transitions:

Wavelength (nm)	Oscillator Strength
403.89	0.0054
295.82	0.1014
271.49	0.0906
253.33	0.0188
251.04	0
205.93	0.0458
193.41	0.5036
191.72	0
185.09	0.0073
175.49	0.0004
167.25	0.0416
162.99	0.3958
162.8	0.0006
157.04	0.0013
153.27	0.0004
152.56	0.0001
151.6	0.0002
151.6	0
151.44	0.0233
151.27	0.0264
149.59	0.0323
149.1	0.107
148.76	0.037
144.15	0.0011
143.8	0
141.8	0.0055
138.74	0.0013
136.06	0
135.84	0.0015
131.32	0
130.94	0.0013

130.01	0
129.16	0.0028
129.07	0.0158
128.24	0.0262
126.92	0.0136
126.4	0.0206
123.6	0
122.86	0
122.82	0
122.25	0.0166
122.16	0.0097
118.47	0
118.2	0.0006
116.7	0.0106
115.35	0.0153
115.11	0.0119
113.93	0.0003
113.81	0.0008
113.48	0
113.33	0.0125
113.24	0.0018
112.71	0.0005
112.58	0
112.52	0.0045
112.35	0.0028
112.11	0
111.95	0.0014
110.59	0.0006
110.57	0.0002
109.77	0.0001
109.63	0
109.54	0
109.38	0
109.18	0.0462
109.13	0.0085
109.09	0.0085
108.58	0.0036
107.83	0
107.22	0.0016
107.02	0.0012
106.62	0.0045
106.48	0.0505
105.13	0.0004

104.1	0.0059
104.1	0.002
103.98	0.0043
103.81	0.0034
103.8	0
103.32	0
103.24	0.0011
102.43	0.0942
101.96	0.0592
101.88	0.0269
101.81	0.1475
101.72	0.1421
101.16	0
100.83	0.0004
100.43	0.0048
99.99	0.0107
99.59	0
99.28	0.0007
98.92	0.0318
98.82	0.0334
98.48	0.0108
98.06	0
97.58	0.0141
97.5	0.0001
97.19	0.0004
96.91	0.005

$Zn^{+}(C_2H_4)$ (Symmetry Constrained C_{2v})
M06
m=2

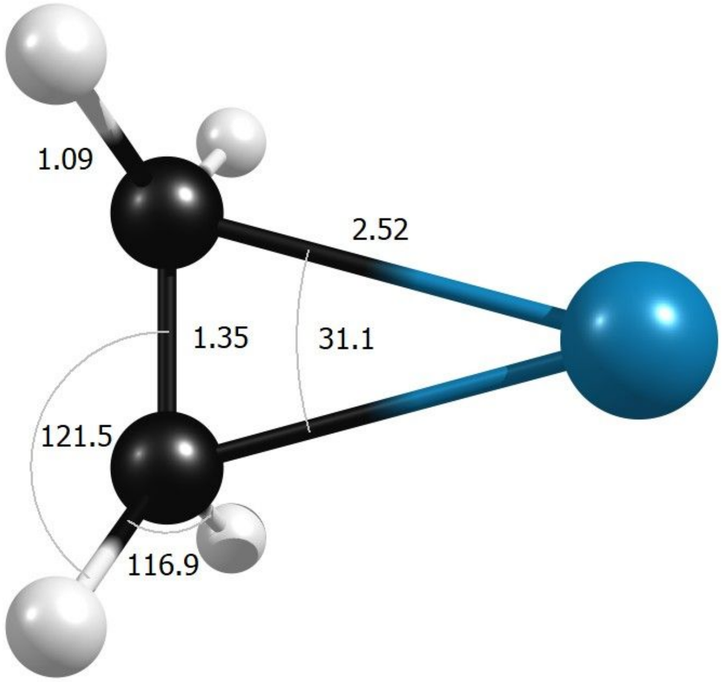

Coordinates:

6	0.000000000	0.674359000	-1.576652000
6	0.000000000	-0.674359000	-1.576652000
1	0.925739000	1.241303000	-1.619015000
1	-0.925739000	1.241304000	-1.619015000
1	0.925739000	-1.241304000	-1.619015000
1	-0.925739000	-1.241303000	-1.619015000
30	0.000000000	0.000000000	0.846530000

Zero-Point Corrected Electronic Energy:
-1857.560131 Hartrees

Vibrational Frequencies:

Frequency (cm^{-1})	Intensity (km/mol)
-96.7364	3.8017
197.237	6.1309
305.0572	0.3605
812.4544	0.3132
1021.2994	0
1024.1798	7.7463

1057.4199	89.0254
1212.7004	0
1342.1314	35.1049
1436.5497	18.6243
1609.517	72.3718
3110.1325	10.8145
3116.2523	0.0023
3204.93	0
3224.9679	13.6297

Electronic Transitions:

Wavelength (nm)	Oscillator Strength
410.78	0.0018
307.68	0.1157
259.4	0.0753
252.72	0
242.77	0.0118
203.63	0.0476
192.88	0.3482
190.99	0
183.77	0.003
179.85	0.1201
179.35	0.0007
166.29	0.2053
165.26	0.0017
161.2	0.0143
158.74	0.1926
156.36	0.0013
152.91	0.0043
149.88	0.0042
149.62	0.0081
149.4	0.0002
149.39	0
148.83	0.1426
147.64	0
144.86	0.0018
144.84	0.0179
143.92	0
142.62	0.0058
142.48	0
139.93	0.0012
137.61	0.0042

136.17	0.0003
134.97	0.0072
134.25	0.0599
133.39	0.0093
130.48	0.0099
129.8	0.0104
129.76	0
129.73	0.0039
124.53	0
124.14	0.0039
123.6	0
122.01	0.0145
121.37	0.0024
120.56	0.0005
120.5	0.0012
120.36	0.0347
119.92	0
119.37	0
118.08	0.0013
117.89	0.0008
117.01	0
116.65	0.0205
114.51	0.0015
113.89	0.0005
113.48	0.0004
112.72	0.0012
112.57	0.0014
111.7	0.0044
111.21	0.0004
109.66	0.004
109.52	0.0069
109.44	0.0003
109.23	0
109.14	0
108.88	0.0003
108.77	0.0003
108.46	0.0022
108.06	0.0085
107.44	0.0008
107.12	0.1288
107	0.0018
106.86	0
106.77	0.0293

106.69	0.0094
106.55	0
106.46	0.0367
106.28	0.0047
106	0.0839
105.23	0.0566
104.87	0.0088
104.55	0.0118
103.54	0.0004
103.16	0.0345
103.06	0
102.27	0
101.93	0.0378
101.9	0.0012
101.74	0.0008
101.65	0.0185
101.26	0
100.87	0.0007
100.78	0.001
100.76	0.0001
100.64	0.0361
100.45	0.0053
100.42	0.0001
99.74	0.0006
99.45	0
99.4	0.0003
98.96	0.0033

$Zn^+(C_2H_4)$ (Symmetry Constrained C_{2v})
M06-L
m=2

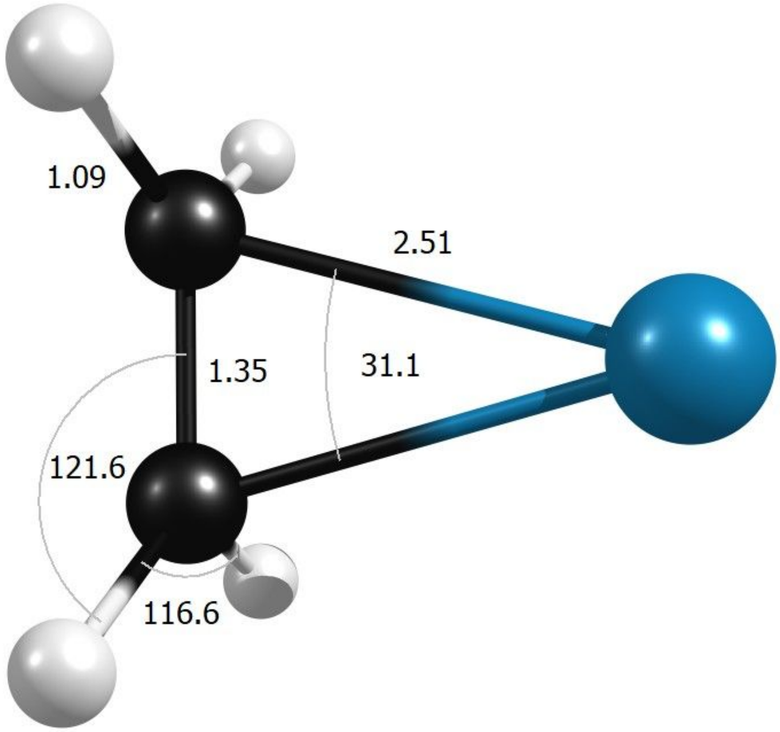

Coordinates:

6	0.000000000	0.673587000	-1.574428000
6	0.000000000	-0.673587000	-1.574428000
1	0.923288000	1.242545000	-1.616484000
1	-0.923288000	1.242545000	-1.616484000
1	0.923288000	-1.242545000	-1.616484000
1	-0.923288000	-1.242545000	-1.616484000
30	0.000000000	0.000000000	0.845303000

Zero-Point Corrected Electronic Energy:
-1857.597201 Hartrees

Vibrational Frequencies:

Frequency (cm^{-1})	Intensity (km/mol)
-121.1881	3.5525
189.2586	8.3541
286.7619	0.3913
824.8915	0.3044
1018.4862	6.6113

1052.1663	0
1067.035	89.51
1233.6395	0
1361.9634	29.8118
1459.0526	13.2102
1623.5667	53.6228
3123.5502	8.2525
3130.9068	0.2659
3218.7535	0
3240.7065	9.4924

Electronic Transitions:

Wavelength (nm)	Oscillator Strength
426.5	0.0055
287.31	0.1017
263.53	0.1053
258.91	0
246.63	0.0165
214.06	0.0519
191.46	0
188.07	0.1347
185.42	0.3378
176.56	0.0003
165.3	0.002
163.37	0.0142
163	0
162.95	0.0005
162.47	0.0114
161.41	0.3474
160.66	0.0674
159.53	0.0045
154.64	0.0015
152.33	0.0015
152.09	0.0021
148.46	0.0407
148.15	0.1447
146.44	0.0066
141.41	0.0036
140.96	0.0016
138.76	0.002
133.88	0.0045
132.01	0.0011

131.19	0
129.86	0.001
128.22	0.0083
128.19	0.0006
128.04	0
127.92	0.0083
125.91	0.0021
125.77	0
125.22	0.0033
124.52	0.0003
124.52	0.0016
124.37	0.0071
123.91	0.0059
123.32	0
123.17	0.0067
122.97	0.022
122.77	0.0031
122.73	0.0016
121.99	0.016
121.44	0
120.77	0.1006
120.2	0
120.09	0
117.82	0
117.48	0.0212
116.96	0.0437
114.75	0.0005
114.13	0.0016
113.36	0.0008
112.3	0
112.18	0.0094
111.75	0.001
111.33	0
110.97	0.0011
109.91	0.0008
109.42	0.0008
109.04	0.0219
108.95	0
108.4	0.0015
108.4	0
108.18	0
107.48	0.0001
106.64	0

106.3	0
105.9	0.0594
105.57	0.0011
105.57	0.0027
105.08	0.0006
105.05	0.0161
104.86	0.0095
104.78	0.0544
104.48	0.0098
104.45	0.0001
103.94	0
103.9	0.0001
103.22	0.0001
102.67	0.1003
102.44	0.1368
102.35	0.0001
101.73	0.1263
101.35	0.0015
100.15	0.0008
100.05	0.0001
99.3	0
98.75	0.0001
98.62	0.0024
98.4	0
97.52	0.0027
97.46	0.0033
97.12	0
97.11	0.0046

$Zn^+(C_2H_4)$ (Symmetry Constrained C_{2v})
MN15-L
m=2

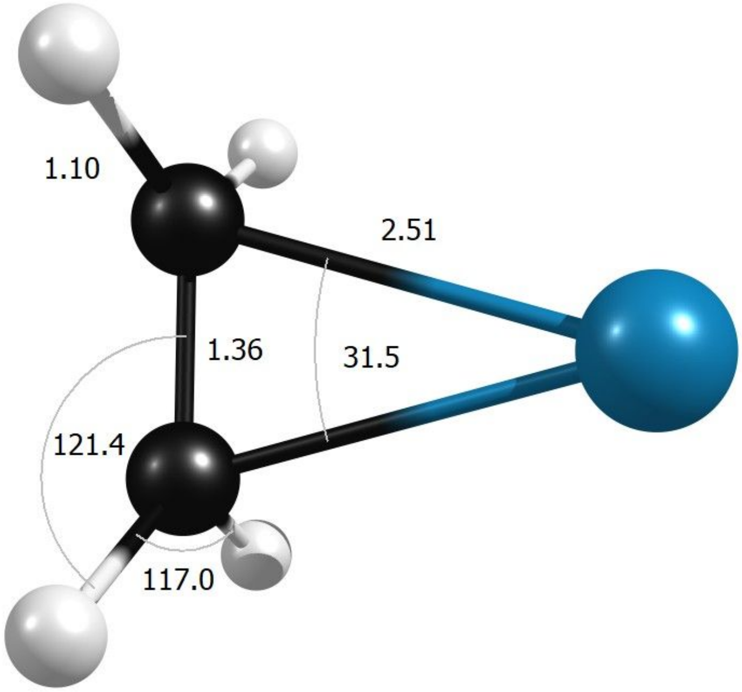

Coordinates:

6	0.000000000	0.680924000	-1.569000000
6	0.000000000	-0.680924000	-1.569000000
1	0.935797000	1.252739000	-1.606049000
1	-0.935797000	1.252739000	-1.606049000
1	0.935797000	-1.252739000	-1.606049000
1	-0.935797000	-1.252739000	-1.606049000
30	0.000000000	0.000000000	0.841740000

Zero-Point Corrected Electronic Energy:
-1857.571319 Hartrees

Vibrational Frequencies:

Frequency (cm^{-1})	Intensity (km/mol)
-56.7678	4.6737
192.7325	11.4629
295.1079	1.4209
816.4582	0.1989
1017.6282	4.3988
1069.1324	0

1079.4861	113.2174
1241.2588	0
1366.1399	35.8574
1466.763	11.7525
1618.3329	50.0003
3110.3662	7.3019
3120.4776	0.1789
3207.0839	0
3231.0277	8.6332

Electronic Transitions:

Wavelength (nm)	Oscillator Strength
434.97	0.0135
294.29	0.1134
268.14	0.0972
254.43	0.0205
229.77	0
216.35	0.0564
189.92	0.4799
184.5	0
172.23	0.0005
171.45	0.0051
169.56	0.0007
167.11	0.0754
162.71	0.3748
153.86	0.0202
152.28	0.0124
151.53	0.01
150.22	0.0112
150.19	0
150.17	0.0085
150	0.1262
149.69	0.0004
149.18	0.049
145.13	0
143.4	0.0051
142.36	0.0002
139.86	0.0108
139.05	0.0015
136.8	0.0004
135.36	0.0032
132.68	0

131.85	0.0008
130.53	0.0196
128.9	0.002
128.59	0.0029
128.46	0.0387
126.46	0.0229
126.33	0
125.26	0.01
123.57	0.0102
122.95	0.0006
122.83	0.0026
122.69	0
122.14	0.0035
121.32	0
121.31	0.0002
120.95	0
119.99	0.014
119.69	0.0007
119.66	0.0031
119.36	0.0131
119.2	0.0134
118.23	0.0588
117.02	0
116.68	0
115.74	0.0034
115.19	0.0463
113.46	0.0009
112.95	0
112.23	0.0164
112.02	0.0037
111.29	0.002
110.9	0.0377
110.8	0
110.71	0
109.96	0.0114
109.35	0.0046
108.68	0.0027
108.59	0
108.27	0.0011
107.93	0.001
107.89	0.0015
107.56	0
107.02	0.0031

106.64	0.0154
105.73	0.0033
105.44	0.0013
104.93	0.0068
104.48	0.0396
103.99	0.0011
103.91	0.0579
103.43	0.0202
103.13	0.0037
102.84	0
102.5	0.0001
102.37	0.0223
102.03	0
101.9	0.2337
100.98	0.1574
100.78	0.0366
100.55	0.001
99.95	0.0003
99.73	0
99.25	0.0121
98.74	0.0005
98.46	0.0033
98.37	0.0029
98.1	0
97.72	0.0011
97.34	0
96.9	0.0004
